# Contribution of the Tumor Microenvironment to Metabolic Changes Triggering Resistance of Multiple Myeloma to Proteasome Inhibitors

**DOI:** 10.3389/fonc.2022.899272

**Published:** 2022-05-26

**Authors:** Jonas Schwestermann, Andrej Besse, Christoph Driessen, Lenka Besse

**Affiliations:** Laboratory of Experimental Oncology, Clinics for Medical Hematology and Oncology, Cantonal Hospital St. Gallen, St. Gallen, Switzerland

**Keywords:** multiple myeloma, tumor microenvironment, proteasome inhibitors, resistance, metabolism

## Abstract

Virtually all patients with multiple myeloma become unresponsive to treatment with proteasome inhibitors over time. Relapsed/refractory multiple myeloma is accompanied by the clonal evolution of myeloma cells with heterogeneous genomic aberrations, diverse proteomic and metabolic alterations, and profound changes of the bone marrow microenvironment. However, the molecular mechanisms that drive resistance to proteasome inhibitors within the context of the bone marrow microenvironment remain elusive. In this review article, we summarize the latest knowledge about the complex interaction of malignant plasma cells with its surrounding microenvironment. We discuss the pivotal role of metabolic reprograming of malignant plasma cells within the tumor microenvironment with a subsequent focus on metabolic rewiring in plasma cells upon treatment with proteasome inhibitors, driving multiple ways of adaptation to the treatment. At the same time, mutual interaction of plasma cells with the surrounding tumor microenvironment drives multiple metabolic alterations in the bone marrow. This provides a tumor-promoting environment, but at the same time may offer novel therapeutic options for the treatment of relapsed/refractory myeloma patients.

## Introduction

Multiple myeloma (MM) is a plasma cell (PC) malignancy that is characterized by clonal expansion of malignant PCs inside the bone marrow (BM). Excessive production of monoclonal immunoglobulins (Igs) together with complex interactions with other members of the BM microenvironment (BMM) lead to pathological complications including bone lesions, hypercalcemia, renal failure, cytopenia and immunodeficiency at the time of MM diagnosis (1). Despite the development of novel and biology-driven anti-MM drugs in the past two decades, disease heterogeneity, early relapse and treatment resistance still pose major challenges in MM therapy. Moreover, subclonal heterogeneity of PCs evolves alongside disease progression through selection of increasingly drug-resistant as well as genetically and metabolically adapted subclones (2, 3). Nowadays, immunomodulatory drugs (IMiDs), immunotherapies based on monoclonal antibodies (mABs), and proteasome inhibitors (PIs) constitute an integral part of MM treatment regimens and have considerably improved patient prognosis. However, patients who are triple-class refractory towards IMiDs, mABs and PIs only have 5.6 months median overall survival (4), emphasizing the need to understand the underlying mechanisms that mediate (multi-)drug resistance in MM.

Since MM PCs secrete immense amounts of Igs, they are highly dependent on their ability to dispose of misfolded proteins *via* proteasomal degradation. Approximately 90% of total protein degradation occurs *via* the ubiquitin-proteasome system. In addition, MM PCs heavily rely on the unfolded protein response (UPR) and the endoplasmic reticulum (ER)-associated degradation (ERAD) machinery to ensure adequate protein folding and turnover to maintain cellular proteostasis (5). Proteasomes are proteolytic complexes that degrade ubiquitinated proteins and are composed of a 20S core catalytic particle and a 19S regulatory particle. The 20S particle has three distinct catalytic sites: the chymotrypsin-like site (β5 subunit), the trypsin-like site (β2 subunit) and the caspase-like site (β1 subunit) (6). PIs, such as Bortezomib (BTZ), Carfilzomib (CFZ), and Ixazomib, are selective inhibitors that by design bind to the β5 catalytically active site of proteasomes and inhibit its activity (7). Notably, at higher concentrations, BTZ also co-inhibits the β1 subunit, whereas CFZ co-inhibits the β2 subunit (6, 7), thus providing a slightly different scenario of proteasome inhibition, likely contributing to different clinical outcomes of treatment with the drugs. Proteasome inhibition causes excessive accumulation of (misfolded) proteins within MM cells, leading to prolonged and irresolvable ER/proteotoxic stress, and apoptosis (8, 9). Although the PI drugs are initially very effective, the evolving resistance and disease progression in relapsed/refractory MM (RRMM) remains a long-term clinical challenge. The biology of PI-resistant MM is currently being dissected in some detail (10–12). Increasing evidence suggests a metabolic rewiring as a cell biological basis of the adaptation of MM cells to PIs at the sub-clonal level (13).

In recent years, accumulating evidence has also emphasized the importance of the BMM for MM pathogenesis, cell growth, survival, migration, and drug resistance (14). The BMM is composed of a cellular and a non-cellular compartment and MM PCs strongly interact with both compartments in a mutual fashion. Such interactions are regulated in an autocrine and/or paracrine fashion and induce proteomic and metabolomic changes in MM and other BM resident cells, thereby creating a hypoxic, nutrient depleted, and tumor supportive microenvironment. Thus, not surprisingly, due to the supportive and protective contribution of the tumor microenvironment (TME) and metabolic rewiring of MM PCs, the therapy of RRMM remains difficult (15–17).

In this review, we summarize the key players involved in TME-mediated PI resistance and delineate contact dependent and contact independent interactions between them and MM PCs. Moreover, we describe proteomic and metabolomic reprogramming of MM cells within the TME, elucidate the metabolic consequences of proteasome inhibition and metabolism-related factors promoting PI resistance in MM in the context of the TME and further present potential strategies on how to overcome TME-mediated PI resistance.

## The Tumor Microenvironment in Multiple Myeloma

Malignant transformation of normal PCs to MM is not only a result of molecular changes of the cells themselves but is likewise influenced by the surrounding BMM and its interactions with the malignant PCs. The BMM surrounding malignant PCs during active disease, also called a TME, is a sophisticated network of cells of hematopoietic origin (including myeloid cells, T- and B-lymphocytes, natural killer (NK) cells, osteoclasts, etc.), or mesenchymal origin (mesenchymal stromal cells, fibroblasts, osteoblasts, adipocytes, endothelial cells (ECs)). The non-cellular compartment comprises the extracellular matrix (ECM) and the liquid BM milieu, including soluble factors such as cytokines, growth factors and chemokines, which are produced and/or affected by the cellular compartment of the BMM. Malignant PCs constantly interact with their surrounding TME thereby gaining access to a wide array of TME-derived pro-survival signals, which help them thrive within the BM niche. Moreover, molecular changes occurring during the progression of MM in malignant PCs and within the TME culminate in an expansion of malignant PCs throughout the BM. At the same time, soluble factors and physical interaction with other BM-homing cell types mediate drug resistance of PCs in several settings. The following sections will shed some light onto key cellular and soluble compartments of the TME, which are associated with disease pathogenesis and progression, and their interaction within the TME.

### Key Players of the Myeloma TME

#### Bone-Marrow Mesenchymal Stromal Cells

BM-derived mesenchymal stromal cells (BMSCs) are multipotent cells located within the BM stroma that are required for bone development, homeostatic remodeling, and repair (18). They can differentiate into various cell lineages, such as adipocytes, osteoblasts, fibroblasts, ECs, pericytes and neuronal cells. Together they form the skeletal structure of the BM and generate a permissive environment that influences the function and differentiation of hematopoietic cells. In MM, BMSCs strongly interact with malignant PCs in a reciprocally supporting manner towards cancer progression (19).

#### Osteoclasts

Osteoclasts are multinucleated monocyte-macrophage derivatives that degrade bone, and thus are involved in periodic repair and remodeling of bone tissue. Osteoclasts dissolve bone mineral by massive acid secretion and secrete specialized proteinases to degrade the organic protein matrix, which is mainly composed of type I collagen. In myeloma, osteoclasts are heavily activated due to various soluble factors secreted by MM cells and BMSCs, which ultimately leads to bone lesions, a hallmark of MM (20, 21).

#### Osteoblasts

Osteoblasts are specialized, terminally differentiated BMSCs that synthesize dense, crosslinked collagen as well as specialized proteins, such as osteocalcin, osteopontin and hydroxyapatite, which are essential components of the bone matrix. Physiologically, osteoblasts and BMSCs both produce osteoprotegerin (OPG), which counteracts bone resorption and further prevents osteoclast maturation and activation (22, 23). In MM, OPG has been shown to be bound, internalized and degraded by MM cells (24). Thus, MM cells inhibit osteoblast formation and differentiation, resulting in bone loss (25, 26).

#### Bone Marrow Endothelial Cells

ECs line the interior surface of blood vessels (vascular ECs) and lymphatic vessels (lymphatic ECs). ECs form the barrier between vessels and tissue and allow for the exchange of nutrients, hormones, or catabolites as well as the transit of white blood cells into and out of the surrounding tissue. At the same time, ECs are involved in processes such as inflammation, angiogenesis, and blood pressure control. BM angiogenesis is a hallmark of MM progression (27, 28). Induction of pro-angiogenic genes and secretion of growth factors and matrix metalloproteinases (MMPs) by MM cells facilitate ECs growth and neovascularization of the TME (29–32). Moreover, a progressive increase in BM microvascular density correlates with the development of PC disorders, ranging from monoclonal gammopathy of undetermined significance (MGUS) to smoldering (SMM) and active MM (28).

#### Bone Marrow Adipocytes

BM adipocytes (BMAds) are derived from BMSCs and constitute the majority of BM adipose tissue, a type of fat deposit in the BM. BM adipose tissue expands with aging and obesity, two well-known MM risk factors, suggesting that BMAds play a role in MM pathogenesis. BMAds contribute to systemic metabolism *via* secretion of circulating adipokines (cytokines secreted by adipose tissues) as well as free fatty acids and are involved in processes such as bone remodeling and hematopoiesis. Specifically, the MM-associated adipocytes exhibit reduced adipogenic gene expression and lipid loss and support MM cell growth and resistance to Dexamethasone-induced cell cycle arrest and apoptosis (33). At the same time, MM-induced BMAd-derived exosomal lncRNA mediate resistance towards BTZ, CFZ and Melphalan *via* apoptosis inhibition (34). The precise role of BMAds in MM needs to be further elucidated, as obesity is associated with increased risk of MGUS development and progression to MM (35, 36); however, being underweight is a risk factor of mortality in newly diagnosed MM (37).

#### Bone Marrow Immune Cells

A general alteration of the immune system, a condition termed immunosuppression, is a common characteristic of MM patients, that has been associated with disease evolution from its precursor stages (38). In the context of the BM niche, complex interactions between immune cells and MM PCs shift the balance towards an immunosuppressive environment (39). It is characterized by high concentration of immunosuppressive factors, loss of effective antigen presentation, effector cell dysfunction and expansion of immunosuppressive cell populations, such as myeloid-derived suppressor cells (MDSCs), regulatory T cells (T_reg_), tumor-associated macrophages, Th17 cells and T cells expressing checkpoint molecules, all together promoting myeloma progression (40–42). Recent studies have shown that malignant transformation of PCs is associated with altered expression of HLA class I antigen processing machinery (APM) components, and further downregulated expression of proteasome subunits (43). These changes lead to decreased expression of tumor antigen peptides on the PCs surface, enabling MM cells to evade CD8^+^ T cell recognition and killing (44, 45). Whether alterations in the immune system are responsible for disease progression to MM, or in contrast, abnormalities in the malignant PCs induce an immunosuppressive microenvironment, favoring the transition from SMM to active MM is still a matter of debate.

### Interaction of the TME With MM Cells

#### Contact Dependent Interaction

##### Cell Adhesion/Cell-to-Cell Interaction

**Figure 1:**
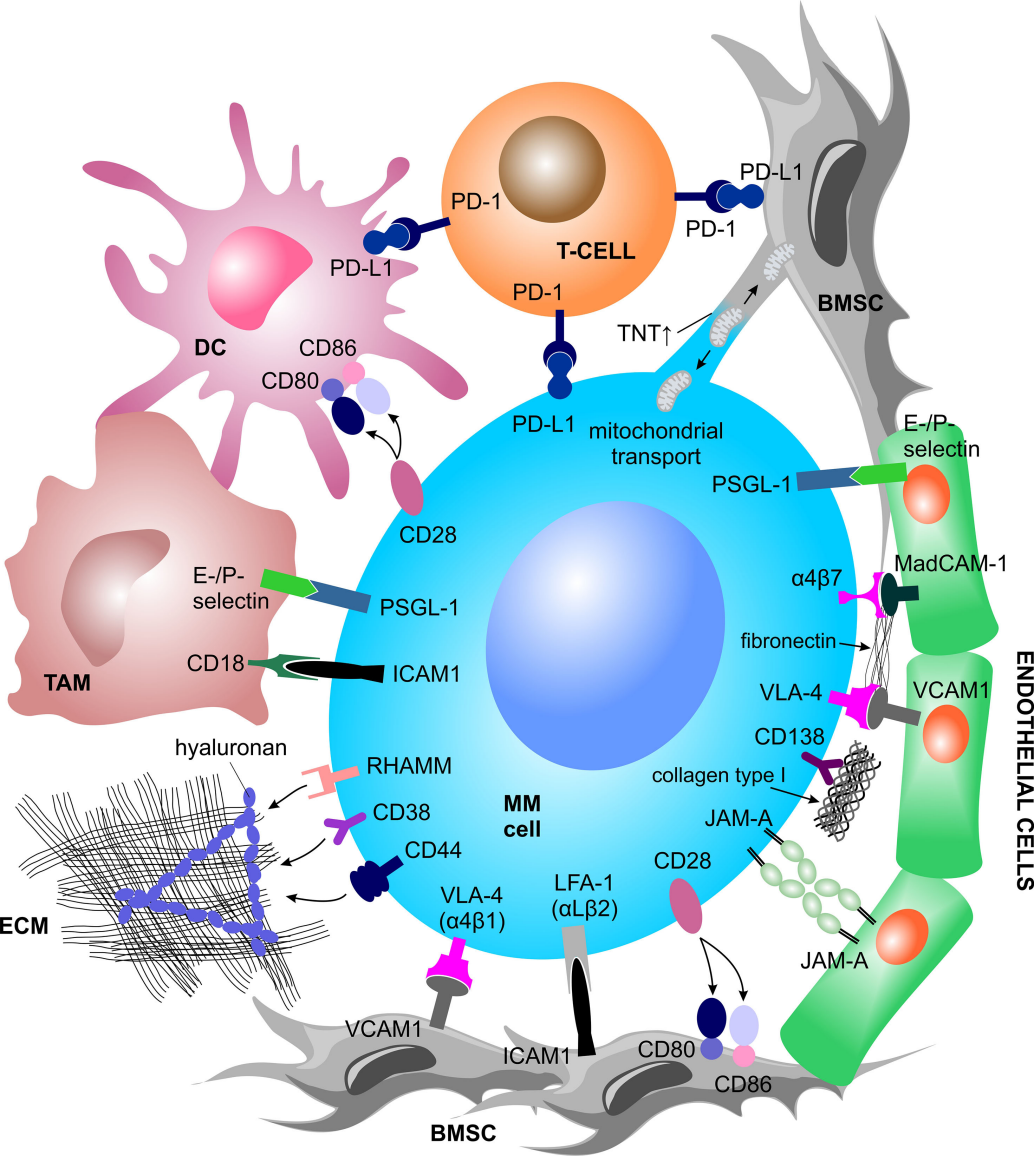
Contact dependent interactions between MM cells and the TME. MM cells physically interact with various other BM-homing cell types and the ECM structures *via* receptor-ligand binding or tunneling nanotubes. Such interactions trigger a plethora of inter- and intracellular signal cascades which support MM cell growth and metabolism, immune evasion and therapy resistance. BMSC, bone marrow stromal cell; DC, dendritic cell; ECM, extracellular matrix; ICAM-1, intercellular adhesion molecule 1; JAM-A, junctional adhesion molecule A; LFA-1, lymphocyte function-associated 1; MAdCAM-1, mucosal vascular addressin cell adhesion molecule 1; MM, multiple myeloma; PD-1, programmed cell death protein 1; PD-L1, programmed cell death 1 ligand 1; PSGL-1, P-selectin glycoprotein ligand-1; RHAMM, receptor for hyaluronan mediated motility; TAM, tumor associated macrophage; TNT, tunneling nanotubes; VCAM1, vascular cell adhesion molecule 1; VLA-4, very late antigen-4.

An important aspect of the TME in MM is cellular crosstalk mediated by cell-to-cell interaction *via* receptor-ligand binding. Virtually all cell types present within the BM can interact with MM PCs and directly support MM growth and metabolism, immune evasion and therapy resistance (**Figure 1**). The cellular interaction induces downstream signaling, ultimately triggering the release of soluble factors into the TME, or direct exchange of mitochondria through tunneling nanotubes (TNT).

###### Interaction With BMSCs

Myeloma cells interact with BMSCs *via* binding of very late antigen-4 (VLA-4), also known as α4β1 integrin, to vascular cell adhesion molecule 1 (VCAM1) or *via* binding of lymphocyte function associated-1 (LFA-1), also known as integrin subunit ß2, or Mucin 1 (MUC1, cell surface associated) to intercellular adhesion molecule 1 (ICAM-1) (46). BMSC-MM cell-to-cell interaction triggers downstream signaling cascades in both cell types, which ultimately increases MM cell fitness. Therefore, perhaps not surprisingly, high LFA-1 is associated with poor prognosis in MM patients and in mice (47, 48), and presence of MUC1 promotes MM proliferation (49). While in MM this interaction activates nuclear factor kappa B (NFκB) signaling, a major driver of MM survival and proliferation (50), in BMSCs, it induces the activation of the mitogen-activated protein kinase (MAPK), Notch, and phosphoinositide 3-kinase (PI3K) pathways, which leads to the transcription and subsequent secretion of numerous cytokines (51).

###### Interaction With ECs

The interaction of MM PCs with ECs is implicated *via* P-selectin glycoprotein ligand 1 (PSGL-1) binding on MM PCs to E- and P-selectins on the surface of ECs, particularly during early cell adhesion (52, 53). Likewise, expression of adhesion molecules VLA-4, LFA-1 and CD44 on MM cells correlate with increased angiogenesis in active MM (54). Additionally, MM-associated ECs show elevated membrane expression of junctional adhesion molecule-A (JAM-A), which correlates with disease progression through enhancement of MM-associated angiogenesis. The interaction between MM-associated ECs and MM PCs increases the expression of JAM-A also on MM PCs surface (55).

###### Interaction With Immune Cells

Myeloma cells interact with surrounding immune cells *via* CD28 and programmed cell death 1 ligand 1 (PD-L1) molecules. Plasma and myeloma cells express CD28, a protein known for its role in providing co-stimulatory signals required for T cell activation and survival. MM cells retain CD28 expression due to its pro-survival capacity upon binding to CD80/CD86, which is expressed by BMSCs and dendritic cells (DCs) (56–58). Both CD28 and CD86 are essential for PCs development, myeloma survival and therapy resistance (57, 59, 60). At the same time, DCs interact with MM cells *via* CD80/CD86–CD28 interaction, promoting a downregulation of proteasome subunit expression and a consequent escape of MM cells from CD8+ T cell recognition and killing (45). Moreover, plasmacytoid DCs promote MM cell growth, survival, and drug resistance (43) and express high surface levels of PD-L1 conferring T cell and NK cell immune suppression *via* the programmed cell death protein 1 (PD-1)–PD-L1 signaling axis (61, 62).

Within the MM-TME, PD-1 has been shown to be strongly expressed by γδ T cells (63) and NK cells (64) and to interact with PD-L1, expressed by myeloma PCs, DCs, and MDSCs thereby downregulating the immune response. Tumor associated macrophages are strongly represented in the TME. They have been shown to increase MM cell survival and protection from drug-induced apoptosis *via* contact-dependent interaction with MM PCs, involving PSGL-1 and ICAM-1 on MM PCs and E- and P-selectins and CD18 on the cell surface of macrophages (65–67). Myeloma cells can also directly induce formation of functional T_reg_ cells in a contact dependent manner, acting as immature and tolerogenic antigen presenting cells (APCs) (68), as well as in an independent manner *via* expression of the inducible co-stimulator ligand (ICOSL) (69).

A myriad of interactions between immune cells and MM cells are currently being studied towards identification of novel therapeutic approaches or are developed in the clinics as immunotherapy and immune-stimulating drugs (70, 71).

###### Interaction With Osteoblast Progenitors

MM cells co-cultured with human osteoblast progenitor cells exert inhibitory effects on osteocalcin, alkaline phosphatase, collagen I mRNA, protein expression, and RUNX family transcription factor 2 (RUNX2)/core binding factor alpha 1 (CBFA1) activity in osteoblast progenitor cells, thereby suppressing osteoblast formation and differentiation. Such inhibitory effects are partly driven by physical interaction between MM cells and osteoblast progenitor cells, involving the VLA-4–VCAM1 integrin system (25).

##### Mitochondrial Trafficking

In recent years, several studies have shown the importance of (bidirectional) mitochondrial trafficking through tunneling nanotubes (TNT) and partial cell fusions, and its association with increased growth potential, survival benefits, enhanced chemoresistance, as well as altered metabolism and functional properties of tumor cells (72–77). Specifically in MM, MM PCs endorse mitochondria uptake from autologous BMSCs once exposed to increasing concentrations of different chemotherapeutic drugs, thereby promoting MM survival and increasing the level of adenosine triphosphate (ATP) (*via* increased oxidative phosphorylation (OXPHOS) capacity), while lowering superoxide levels. These changes were proportional to the amount of incorporated BMSCs-derived mitochondria as well as the drug concentration but were independent of the type and mechanism of action of the applied drug. At the same time, autologous BMSCs incorporate the MM cell-derived mitochondria as well, which leads to increased levels of intracellular superoxides in BMSCs. In addition, the supportive effect of stromal cells could be successfully abrogated by the use of chemotherapeutic agents in combination with Metformin, an inhibitor of OXPHOS (77). Interestingly, mitochondrial trafficking appears to be a CD38-dependent process and shRNA-mediated knockdown of CD38 inhibited the trafficking and TNT formation *in vitro*, blocked the trafficking *in vivo* and improved survival of NSG mice that were engrafted with MM cell line with reduced CD38 expression (75).

MM-primed BMSCs have decreased reliance on mitochondrial metabolism as compared to healthy BMSCs, and increased tendency to deliver mitochondria to MM PCs. Particularly, PC-induced expression of connexin 43 (CX43) in BMSCs causing expression of C-X-C motif chemokine ligand 12 (CXCL12, also known as stromal-derived factor-1 alpha; SDF-1α) and consequent stimulation of its receptor C-X-C motif chemokine receptor 4 (CXCR4) on MM cells facilitates mitochondrial trafficking. An *in vitro* co-culture experiment showed that this interaction could be disturbed *via* selective inhibition of CXCR4 using Plerixafor, a CXCR4 antagonist, resulting in decreased mitochondrial transfer. In addition, the intracellular CXCR4 expression was elevated in CD138+ MM cells from MM patients who failed to respond to BTZ, suggesting that CXCR4 mediates chemoresistance in MM (76).

#### Contact Independent Interaction

##### Cytokine Signaling and Soluble Factors

**Figure 2:**
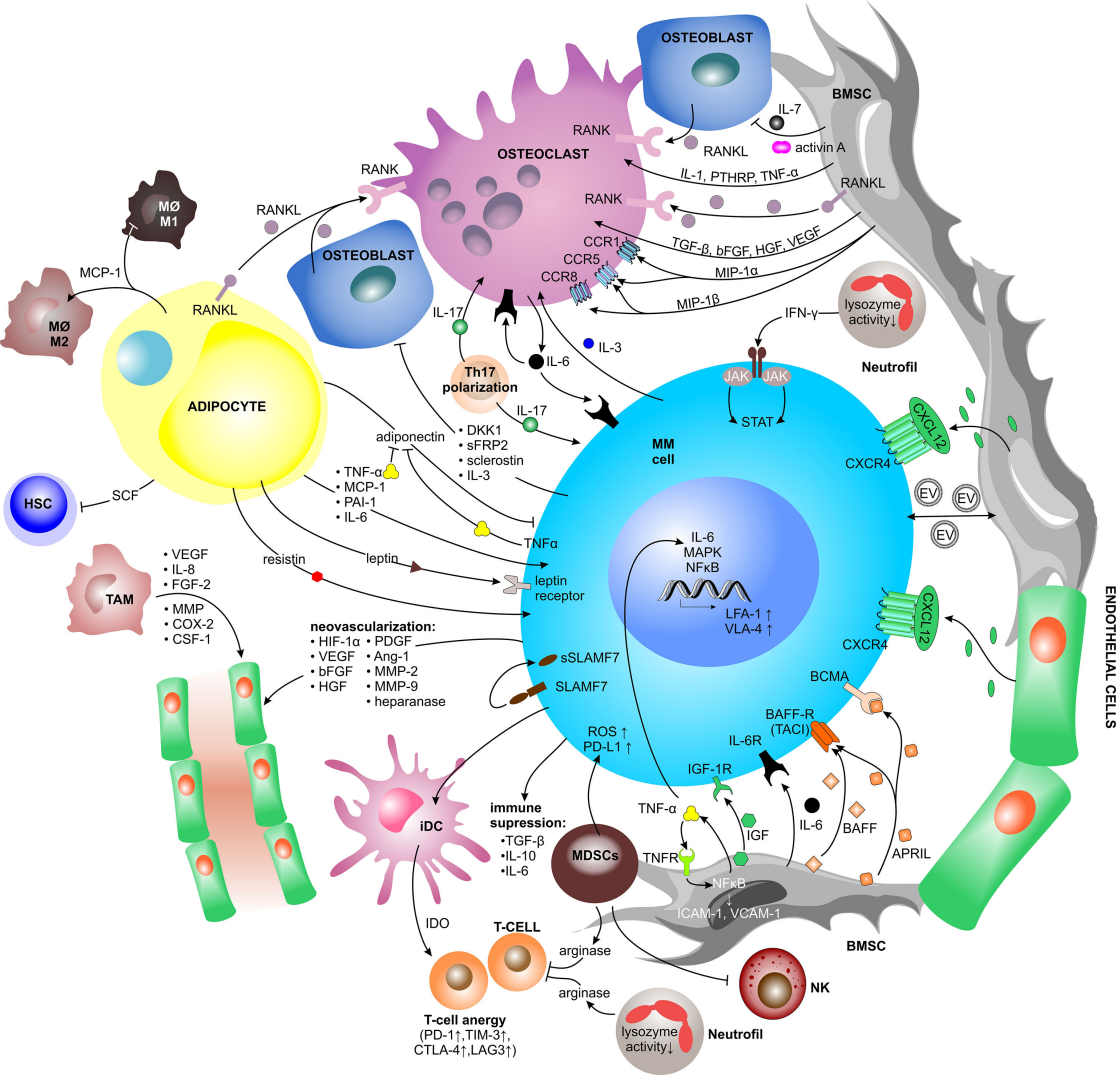
Contact independent interactions between MM cells and the TME. Cellular crosstalk between MM cells and other key players of the TME triggering the release of soluble factors (mainly cytokines, chemokines, and growth factors, but also extracellular vesicles). Upon binding to their cognate receptors, soluble factors trigger intracellular signaling pathways, which promote myeloma growth and survival as well as BM neovascularization, bone remodeling and immune evasion. Ang-1, angiopoietin-1; APRIL, a proliferation-inducing ligand; BAFF(-R), B-cell activating factor(-receptor); BCMA, B-cell maturation antigen; bFGF, basic fibroblast growth factor; BMSC, bone marrow stromal cell; CCR, C-C motif chemokine receptor; COX-2, cycloxygenase-2; CSF-1, colony stimulating factor-1; CTLA-4, cytotoxic T lymphocyte antigen-4; CXCL, C-X-C motif chemokine ligand; CXCR, C-X-C motif chemokine receptor; DC, dendritic cell; DKK1, dickkopf WNT signaling pathway inhibitor 1; EV, extracellular vesicle; FGF-2, fibroblast growth factor-2; HGF, hepatocyte growth factor; HIF-1α, hypoxia inducible factor-1α; HSC, hematopoietic stem cell; ICAM-1, intercellular adhesion molecule 1; iDC, immature dendritic cell; IDO, indoleamine 2,3-dioxygenase; IFN-γ, interferon-γ; IGF, insulin-like growth factor; IGF-1R, insulin-like growth factor 1 receptor; IL, interleukin; JAK2, janus kinase2; LAG3, lymphocyte-activation gene 3; LFA-1, lymphocyte function associated-1; MΦ, macrophage; MAPK, mitogen-activated protein kinase; MCP-1, monocyte chemoattractant protein-1; MDSC, myeloid-derived suppressor cell; MIP-1α/ß, macrophage inflammatory protein 1-α/ß; MM, multiple myeloma; MMP, matrix metalloproteinase; NFκB, nuclear factor kappa B; NK, natural killer cell; PAI-1, plasminogen activator inhibitor-1; PD-1, programmed cell death protein 1; PD-L1, programmed cell death 1 ligand 1; PDGF, platelet-derived growth factor; PTHRP, parathyroid hormone-related peptide; RANK(L), receptor activator of NFκB (ligand); ROS, reactive oxygen species; SCF, stem cell factor; sFRP2, secreted frizzled related protein 2; (s)SLAMF7, (secreted) signaling lymphocytic activation molecule family member 7; STAT, signal transducer and activator of transcription; TAM, tumor associated macrophage; TACI, transmembrane activator and CAML interactor; TGF-ß, transforming growth factor-ß; TIM-3, T-cell immunoglobulin and mucin-domain containing-3; TNF-α, tumor necrosis factors-α; VCAM1, vascular cell adhesion molecule 1; VEGF, vascular endothelial growth factor; VLA-4, very late antigen-4.

In the context of the TME, the direct ligand-receptor-mediated crosstalk between MM PCs and BM cellular compartment induces the release of soluble factors, mainly cytokines, growth factors and chemokines. Upon binding to their cognate receptors, these factors trigger intracellular signaling. Since all cells in the BM niche sense and respond to such stimuli, the soluble factors-mediated interaction stimulates MM PCs growth and survival, but at the same time promotes BM neovascularization, bone remodeling and immune evasion (**Figure 2**).

###### Factors Promoting MM Growth and Survival

Physical interaction between MM cells and BMSCs *via* VLA-4–VCAM1 binding induces BMSCs to produce multiple cytokines. One of them is interleukin-6 (IL-6), which is essential for MM growth, survival, migration, and drug resistance. It binds to its cognate receptor (IL-6R) and signals through mitogen-activated protein kinase (MEK)/MAPK, janus kinase (JAK)/signal transducer and activator of transcription (STAT), and PI3K/Akt pathways, leading to increase and stabilization of anti-apoptotic proteins, such as myeloid leukemia 1 (MCL-1) (50, 78–81). At the same time, MM PCs uniquely express the signaling lymphocytic activation molecule family member 7 (SLAMF7) receptor, which is cleaved *via* unknown mechanisms and detected as a soluble form (sSLAMF7) exclusively in the serum of MM patients (82). sSLAMF7 enhanced the growth of MM cells *via* homophilic interaction with surface SLAMF7 and subsequent activation of the downstream signaling pathways (83).

The BMSCs-derived cytokine B-cell activating factor (BAFF), a member of the tumor necrosis factor (TNF) family, is either expressed on the surface of BMSCs or it appears in a cleaved soluble form. Physiologically, BAFF stimulates B cell growth, and likewise binding of BAFF to its cognate BAFF-receptor (BAFF-R) or transmembrane activator and CAML interactor (TACI), on MM cells leads to increased proliferation and survival of MM (84, 85). Another BMSC-derived cytokine, a proliferation-inducing ligand (APRIL) can bind to TACI or B-cell maturation antigen (BCMA) on MM cells. APRIL and BCMA positively impact survival and growth of MM *via* MAPK and NFκB signaling and further promote immunosuppression *via* PD-L1, transforming growth factor-ß (TGF-ß), and IL-10 (86).

TNF-α is a well-described mediator of inflammation, which has recently been shown to be one of the main drivers inducing the inflammatory gene signatures in MM-associated BMSCs, which further promote MM PCs survival and immunomodulation within the TME (87). TNF-α has only a modest impact on the proliferative capacity of MM cells, but it induces expression of adhesion molecules resulting in a 2 to 4-fold increase in binding of MM cells to BMSCs (88). Moreover, TNF-α and IL-6 were significantly increased in the BM aspirates of patients with active MM. TNF-α triggers NFκB and MAPK activation, as well as secretion of IL-6 that is regulated by the JAK/STAT pathway (88, 89).

Similarly, MM cells influence BMSCs to produce growth factors which promote MM growth. A key growth factor promoting MM proliferation is insulin-like growth factor (IGF). IGF binds to its tyrosine kinase receptor, the insulin like growth factor 1 receptor (IGF-1R), and in this way supports MM growth, anti-apoptotic signaling and drug resistance to cytotoxic chemotherapy, Dexamethasone, and PIs (90). Moreover, IGF enhances the ability of MM cells to respond to other cytokines and to produce pro-angiogenic cytokines (91).

###### Factors Involved in Bone Remodeling

MM cells directly activate osteoclasts *via* secretion of macrophage inflammatory protein 1-α (MIP1-α), also known as C-C motif chemokine ligand 3 (CCL3), and MIP1-ß. Binding of MIP1-α to C-C motif chemokine receptor 1 (CCR1) and CCR5 and likewise binding of MIP1-ß to CCR5 and CCR8 both induce osteoclast formation and activity (21, 92, 93). MIP1-α further increases adhesion of MM cells to BMSCs and disease burden in immunodeficient mice suffering from MM (92, 93). In return, osteoclasts secrete IL-6 to stimulate the proliferation and growth of MM cells and other osteoclasts in an autocrine and paracrine fashion (94). Upon interaction, osteoclasts upregulate chondroitin synthase 1 (CHSY1), which induces Notch signaling promoting the survival of MM cells (95).

Apart from osteoclast activation, MM cells inhibit osteoblast differentiation *via* increased secretion of activin A into the microenvironment, leading to suppressed bone formation (96). Moreover, MM cells secrete cytokines, such as dickkopf WNT signaling pathway inhibitor 1 (DKK1), which is physiologically mainly produced by BMSCs and osteoblasts, secreted frizzled related protein 2 (sFRP2) and IL-3. The first two inhibit the canonical Wnt/ß-catenin pathway, which is responsible for osteoblast differentiation (25). IL-3 inhibits basal and bone morphogenic protein-2 (BMP-2)-stimulated osteoblast formation and plays an important role in stimulating osteoclast formation as well (97). Thus, DKK1, sFRP2, and IL-3 contribute to increased bone resorption in MM.

Beyond MM PCs, BMSCs as well produce osteoclastogenic cytokines such as IL-1, TNF-α and parathyroid hormone-related peptide (PTHRP) (20). The VLA-4–VCAM1 interaction also induces the production of BMSCs-derived receptor activator of NFκB ligand (RANKL), a membrane-bound or soluble (sRANKL) cytokine essential for osteoclast differentiation, which further increases osteoclast activation and bone lysis in MM. Importantly, the high sRANKL/OPG ratio is a negative predictor of survival in MM (20, 98) and ist therapeutic targeting with Denosumab, an anti-RANKL antibody, has been shown to reduce osteoclastogenesis and bone resorption markers in MM patients (99). Other factors increasing the sRANKL/OPG ratio are activin A and sclerostin, both produced by bone tissue (100, 101). Activin A, a member of the TGF-ß superfamily, is stored in bone tissue and is released from bone upon bone resorption. The increased level of circulating activin A causes downstream signaling through numerous pathways to promote osteoclast differentiation (101). At the same time, sclerostin, a secreted glycoprotein from the bone tissue, can be targeted therapeutically by Romosozumab, an anti-sclerostin antibody, which represents a potential new therapeutic strategy in MM bone disease (102, 103). In addition to increasing the RANKL/OPG ratio, BMSCs secrete IL-7, which has been shown to decrease RUNX2 activity and osteoblast differentiation (25, 104). Moreover, the growth factors produced and secreted by BMSCs, such as TGF-ß, hepatocyte growth factor (HGF), basic fibroblast growth factor (bFGF) and vascular endothelial growth factor (VEGF), are all involved in bone remodeling since they influence osteoclast activation and angiogenesis (97, 105).

###### Factors Promoting Neovascularization

Proliferating MM cells generate a hypoxic milieu within the TME and produce various pro-angiogenic modulators including hypoxia inducible factor-1α (HIF-1α), VEGF, bFGF, HGF, platelet-derived growth factor (PDGF), angiopoietin-1 (Ang-1), osteopontin, MMPs (MMP-2 and MMP-9), and heparanase, all of which contribute to EC proliferation and migration, ECM degradation, and neovascularization (29–32). At the same time, BMSCs, osteoclasts, osteoblasts and ECs secrete various pro-angiogenic and other factors, including VEGF, fibroblast growth factor-2 (FGF-2), TNF-α, HGF, IL-6 and IL-8, osteopontin, Ang-1, BAFF, CXCL12, and various Notch family members. All of these factors are up-regulated by physical and/or paracrine interactions with MM PCs (106). Heparanase-enhanced shedding of CD138, also known as syndecan-1 (SDC1), by MM PCs promotes endothelial invasion and angiogenesis (32). Moreover, tumor-associated macrophages contribute to MM-associated neovascularization *via* vasculogenic mimicry and indirectly *via* secretion of pro-angiogenic factors such as VEGF, IL-8, FGF-2, MMPs, cycloxygenase-2 (COX-2), and colony stimulating factor-1 (CSF-1) (107).

###### Immunosuppressive Factors Within the TME

The immunosuppressive TME in MM patients is caused by multiple immunosuppressive factors secreted by various cell lineages. The primary suspects, malignant PCs, are known to secrete TGF-ß, IL-10 and IL-6, all of which have debilitating effects on the immune system (108). At the same time, the interaction of PCs with immature DCs (iDCs) stimulates TGF-ß production by iDCs, subsequently inducing T_reg_ cell proliferation, which further increases TGF-ß and IL-10 levels within the microenvironment. Additionally, iDCs produce indoleamine 2,3-dioxygenase (IDO) that causes anergy in activated T cells (109). It results in upregulation of exhaustion markers in T cells, such as PD-1, cytotoxic T lymphocyte antigen-4 (CTLA-4), T cell immunoglobulin-3 (TIM-3), and lymphocyte-activation gene 3 (LAG3) as well as high levels of the T cell senescence markers, such as killer-cell lectin like receptor G1 (KLRG1) and CD160 (110). Within the TME, elevated levels of IL-6, TGF-ß, and IL-1ß promote T helper 17 cell (Th17) polarization, inducing the release of high levels of IL-17 favoring MM PCs growth and inhibiting the immune system (111–114). IL-17 also plays a role in osteoclast-mediated bone lysis (112).

MDSCs are known to suppress T cell-mediated immunity and thus help myeloma cells to escape from immunosurveillance (115, 116). MDSCs inhibit T cell activation and proliferation by secreting high levels of arginase, which sequesters L-arginine, an essential amino acid for T cell activity (117). At the same time, they induce reactive oxygen species (ROS) formation (118) and expression of PD-L1 on the surface of MM PCs (119). Moreover, MDSCs induce anergy of NK cells through membrane bound TGF-ß (120).

Myeloma-associated functional defects of neutrophils include reduced lysozyme activity (121) and increased secretion of arginase (122), which affects the T cells. Additionally, during disease progression neutrophils secrete increasing amounts of interferon-γ (IFN-γ) in response to MM soluble factors, thereby increasing MM JAK-2/STAT3 pathway activation that supports myeloma survival (123).

###### Adipokines

BMAds contribute to changes in systemic metabolism in MM *via* enhanced secretion of circulating adipokines (124) as well as cytokines, which regulate both bone remodeling *via* the production of RANKL (125) and hematopoiesis *via* stem cell factor (SCF) production (126). BMAd-derived adipokines include TNF-α, monocyte chemoattractant protein-1 (MCP-1), also known as CCL2, plasminogen activator inhibitor-1 (PAI-1), IL-6, resistin, adiponectin and leptin (127, 128). The anti-myeloma and anti-inflammatory cytokine adiponectin inhibits proliferation and induces cell death in MM cells (129). To protect themselves against the effect of adiponectin, MM cells downregulate adiponectin production *via* TNF-α secretion (130). On the contrary, leptin, another adipokine produced by BMAds, increases MM cell proliferation, reduces toxicity of PIs, and also counteracts the anti-tumor activity of invariant NK T (iNKT) cells, which express the leptin receptor (131, 132). Moreover, MM-associated adipocytes upregulate the expression of autophagy proteins in MM cells *via* leptin, leading to increased chemoresistance *in vitro* and *in vivo (* 133). MCP-1 is involved in transendothelial migration of MM cells and plays an important role as a chemoattractant essential for BM-homing (134). Moreover, MCP-1 promotes macrophage-associated chemoresistance in MM by shifting macrophages towards the M2-like phenotype (135). Further, it was shown that resistin induced multidrug resistance in MM by inhibiting cell death and upregulating the ABC transporter protein expression, leading to increased drug efflux (136).

##### Exosomes

Exosomes, a subtype of extracellular vesicles (EVs), are membranous vesicles (30–100 nm in diameter) of endocytic origin, which are generated in multivesicular endosomes (MVEs) and are released upon fusion of MVEs with the cell membrane (137). Exosomes are secreted by most cell types and act as carriers for intercellular transfer of nucleic acids, nucleoproteins (RNA, microRNA, DNA), enzymes, soluble factors, lipids and various other cytosolic molecules from parent to recipient cells, thereby inducing phenotypic and/or functional changes in the recipient cells (138–140). Since exosomes carry information and thus modulate the behavior of local and distant recipient cells, they are involved in a variety of physiological and pathological processes, such as malignant transformation and/or induction of the pre-metastatic niche (140, 141). Emerging evidence shows that MM-derived exosomes reprogram recipient cell functions in the BM to modulate and shape a pro-MM environment capable of supporting disease progression (140). Exosomes signaling is bidirectional and BMSC-derived exosomes (BMSC-EXs) have been found to induce MM growth, survival, and drug resistance (142). It has been shown that BMSC-EXs obtained from MM patients promoted MM growth, whereas BMSC-EXs from healthy individuals inhibited MM proliferation (143). Another study has shown that BMSC-EXs obtained from MM patients contain different cargo, such as lower levels of the tumor suppressor microRNA-15a and higher levels of IL-6, CCL2, and fibronectin, when compared to BMSC-EXs from healthy individuals. Thus, MM-BMSC-EXs, when delivered to MM cells, increase their proliferative capacity and survival (143). Emerging evidence shows that BMSCs selectively transfer specific proteins into MM cells that induce p38, p53, c-Jun N-terminal kinase (JNK), and Akt pathways to promote MM cell survival (144). It was also reported that BMSC-EXs (healthy or MM-derived) both induce upregulation of anti-apoptotic B-cell lymphoma-2 (Bcl-2) and downregulation of pro-apoptotic Caspase 9 and Caspase 3 in MM cells, thereby mitigating BTZ-induced apoptosis (144). Moreover, MM-derived exosomes contain and thus increase the levels of microRNA-146a in BMSCs, leading to enhanced secretion of several cytokines and chemokines by BMSCs, including CXCL1, IL6, IL-8, IP-10, also known as CXCL10, MCP-1, and CCL-5, resulting in enhanced MM cell viability and migration (145). In recent years, there has been a growing interest in understanding how exosomes contribute to MM pathogenesis and to further exploit their potential as prognostic, diagnostic and/or therapeutic tools in the treatment of MM (142).

### The Role of the ECM in MM

The BMM provides a three-dimension structure called the ECM, which consists of extracellular macromolecules, such as fibronectin, collagen, osteopontin, hyaluronan, laminin, enzymes, glycoproteins, and minerals, which provide structural and biochemical support to surrounding cells (146, 147). The ECM enables cell adhesion, cell-to-cell communication and differentiation (148). MM cells bind to ECM structures *via* activated VLA-4 and integrin subunit beta 7 (ITGB7), also known as integrin a4ß1 and integrin ß7, respectively (149, 150). At the same time, binding of VLA-4 to fibronectin induces activation of NFκB, leading to pro-survival signaling and cell adhesion mediated drug resistance (CAM-DR) (151). ITGB7 is constitutively active in MM cells and is essential for MM cell survival and CAM-DR (150, 152). Other integrins, such as VLA-5 and neural cell adhesion molecule (NCAM or CD56) or integrin ß5, are less essential, but still important in MM progression (149, 153). CD138 (SDC1) is a heparan sulfate proteoglycan and a surface marker of MM PCs. It binds to type I collagen and induces expression of MMP-1 to promote tumor invasion, bone resorption, and angiogenesis (105, 154). CD138 expression correlates with MM cell survival and growth and has been shown to promote myeloma progression *in vivo (* 155, 156). Additional ECM-binding proteins are CD44, receptor for hyaluronic acid-mediated motility (RHAMM) and CD38, all of which are receptors for the secreted scaffold protein hyaluronan. The first two also regulate the CXCL12–CXCR4 signaling axis (157). In summary, adhesion of myeloma cells to ECM structures has been shown to be important for survival and CAM-DR, e.g., towards anti-MM drugs such as BTZ, Vincristine, Doxorubicin and Dexamethasone (151, 157, 158).

## Crucial Factors Involved in Bone Marrow Homing of PCs

**Figure 3:**
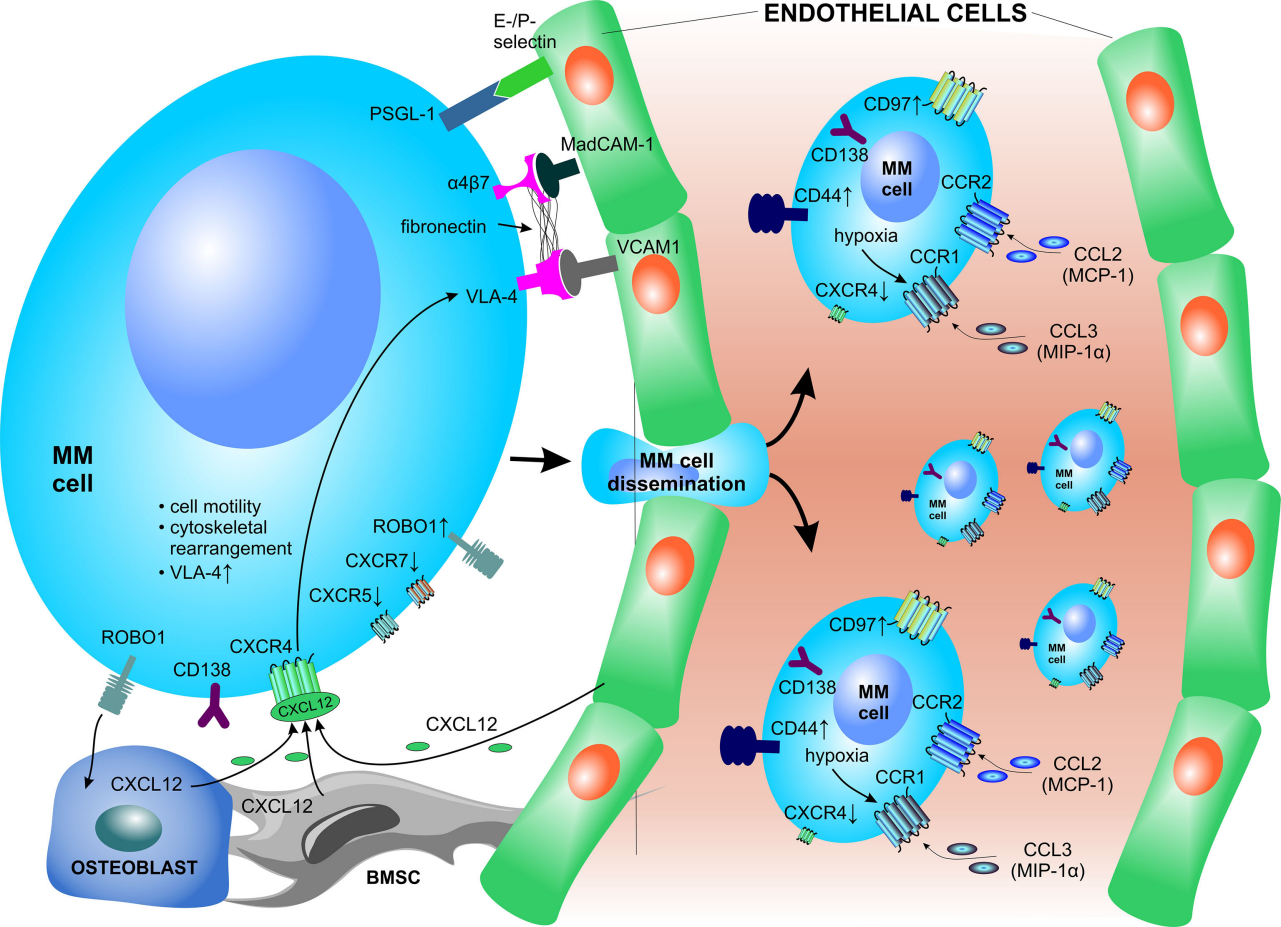
Crucial factors involved in bone marrow homing of plasma cells. PCs express several surface receptors which serve as environmental sensors but are also crucial for interactions with vascular structures and other components of the BM niche. The homing, lodging and retention of PCs to the BM is primarily mediated *via* the CXCL12–CXCR4 signaling axis. BMSC, bone marrow stromal cell; CCL, C-C motif chemokine ligand CCR, C-C motif chemokine receptor; CXCL, C-X-C motif chemokine ligand; CXCR, C-X-C motif chemokine receptor; MAdCAM-1, mucosal vascular addressin cell adhesion molecule 1; MCP-1, monocyte chemoattractant protein-1; MIP-1α, macrophage inflammatory protein 1-α; MM, multiple myeloma; PSGL-1, P-selectin glycoprotein ligand-1; ROBO1, roundabout guidance receptor 1; VCAM1, vascular cell adhesion molecule 1; VLA-4, very late antigen-4.

The homing, lodging and retention of PCs into the BM niche is primarily mediated by the PC-expressed chemokine receptor CXCR4, which interacts with CXCL12, a chemokine highly expressed in the BMM and secreted by osteoblasts and BMSCs (**Figure 3**) **(** 159, 160). The expression of CXCR4 is dynamically regulated as the PCs move from the peripheral blood milieu to the BM and significantly decreases upon homing to the BM in response to CXCL12, which is elevated in the BM in contrast to the peripheral blood milieu and its levels are significantly increased in the BM of MM patients (161). CXCL12 induced signaling enhances MM cell motility, facilitates cytoskeletal rearrangements, and promotes transient upregulation and increased affinity of VLA-4 (α4β1) to bind its cognate ligand VCAM1, which is expressed by BM vascular endothelium (161–163). These mechanisms are essential for transendothelial migration of PCs into the BMM and likely play an important role during MM cell recirculation. VLA-4 has also been reported to be crucial for anchoring and retention of MM cells to BM niches (20, 164). Blocking of the CXCL12–CXCR4 interaction disrupts ties between MM cells and the BMM, thereby promoting cell mobilization into the circulation (161, 165). Moreover, in contrast to increased CXCR4 expression, PCs show decreased CXCR5 and CXCR7 expression, leading to loss of responsiveness to B and T zone chemokines CXCL13, CCL9 and CCL21 in the secondary lymphoid structures and lymph nodes (166).

Other crucial molecules involved in PC migration, adhesion of PCs to vascular endothelium and subsequent homing to the BM are the α4β7 integrin, CD44 and E- and P-selectins and their ligands (**Figure 3**) **(** 53, 150). The α4β7 integrin is a receptor that interacts with mucosal vascular addressin cell adhesion molecule 1 (MAdCAM-1), present mainly in venular endothelium, and fibronectin in the BM (167). The contribution of E-selectin during homing of MM to the BM has been shown using enzyme inhibitors (168) and E-selectin blocking antibodies (163). After transendothelial migration, PCs upregulate specific genes with the aim to migrate and adhere to ECM proteins and/or co-localize with other native BM cells within the stromal compartment. The adhesion of MM PCs to the BMM is modulated *via* membrane-embedded tetraspanins, such as CD81 and CD82, which negatively affect myeloma invasion, adhesion, motility, migration as well as secretion of MMP-9 in human MM cell lines (169). In contrast, the transmembrane receptor roundabout guidance receptor 1 (ROBO1) was found to be essential for MM adhesion to BMSCs and ECs and supports homing and dissemination of PCs to the BM niche (170). Furthermore, constitutive activation of cyclin D1 causes increased MM cell adhesion to stromal cells and fibronectin, stabilized F-actin fibers, and also enhanced chemotaxis and inflammatory chemokine secretion (171).

Molecular alterations in the homing signaling of MM PCs lead to dissemination of MM cells outside the BM to the peripheral blood, where they appear as circulating PCs, or their homing to other tissues and/or organs. MM PCs rely on functional CCR1 (ligand: MIP1-α/CCL3) and CCR2 (ligand: MCP-1/CCL2) signaling to regulate PC migration in specific conditions (172). Under hypoxic conditions, HIF2-α strongly induces the expression of CCR1 on the surface of MM PCs. The induced CCR1 signaling abrogates the MM PC homing in response to CXCL12, thereby driving MM cells to egress from the BM to the periphery (173). Likewise, neutralizing or shedding of CD138 increases MM PC motility and rapidly triggers migration of PCs cells *in vivo*, which leads to increased intravasation and dissemination to other bones (174). Moreover, circulating PCs show overexpression of CD44 and CD97 (175). Dysregulation of several factors is implicated in the homing of PCs outside of the BM, where MM cells form extramedullary disease and/or infiltrate solid organs and structures. These include deregulation of the CXCL12–CXCR4 signaling axis (176), upregulated surface expression of CD44, and downregulated CD56 surface expression in extramedullary PCs (177). The presence of circulating MM cells and extramedullary disease is associated with high-risk MM, serves as a poor prognostic factor in MM and is associated with short overall survival (178).

## Metabolomic Reprogramming of MM Within the TME

**Figure 4:**
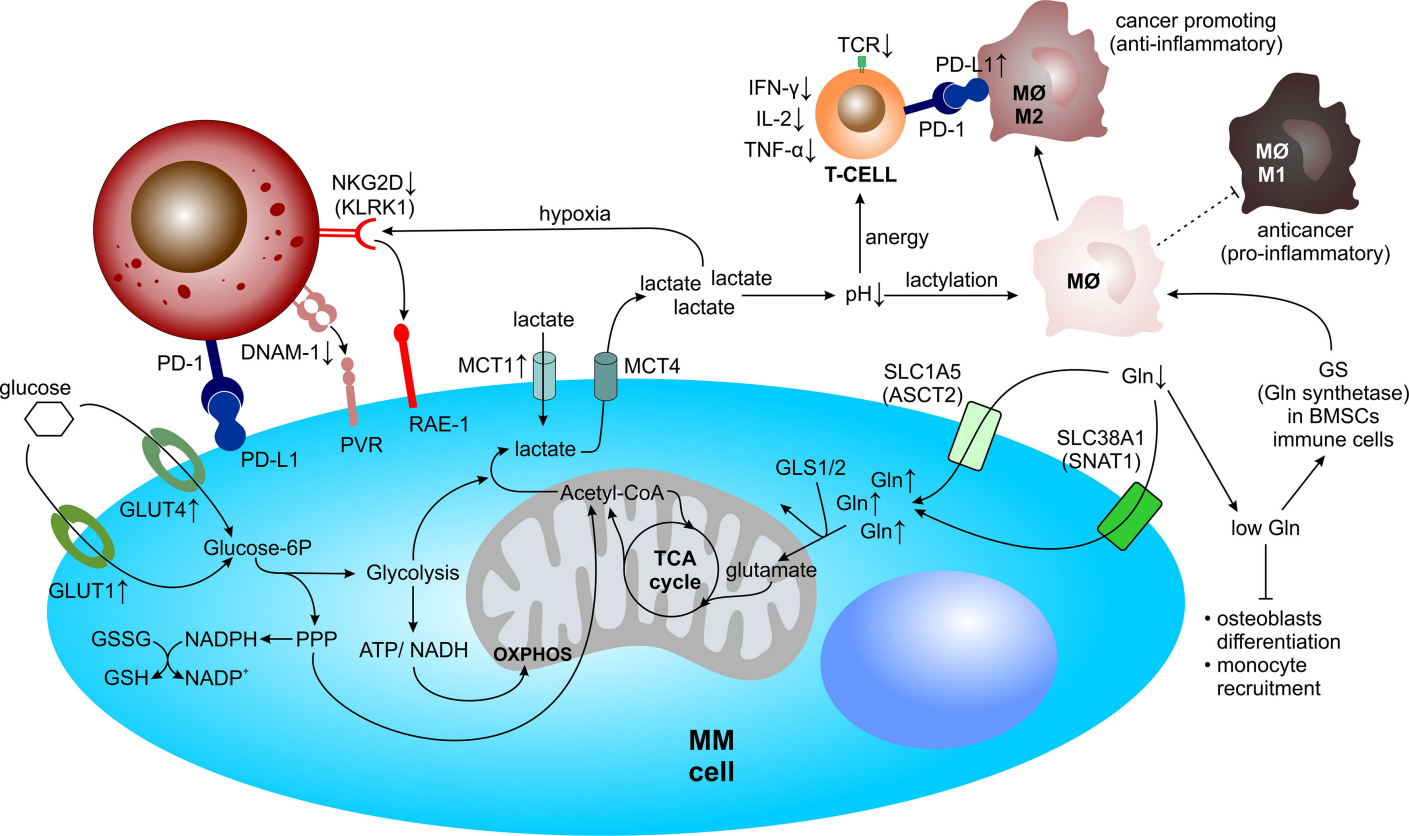
Metabolic reprogramming of MM within the TME. Within the TME, MM cells undergo metabolic adaptations and simultaneously drain their microenvironment of vital resources, such as glucose for glycolysis and/or the pentose phosphate pathway and glutamine for glutaminolysis, allowing for rapid cell proliferation. Concomitantly, metabolic reprogramming shapes the TME towards a hypoxic, acidic, and nutrient depleted milieu, thereby negatively influencing anti-MM immune cell performance. Acetyl-CoA, acetyl-coenzyme A; ATP, adenosine triphosphate; BMSC, bone marrow mesenchymal stromal cell; DNAM-1, DNAX accessory molecule-1; Gln, glutamine; GLS, glutaminase; GLUT, glucose transporter; GS, glutamine synthetase; GSH, glutathione (reduced); GSSG, glutathione (oxidized); IFN-γ, interferon-γ; IL, interleukin; KLRK1, killer cell lectin like receptor K1; MΦ, macrophage; MM, multiple myeloma; MCT, monocarboxylate transporter; NAD(P)+/H, nicotinamide adenine dinucleotide (phosphate) oxidized/reduced; NK, natural killer cell; OXPHOS, oxidative phosphorylation; PD-1, programmed cell death protein 1; PD-L1, programmed cell death 1 ligand 1; PPP, pentose phosphate pathway; PVR, poliovirus receptor; RAE-1, ribonucleic acid export-1; SLC1A5, solute carrier family 1 member 5; SLC38A1, solute carrier family 38 member 1; TCA, tricarboxylic acid; TCR, T cell receptor; TNF-α, tumor necrosis factor-α.

PCs adapt their cellular metabolism to sustain continuous production of the monoclonal immunoglobulins. This is ensured by the enlargement of the ER during PC development, to cope with continuous protein secretion (179). Even in normal PCs, the massive Ig production comes with excessive amounts of misfolded proteins, that are not effectively degraded, thus generating high proteasome load, which may ultimately trigger apoptosis if it reaches a certain threshold (180). Thus, the lifespan of antibody-secreting PCs is tightly regulated and may be rather short. On contrary, MM PCs adapt their protein synthesis and degradation machinery to sustain high proteasome load and eventually evade apoptosis. At the same time, a plethora of anti-apoptotic signals is provided also by the surrounding TME. To survive such high cellular protein turnover and to cope with high energy expenditure and biosynthesis for rapid cell proliferation, MM PCs adapt to their TME by exploiting the available resources (**Figure 4**). Increasing glycolysis and glutaminolysis are two of the most common, but vital prospects for cancer cells in the TME (181, 182). Moreover, the metabolic reprogramming shapes the TME towards a hypoxic, acidic (high lactate levels) and nutrient depleted milieu, thereby supporting cancer proliferation and metastasis (183, 184). However, such an environment negatively impacts anti-tumor immune cell performance (185–187).

### Metabolic Changes in MM PCs Allowing for Their Longevity

#### Aerobic Glycolysis and Lactate Production

In contrast to normal PCs, which switch from glycolysis to oxidative phosphorylation (OXPHOS) during differentiation into antibody-secreting cells (188), MM PCs rely on both increased OXPHOS and glycolysis for survival (189). Aerobic glycolysis is used as a bioenergetic pathway in MM and conversion of glucose into pyruvate provides carbon building blocks for growth, proliferation and protein biosynthesis (190).MM cells display an elevated glycolytic profile and depend on glucose transporters (GLUT), such as GLUT1 and GLUT4 (191, 192). At the same time, enhanced aerobic glycolysis induces the pentose phosphate pathway (PPP) and leads to increased production of reduced nicotinamide adenine dinucleotide phosphate (NADPH) and glutathione (GSH), which both help tumor cells to cope with oxidative stress (193). Lactate, a product of anaerobic glycolysis, together with pyruvate can both serve as major carbon sources to fuel the mitochondrial TCA cycle and thus support OXPHOS as well (194, 195). Lactate and pyruvate are transported *via* bidirectional proton-linked monocarboxylate transporters (MCTs), such as MCT1 (mainly lactate import) and MCT4 (mainly lactate export) (196, 197). MM cells, at least partly, depend on lactate under normoxic conditions and increase the level of MCT1 transporters, whereas they upregulate MCT4 expression under hypoxic conditions to transport lactate outside of the cell, suggesting that in aerobic conditions they use the extracellular lactate as an additional energy source (198).

#### Glutamine Metabolism

Several studies have emphasized the importance of glutamine (Gln) in PC metabolism (199–201). Gln is one of the most abundant free amino acids in the human blood that supports bioenergetics, biosynthesis, tumor growth as well as the production of antioxidants through glutaminolysis (202). During glutaminolysis, Gln is imported into the cell through glutamine transporters such as solute carrier family 1 member 5 (SLC1A5), also known as ASCT2, and solute carrier family 38 member 1 (SLC38A1), also known as SNAT1. Subsequently, it is converted to glutamate *via* glutaminases (GLS1 and GLS2) and further to α-ketoglutarate (α-KG), oxaloacetate and acetyl-CoA, thereby fueling the tricarboxylic acid (TCA) cycle (203–207). At the same time, Gln serves as a major source of nitrogen for synthesis of nonessential amino acids, nucleotides and hexoamines (208). Numerous studies have demonstrated the importance of Gln-derived TCA metabolites in Gln-dependent cancer cells (209–211). Likewise, hematological cancer cells and in particular acute myeloid leukemia (AML) blasts (212, 213) and MM cells (199, 200, 214–216) rely on extracellular Gln supplementation for their growth. A gene expression profiling analysis of multiple datasets revealed increased expression of Gln transporters ASCT2, SNAT1 and solute carrier family 7 member 5 (SLC7A5), also known as LAT1, across the progression of monoclonal gammopathies. Notably, ASCT2 inhibition in human myeloma cell lines (HMCLs) considerably decreased Gln uptake and significantly reduced MM cell growth (199).

Late-stage MM is characterized by strong oncogenic MYC activity (217), which modulates both glycolysis and glutaminolysis (218–221). MYC enhances Gln metabolism by inducing ASCT2 and GLS1 expression to favor glutaminolysis (222, 223). In addition, MM cells mainly depend on extracellular Gln uptake rather than on intracellular Gln synthesis, which is associated with low glutamine synthetase (GS) expression (199). Interestingly, a recent *in vitro* study has shown that Gln depletion in HMCLs induced rapid MYC and Cyclin D1 protein degradation, resulting in increased apoptosis. Moreover, decreased MYC protein levels may potentially have downstream effects which render MM cells more susceptible towards the activity of immune cells, since MYC also regulates anti-tumor immunity through CD47 and PD-L1 *in vivo (* 221). At the same time, Gln withdrawal enhanced the expression and binding of Bcl-2 like protein 11 (BIM) to BCL-2 in MM PCs, sensitizing MM cells towards the BH3-mimetic inhibitor Venetoclax (201).

### Molecular Signaling Pathways and Transcription Factors Involved in Metabolic Reprogramming in MM

Several signaling pathways are involved in metabolic reprogramming of malignant PCs. The PI3K-Akt signaling pathway, which regulates proliferation, growth, survival and other basic cell functions, is upregulated in MM and can be activated by various stimuli, including IL-6 (78) and CXCL12 (161). Once activated, Akt signaling promotes the induction of several glycolytic enzymes, including hexokinase (HK), phosphofructokinase (PFK) and upregulates GLUT1/4 expression (224). In addition, Akt triggers mechanistic target of rapamycin (mTOR)/mTOR complex 1 (mTORC1) activation, leading to enhanced expression of several glycolytic enzymes such as phosphofructokinase 1 (PFK-1) and thus promoting a metabolic shift from physiologically preferred OXPHOS in PCs towards enhancedglycolysis in malignant PCs (225).

AMP-activated protein kinase (AMPK) is a sensor of cellular energy levels. When energy is low, AMPK positively regulates signaling pathways that generate ATP, for example fatty acid﻿ β-oxidation and autophagy, and at the same time inhibits anabolic processes, such as gluconeogenesis, fatty acid synthesis and protein synthesis (226). Moreover, activated AMPK can phosphorylate and activate tuberous sclerosis complex 2 (TSC2), which results in attenuated mTOR signaling, a master regulator of cellular metabolism (227). It has been shown that tumor cells, including MM cells (129), downregulate AMPK to evade its inhibitory effect on cell growth and proliferation (228).

The transcription factors HIF-1α, MYC and P53 also play an important role during metabolic reprogramming of MM cells. HIF-1α is highly expressed in MM BM and is an important regulator of cellular metabolism (229). HIF-1α triggers the expression of glycolytic genes, including GLUT1, HK2, lactate dehydrogenase A (LDHA), pyruvate dehydrogenase kinase (PDK) as well as suppressors of the TCA cycle (230, 231). As mentioned, c-Myc activity is enhanced in MM and is a master regulator of genes involved in glycolysis and glutaminolysis (222). Notably, c-Myc induces transactivation of LDHA (232) and promotes the expression of glucose transporters and major rate-limiting enzymes in glycolysis (233). At the same time, c-Myc regulates cancer cell glutamine metabolism by inducing the expression of ASCT2 and GLS1 (223). The tumor suppressor P53 is mutated in most cancer types, including MM, and its mutational status serves as a robust negative prognostic marker in myeloma (234). P53 suppresses glycolysis and thus favors OXPHOS *via* downregulation of GLUT1/4, and at the same time upregulates phosphatase and tensin homolog (PTEN), a tumor suppressor gene, which inhibits the PI3K-Akt pathway. Therefore, defective P53 pushes ﻿metabolic rewiring of cancer cells towards increased glycolysis (235). In summary, altered activity of the transcription factors HIF-1 α, MYC and P53 results in decreased OXPHOS and simultaneously increased glycolysis and glutaminolysis, which promotes MM cell growth and proliferation by providing them with sufficient amount of carbon building blocks and energy.

### The Impact of Metabolic Changes on the TME and Immunosurveillance in MM

During neoplastic transformation, the malignant PCs rewire their metabolism which enables them to evade apoptotic signals and rapidly proliferate. Concomitantly, cells of the TME adapt their cellular metabolism towards survival in a hypoxic microenvironment with high concentrations of lactic acid and low levels of Gln, forming a MM PCs-supporting milieu (**Figure 4**).

#### Hypoxia

Oxygen-deprived conditions promote immunosuppression and evasion of immune detection. NK cells play an important role in immune monitoring and anti-tumor activity. The surface receptors of NK cells killer cell lectin like receptor K1 (KLRK1), also known as NKG2D, and DNAX accessory molecule-1 (DNAM-1), also known as CD226, are required for cell-mediated killing *via* binding to their ligands ribonucleic acid export-1 (RAE-1) and poliovirus receptor (PVR), respectively, both of which are expressed by MM cells (236, 237). Notably, these receptors are strongly decreased on NK cells derived from MM patients, resulting in impaired NK cell function (42, 238). Several studies reported that hypoxic environments negatively impact NKG2D expression on NK cells, partially due to tumor-derived hypoxic microvesicles (200–1000 nm in diameter) that contain TGF-β (239, 240).

HIF-1α, stabilized during hypoxia with HIF-1β within the HIF-1 complex, directly upregulates PD-L1 expression *via* binding to the hypoxia-response element (HRE) of the PD-L1 gene promoter, thereby contributing to an immunosuppressive TME (241, 242). Notably, PD-L1 expression on MM PCs from minimal residual disease (MRD) positive MM patients is upregulated, in contrast to PCs from healthy donors (243, 244). Subsequently, NK cells derived from MM patients express PD-1 whereas normal NK cells do not (64), suggesting the immunosurveillance to be significantly impaired in patients with positive MRD.

#### Lactate Accumulation

Besides serving as an important source of energy, lactate has an immunomodulatory properties and causes immunosuppression by impairing lymphocyte proliferation, cytokine production and cytotoxic activity (245, 246). Several studies have shown that tumor-cell derived lactate, which lowers the pH within the TME, is able to keep the T lymphocytes in an anergic state. These T cells show reduced cytokine secretion, including IFN-γ, IL-2, and TNF-α, attenuated expression of the T cell receptor (TCR) and IL-2 receptor CD25, as well as impairment of STAT5 and extracellular-signal regulated kinases (ERK) activation after TCR binding (184). Restoring physiological pH levels is able to reverse T cell anergy (247).

Macrophages have great plasticity and exhibit different polarization states dependent on stimulatory effects of their environment. Macrophages can sense the acidity of the TME *via* G protein-coupled receptors (GPCRs), which mediate the expression of inducible cyclic AMP early repressors (ICERs). Subsequently ICERs inhibit the toll-like receptor (TLR)-dependent NFκB signaling, thereby preventing macrophages from polarizing towards a pro-inflammatory and anticancer M1-phenotype (248). Recently, it has been shown that lactate can modulate macrophages *via* epigenetic modification, called lactylation, thereby promoting the polarization of macrophages from the pro-inflamatory and anticancer M1-phenotype to the anti-inflammatory and cancer-promoting M2-phenotype (249). At the same time lactate was shown to induce PD-L1 expression in these M2-like tumor-associated macrophages, which blunts effector T cell function (250).

#### Glutamine Deprivation

Gln dependency of MM PCs and its preferential uptake, rather than the *de novo* synthesis, influences significantly the concentration of Gln in the BM plasma of MM patients. There, concentration of Gln was shown to decrease from 0.6 to 0.4 mM, with a concomitant increase of ammonium as compared with MGUS and SMM patients (199). Such environmental changes may severely affect surrounding cells in the BM. Gln scarcity in the BMM impairs BMSC differentiation into osteoblasts and thus possibly contributes to exacerbated osteolytic bone disease in MM. In addition, Gln deprivation induces changes in the expression of BMSC-derived cytokines and chemokines involved in monocyte recruitment (251) and at the same time it activates the expression of Gln synthase in mesenchymal and immune cells, which leads to M2-like macrophages polarization. Intriguingly, Gln synthase inhibition skews immunosuppressive M2-like macrophages towards pro-inflammatory M1-like macrophages in murine models (252, 253).

## Metabolic Alterations in MM PCs Upon Proteasome Inhibition

Due to their tight balance between protein synthesis and degradation, MM PCs are exceptionally sensitive to PI-induced cytotoxicity (254). Proteasome inhibition has a detrimental effect on protein homeostasis in MM PCs: it, leads to accumulation of the misfolded proteins, and at the same time limits amino acid supply, resulting in cellular starvation (255, 256), which in combination triggers apoptosis. To cope with the harmful effect of acute, proteasome inhibition, MM cells trigger a plethora of molecular mechanisms. These include induction of the UPR and autophagy, formation of inclusion bodies, synthesis of the proteasome subunits to build new proteasomes and a global change in the cellular metabolism (257, 258). These mechanisms generally serve to adapt to proteotoxic stress *via* expansion of the proteosynthetic apparatus, e.g., *via* stabilization of terminal nucleotidyltransferase 5C (TENT5C), also known as FAM46C, a non-canonical poly(A) polymerase, which boosts ER growth in MM and which is tightly regulated *via* proteasomal degradation and autophagy (259).

In the early response to proteasome inhibition, MM PCs increase expression of factors involved in oxidative stress and synthesis of GSH, a major cellular antioxidant. These factors serve as a cellular response to gradually resolve the effects of proteasome inhibition. At later time points and during recovery from proteasome inhibition, surviving MM PCs switch their metabolism from glycolysis to fatty acid oxidation, alter the mitochondrial metabolism and modulate the levels of several amino acids, gradually leading to a decrease in mitochondria metabolism and amino acid supply. In particular the reduced pool of available intracellular amino acids in the cells recovering from proteasome inhibition (e.g., lack of glutamine, arginine, methionine, leucine and lysine) triggers general control nonderepressible 2 (GCN2)-activating transcription factor 4 (ATF4) signaling, which becomes increasingly activated and which further leads to activation of AKT and inhibition of mTORC (260, 261). Thus, the GCN2-ATF4 dependency may represent a novel therapeutic target in MM PCs recovering from PI-induced stress (258).

**Figure 5:**
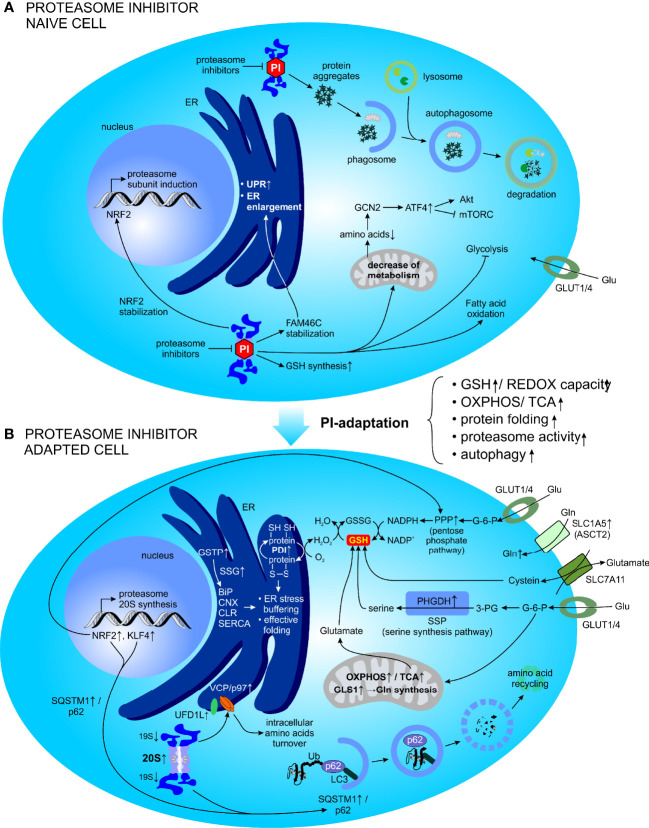
Metabolic factors promoting resistance to PI in MM. **(A)** Adaptive cellular responses following acute and short-term PI treatment. **(B)** Global cellular adaptation towards PI treatment (emerging as the PI resistance). ATF4, activating transcription factor 4; BiP, binding immunoglobulin protein; CLR, calreticulin; CNX, calnexin; ER, endoplasmic reticulum; FAM46C, family with sequence similarity 46 member C; G-6-P, glucose-6-phosphate; GCN2, general control nonderepressible 2; Gln, glutamine; GLS, glutaminase; Glu, glucose; GLUT, glucose transporter; GSH, glutathione (reduced); GSSG, glutathione (oxidized); GSTP, Glutathione S-transferase P; KLF4, kruppel like factor 4; LC3, protein light chain 3; mTORC, mechanistic target of rapamycin complex 1; NAD(P)+/H, nicotinamide adenine dinucleotide (phosphate) oxidized/reduced); NRF2, nuclear factor erythroid 2-related factor 2; OXPHOS, oxidative phosphorylation; PDI, protein disulfide isomerase; 3-PG, 3-phosphoglyceric acid; PHGDH, phosphoglycerate dehydrogenase; PI, proteasome inhibitor; PPP, pentose phosphate pathway; REDOX, reduction–oxidation; SERCA, sarco/endoplasmic reticulum Ca2+-ATPase; SLC1A5, solute carrier family 1 member 5; SLC7A11, solute carrier family 7 member 11; SSG, S-glutathionylation; SSP, serine synthesis pathway; SQSTM1, sequestosome 1; TCA, tricarboxylic acid; Ub, ubiquitin; UFD1L, ubiquitin recognition factor in ER associated degradation; UPR, unfolded protein response; VCP, valosin-containing protein.

In conclusion, to survive proteasome inhibition, MM PCs considerably change their metabolism, mitochondria and the ER to redistribute cellular resources and decrease their fitness (**Figure 5**). Consequently, the mechanisms employed by MM cells to recover from PI-induced stress triggers new and druggable vulnerabilities.

## Metabolic Factors Promoting Resistance to PI in MM

PI resistance remains a major obstacle to the successful treatment of MM. The proteasome is involved in regulation of cellular metabolism and a short-term proteasome inhibition induces a global metabolic response during the recovery phase. Perhaps not surprisingly, adaptive metabolic changes have been shown to promote resistance to PIs (**Figure 5**). Although multiple mechanisms for PIs resistance have been proposed, our mechanistic understanding of the acquisition of PI resistance as well as its potential reversal, remains incomplete. Only recently, the metabolic plasticity of MM PCs that is potentiated by the surrounding TME, was revealed and currently represents a major focus of the study of PI resistance.

### Glycolysis and Lactate Production

Data obtained from cancer cell lines, mouse xenografts and patient-derived tumor samples all indicate a strong association between increased mitochondrial metabolism and decreased PI sensitivity. Notably, when MM cells are forced to use OXPHOS rather than aerobic glycolysis, they develop PI resistance (189, 262). These findings are supported by studies where MM cells increase OXPHOS metabolism when they are forced to survive in the presence of proteasome inhibitors and with subtotal functional inhibition of proteasome activity (263).

On the other hand, during hypoxic conditions, higher glycolytic activity promoted BTZ resistance in MM and subsequent selective inhibition of LDHA sensitized MM cells towards proteasome inhibition *via* by BTZ. At the same time, HIF-1α knockdown decreased lactate levels and partially restored the cytotoxic effect of BTZ, thereby suggesting that LDHA and HIF-1α may be valuable targets in hypoxia-mediated PI resistance (10). Further work reported increased glucose uptake and glycolysis in PI-adapted cells, mainly to fuel the PPP, subsequently leading to increased anti-oxidant capacity that is essential to maintain a PI-resistant phenotype in MM (11).

### Glutamine

The pleiotropic role of Gln in cellular functions has also been reported to promote PI resistance in MM cells by serving as the main fuel source to reinforce mitochondrial respiration and OXPHOS. The same study has demonstrated that targeting Gln-induced respiration in PI resistant cells, using the GLS-1 inhibitor CB-839, synergized well with PIs, such as CFZ, to induce severe ER stress and apoptotic cell death in MM cells (214). More recently, the expression of ASCT2, a Gln transporter otherwise important for MM PCs survival (199), was studied with respect to PI resistance. PI-resistant MM depends on ASCT2 and Gln uptake as well and the combination of ASCT2 inhibitors (ASCT2i) synergistically potentiated the cytotoxicity of PIs in MM *via* induction of apoptosis and modulation of autophagy. Moreover, RNA sequencing of HMCLs treated with CFZ and ASCTi revealed that the drug combination strongly reduced Gln metabolism regulators, including MYC and NRAS and likewise upregulation of Gln metabolism was associated with advanced disease stage and PI resistance in patients with RRMM (264). Thus, therapeutic approaches to specifically disrupt Gln metabolism may be an effective strategy to interfere with cancer metabolism and tumor progression in combination therapy (199, 214, 265).

### Glutathione

Glutathione (GSH),a tripeptide, is one of the most abundant and effective tools that cells can exploit in detoxification of drugs and xenobiotics in general. As a potent reductant, it can react with oxidizing agents before they interact with critical cellular constituents, such as nucleic acids, proteins, or lipids. Moreover, it is involved in a plethora of antioxidant reactions, where it serves as a cofactor and thus may indirectly modulate cell proliferation, apoptosis, immune function and fibrogenesis (266–270). GSH exists in the thiol-reduced form, which accounts for >98% of total GSH, disulfide-oxidized (GSSG) form or in the glutathione conjugated form (GS-R) (267, 271). Cellular GSH is in the cytosol (80-85%), mitochondria (10-15%) and a small fraction is present in the ER (272–274). A high GSH to GSSG ratio indicates a good intracellular redox potential, which represents the ability of a cell to cope with oxidative stress (268). Aerobic metabolism produces vast amounts of ROS, which can be metabolized by GSH peroxidase (GPx), thus reducing ROS, oxidizing GSH, and generating GSSG. In turn, GSSG can be reduced back to GSH by GSSG reductase (GR) at the expense of NADPH, thereby completing the redox cycle (275). In addition, GSH is able to form disulfide bonds with cysteine residues of proteins or to protect thiols under oxidative stress in a process called *via* S-glutathionylation (SSG), a redox-regulated post-translational thiol modification involved in regulation of protein function (276).

GSH is a major determinant of BTZ cytotoxicity in MM and likewise proteasome inhibition induces GSH synthesis, thereby providing the cell with a more robust intracellular buffering system to protect against oxidative stress while simultaneously decreasing the amount of misfolded proteins that need to be ubiquitinated and subsequently cleared by the ubiquitin-proteasome system (277, 278). Therefore a plethora of mechanisms of adaptation to PI focus on increased GSH/GSSG ratio. A recent study has demonstrated that PI resistant MM cells exhibit increased expression of Glutathione S-transferase P (GSTP), which mediates SSG of ER resident proteins, such as binding immunoglobulin protein (BiP), calnexin, calreticulin, endoplasmin, sarco/endoplasmic reticulum Ca^2+^-ATPase (SERCA), and other protein-folding and redox-active proteins, thereby regulating their activities (279–281). Preventing S-glutathionylation in MM cells using a GSTP specific inhibitor restored BiP chaperon activity and/or ATPase activities and reversed resistance towards BTZ (279). Additionally, it was shown that BTZ-induced cytotoxicity was strongly reduced in HMCLs, such as ANBL-6 and INA-6, that were supplemented with cysteine or its derivative GSH. Further mechanistic studies revealed that increased intracellular GSH levels impaired the BTZ-induced nuclear factor erythroid 2-related factor 2 (NRF2)-associated stress response primarily *via* upregulation of the xCT subunit of the cystine/glutamate antiporter (SLC7A11). The inhibition of the x_c_ $x_{\text{c}}^{-}$ activity increased the BTZ-induced cytotoxicity in a subset of HMCLs and primary MM cells, and re-established BTZ sensitivity in BTZ adapted cells (277). Another study has shown that docosahexaenoic acid (DHA) and eicosapentaenoic acid (EPA) supplementation of MM cells, prior the treatment with BTZ, potently decreased cellular GSH levels and altered the expression of related metabolites and key enzymes involved in GSH metabolism, suggesting a critical role of GSH degradation in overcoming BTZ resistance in MM. In addition, the NRF2–ATF3/AFT4–ChaC glutathione specific gamma-glutamylcyclotransferase 1 (CHAC1) signaling pathway was shown to be potentially implicated in DHA/EPA pretreatment-mediated GSH degradation (282).

GSH can be produced from glutamate, cysteine and glycine. In addition, cysteine can be intracellularly converted from serine for GSH synthesis. The serine synthesis pathway (SSP) has been recently demonstrated to be associated with increased BTZ resistance in MM (11). Phosphoglycerate dehydrogenase (PHGDH), the first rate-limiting enzyme in the SSP, was reported to be significantly elevated in CD138+ PCs derived from patients with relapsed MM. In addition, MM cells overexpressing PHGDH exhibited increased cell growth, tumor formation, and resistance to BTZ *in vitro* and *in vivo*, whereas inhibition of PHGDH caused decreased cell growth and BTZ resistance in MM cells. Lastly, PHGDH reduced ROS levels *via* increased GSH synthesis, thereby promoting cell growth and likewise BTZ resistance in MM cells (283). Similarly, glycine has been found to promote MM cell proliferation *in vitro* and *in vivo*, and GSH synthesis was identified as the main metabolic pathway contributing to proliferation of MM cells. Thus, pharmacological blockage of glycine uptake and utilization for GSH synthesis shows therapeutic potential in MM treatment (284).

In summary, GSH has central role in MM biology and is the major factor protecting MM PCs from BTZ-induced cytotoxicity. It does not directly interfere with BTZ-induced proteasome inhibition (277), but impairs its cytotoxicity mainly *via* anti-oxidant defense, protein modification and reduction of proteotoxic stress.

### Specific Metabolic Alterations of PI Resistant MM

Beyond the major metabolic routes of adaptation described above, such as high GSH/GSSG ratio, PI-resistant cells show a global metabolic shift to sustain high protein production and proliferation, but at the same time to survive the consequences of proteasome inhibition (**Figure 5**). Proteomic analysis of PI-adapted MM cells revealed elevated levels of proteins involved in metabolic regulation, protein catabolism, fatty acid/β-oxidation, redox control, protein folding and glutathione regulation, whereas downregulated pathways comprised DNA transcription/protein translation, differentiation, lipid biosynthesis, apoptosis, and structural/cytoskeletal functions compared to non-adapted control cells. The same study has further shown increased OXPHOS in PI resistant MM. The *in vitro* model of MM cell lines adapted to culture conditions containing lethal concentrations of BTZ or CFZ (so-called PI-adapted MM cells) display overexpression of several glycolytic enzymes, NADPH dehydrogenase and NAD(P)H generating enzymes, such as enzymes of the oxidative branch of the PPP and malate dehydrogenase, suggesting that enhanced production of reducing equivalents as adaptive metabolic responses of MM cells to sustain proteasome inhibition (12). Importantly, these reducing equivalents are crucial for the maintenance of redox balance and neutralization of ROS in the mitochondria, and at the same time are important for effective protein folding in the ER. Interestingly, PI-adapted cells also show increased mitochondrial activity compared to PI-naïve cells, indicating that enhanced glycolytic flux may play a role in fueling the PPP and TCA to, among other things, support the production of reducing equivalents to buffer deleterious effects of proteasome inhibition. In line with that, PI-adapted cells display increased expression levels of protein disulfide isomerase (PDI), a key enzyme that oxidizes reduced thiols (-SH) of cysteine residues in nascent proteins, thereby catalyzing disulfide bond formation (S-S) and show more effective protein folding in the ER (285, 286), compared to control cells not adapted to PI containing growth conditions (12). Thus, PI adapted MM cells commonly exhibit lower dependency on proteasome activity, partly owing to an improved protein folding machinery which allows them to alleviate PI-induced proteotoxic stress (12). Further mass spectrometry-based, whole metabolomic profiling combined with metabolite pathway and metabolite set enrichment analysis confirmed the proteomic findings and validated the importance of enhanced antioxidant capacity higher redox homeostasis, and NAD(P)^+^/NAD(P)H levels, all of which ultimately lead to improved protein folding and thus less proteotoxic stress in PI resistant MM cells (263). Subsequently, targeting protein folding as a strategy for the treatment of MM has been proposed and strategies to inhibit PDI to overcome PI resistance were shown to effectively and selectively induce cytotoxicity in MM *in vitro* and *in vivo (* 287, 288).

An alternative proteolytic pathway to replenish the exhausted amino acid pool, which is also involved in PI resistance is autophagy (289–293), a process that regulates sequestration of cellular components, such as misfolded cytoplasmic proteins, protein aggregates (aggresomes) or damaged organelles, into a double-membrane vesicles (autophagosomes). These vesicles subsequently fuse with a lysosome (autolysosome), which results in the degradation of its contents by lysosomal hydrolases, thereby replenishing the cells amino acid pool (294). Thus, autophagy-dependent nutrient recycling may be one way for MM cells to alleviate PI-induced proteotoxic stress and cope with amino acid depletion, thereby increasing PI resistance (255, 256). Sequestosome 1 (SQSTM1 or p62) is the autophagy-associated cargo receptor that bridges ubiquitinylated proteins and autophagosomes and serves as a critical mediator of autophagy and PI-resistance (292, 293). In CFZ resistant HMCLs, SQSTM1 is overexpressed *via* the activation of NRF2 which enhances fatty acid/β-oxidation and in turn increased intracellular NADPH levels (293) and at the same time *via* kruppel like factor (KLF4) which participates in autophagy pathways activated during stress responses (295).

Additional mechanisms that contribute to increased PI resistance of MM cells involve the upregulation of the 20S proteasome subunits (296, 297) and, paradoxically, the downregulation of 19S proteasome subunits (298). The latter findings have been confirmed by showing that modest reduction of the expression of individual subunits of the 19S proteasome complex, such as PSMC6 increased MM survival and protected cells from the PI-induced cytotoxicity (299). Underlying mechanisms included an increased ratio of the 20S to 26S proteasomes, preservation of protein degradation capacity and reduced proteotoxic stress (300). Related work from thousands of cancer cell lines and tumors indicate that suppressed expression of one or more 19S proteasome subunits led to intrinsic PI resistance and is associated with poor outcome in MM patients (301). Of note, depletion of the 19S proteasome subunits leads to increase in SQSTM1 and proteins maintaining protein homeostasis and involved in the ERAD: ubiquitin recognition factor in ER associated degradation (UFD1L) and the triple AAA ATPase valosin-containing protein (VCP)/p97 (298, 299). At the same time, VCP/p97 has been suggested to be a regulator of cellular metabolism, as glutamine depletion leads to increased VCP/p97 expression, whereas VCP/p97 inhibition perturbs metabolic processes and intracellular amino acid turnover (302). Subsequently, pharmacological depletion of VCP/p97 activity with different inhibitors or with approved drug Disulfiram induced proteotoxic stress and cytotoxicity in MM, including PI resistant MM (256, 303).

In conclusion, adaptation to proteasome inhibition in MM PCs involves a broad network of changes in the ER and mitochondria, which allows for reduced oxidative and proteotoxic stress in the cells.

## TME-Mediated PI Resistance

**Figure 6:**
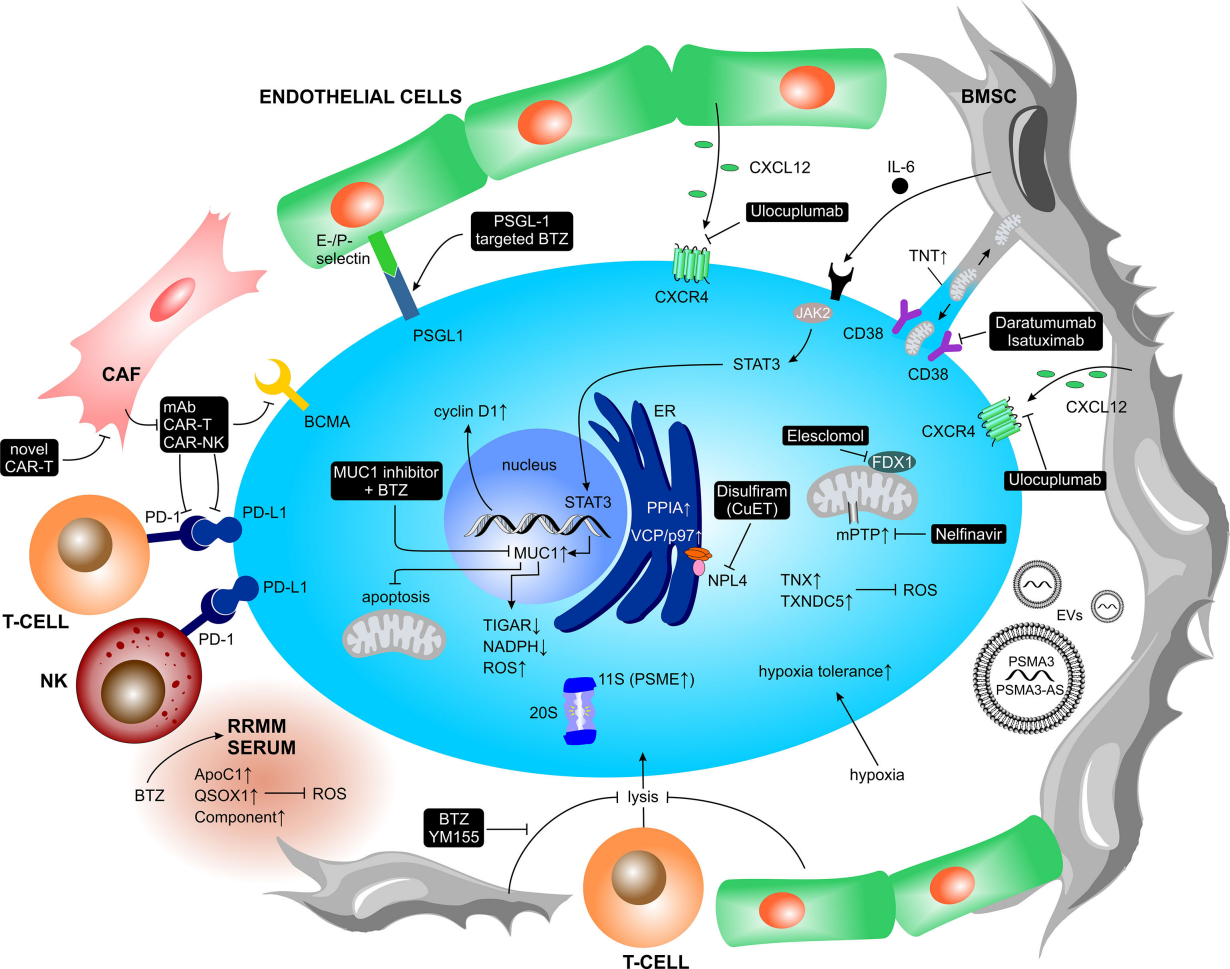
TME-mediated PI resistance and therapeutic strategies to overcome TME-mediated PI resistance. The complex contact-dependent and contact-independent interactions of myeloma PCs with the surrounding tumor microenvironment mediate MM adaptation to PI treatment. Black boxes illustrate potential treatment strategies to overcome TME-mediated PI resistance in MM. ApoC1, apolipoprotein C1; BCMA, B-cell maturation antigen; BMSC, bone marrow stromal cell; BTZ, Bortezomib; CAF, cancer-associated fibroblast; CAR-T, chimeric antigen receptor T-cell; CAR-NK, chimeric antigen receptor natural killer cell; CXCL, C-X-C motif chemokine ligand; CXCR, C-X-C motif chemokine receptor; EC, endothelial cell; ER, endoplasmic reticulum; EV, extracellular vesicle; FDX1, ferredoxin 1; IL-6, interleukin-6; JAK2, janus kinase 2; mAB, monoclonal antibody; mPTP, mitochondrial permeability transition pore; MUC1, mucin 1; NADP(H), nicotinamide adenine dinucleotide phosphate (reduced); NK, natural killer cell; NPL4, nuclear protein localization 4 homolog; PD-1, programmed cell death protein 1; PD-L1, programmed cell death 1 ligand 1; PPIA, peptidylprolyl isomerase A; PSGL-1, P-selectin glycoprotein ligand-1; PSMA3, proteasome 20S subunit alpha 3; PSMA-AS1, PSMA3 antisense RNA 1; PSME, proteasome activator subunit; QSOX1, quiescin sulfhydryl oxidase 1; ROS, reactive oxygen species; STAT3, signal transducer and activator of transcription 3; TIGAR, p53-inducible regulator of glycolysis and apoptosis; TNT, tunneling nanotubes; TNX, thioredoxin; TXNDC5, thioredoxin domain-containing protein 5.

The metabolic changes previously described highlight PI-induced adaptations in resistant MM cells that alleviate cellular and proteotoxic stress. However, adaptation to PI is not exclusively cell intrinsic, but is supported and potentially even driven by the surrounding TME as well (**Figure 6**).

MM cells co-cultured with stromal cells show decreased apoptosis following exposure to alkylating agents as well as the PI BTZ. Upon co-culture, stromal cell-derived IL-6 induces JAK2/STAT3 signaling in MM cells, thereby increasing the expression of the MUC1 oncogene, a gene known to confer resistance to apoptotic cell death. In addition, silencing of MUC1 *via* MUC1-specific shRNA partially reversed stromal cell induced resistance towards BTZ (304). Related work has shown that inhibition of the C-terminal transmembrane domain of MUC1 (MUC1-C) is synergistic with BTZ in downregulating p53-inducible regulator of glycolysis and apoptosis (TIGAR) expression, as well as in depleting NADPH and increasing ROS levels, leading to MM cell death (305). Moreover, BTZ supplementation to the co-culture of BMSCs and primary MM cells or HMCLs increased mitochondrial trafficking, thereby reinforcing bioenergetic plasticity in MM cells (75). Importantly, resistance to CFZ has been shown to be mediated by the co-culture with BMSCs in normal and in cyclin D1 overexpressing MM PCs. However, since cyclin D1 expression enhances MM cell adhesion to stromal cells and fibronectin, favors cell migration, and increases chemotaxis as well as inflammatory chemokine secretion, CFZ-mediated resistance was alleviated particularly in cyclin D1 expressing cells by the immunomodulatory drugs, which modify MM–TME interactions, such as CAM-DR (171).

A very recent study has identified PSMA3 and PSMA3-AS1 as BMSCs-derived exosomal RNAs that are released within the BMM and are subsequently absorbed by MM cells, leading to increased PI resistance. PSMA3-AS1 can form an RNA duplex with pre-PSMA3, which promotes PSMA3 expression by reinforcing its stability. In xenograft models, intravenously administered siPSMA-AS1 was found to enhance CFZ sensitivity. Moreover, elevated levels of plasma circulating exosomal PSMA3 and PSMA3-AS1 derived from MM patients were strongly associated with decreased progression-free survival (PFS) and poor overall survival (OS), suggesting a prognostic value in clinical settings (306).

Single cell RNA-sequencing (scRNA-seq) data from primary BTZ-refractory and early relapsed patients revealed hypoxia tolerance, protein folding, and mitochondrial respiration as molecular pathways involved in PI resistance in patients. Those findings have been validated in larger clinical cohorts such as the MMRF CoMMpass Study. In addition, the study has found peptidylprolyl isomerase A (PPIA), a crucial enzyme in the protein folding response pathway, as a potential new therapeutic target to overcome PI resistance. Indeed, inhibition of PPIA using the small molecule inhibitor Ciclosporin or PPIA gene deletion *via* CRISPR-Cas9 gene editing significantly sensitized MM cells towards PIs (13). At the same time, comparative proteomic profiling of PCs from MM patients responding to BTZ-containing regimens versus non-responders has shown that increased levels of proteasome activator complex subunit 1 (PSME1) and anti-oxidative proteins, such as thioredoxin (TXN) and thioredoxin domain-containing protein 5 (TXNDC5) play a major role in BTZ resistance in patients (307). Moreover, analysis of BTZ-induced systemic changes *via* proteomic profiling of sera from RRMM patients revealed elevated levels of apolipoprotein C1 (ApoC1), quiescin sulfhydryl oxidase 1 (QSOX1) and complement components in MM patients with lower response rates towards BTZ-containing regimens. Notably, QSOX1 contains domains of thioredoxin, confirming previous findings of thioredoxins’ role in ROS homeostasis and BTZ-therapy resistance (308).

In summary, data from co-culture experiments and MM patients not responding to PI-based therapy confirm alleviated oxidative and proteotoxic stress in PCs to be among the most efficient strategies to withstand PI treatment. These can be achieved *via* intrinsic changes in PCs and/or can be promoted by the surrounding TME.

## Overcoming TME-Mediated PI Resistance

Advancing our understanding of multi-drug resistance in relapsed and late-stage MM patients is critical for the development of more effective therapeutic strategies, which consider not only intracellular metabolic alterations in MM cells but also appraise interactions of MM cells with other members of the TME (**Figure 6**).

Current clinical practice uses monoclonal anti-CD38 antibodies, such as Daratumumab (309, 310) or Isatuximab (311, 312), in combination with a PI and IMiDs as standard upfront therapy (313). Given the fact that PI-induced mitochondrial trafficking between BMSCs and MM PCs is a CD38-dependent process (75), this provides a solid biological rational for combining PIs in triplet/quadruplet therapies as per current practice.

Since PI-resistant MM cells exhibit enhanced mitochondrial metabolism and increased ROS buffering to sustain more effective protein folding, this characteristic represents a specific vulnerability that can be targeted therapeutically. Elesclomol may exploit this unique metabolic characteristic of PI-resistant MM cells by further increasing oxidative stress levels beyond the critical threshold and induce MM cell death. Indeed, cancer cells with increased mitochondrial energy metabolism exhibit increased sensitivity to Elesclomol due to impaired ability to cope with oxidative stress (314). Elesclomol directly targets the mitochondrial reductase ferredoxin 1 (FDX1), which has been identified as the primary mediator of Elesclomol-induced toxicity, leading to a unique form of copper-dependent cell death (262, 314). Likewise, Nelfinavir, an HIV-protease inhibitor, has been shown to impair glucose uptake and oxidative metabolism in MM and PI resistant MM due to modulation of lipid bilayer fluidity in mitochondria and therefore affecting the proper function of the mitochondrial transition pore (mPTP) (315). These effects of Nelfinavir together with proteasome inhibition provided by BTZ showed high efficacy and high response rate in heavily pretreated RRMM in Phase II studies (316, 317). In a similar fashion to repurpose Nelfinavir for the treatment of RRMM, the Disulfiram-based compound CuET is able to overcome the PI resistance in MM and may therefore represent a promising and readily available treatment option for RRMM patients (318). Moreover, modulation of the PI-adaptive metabolism with Metformin or with a GCN-specific inhibitor after PI exposure in a PI-recovery state (7 days after the treatment) may overcome the adaptation to the treatment with PI over time (258).

One of the novel approaches to overcome PI resistance in the context of TME is targeted delivery of the PI drugs within the BMM. A new concept of drug delivery used targeted nanoparticles, which improve specificity and efficacy while at the same time reducing the toxicity of therapeutic drugs. Targeting the MM cells within their BMM *via* PSGL-1-targeted BTZ, and Rho-associated, coiled-coil containing protein kinase (ROCK) inhibitor-loaded liposomes is more effective than free drugs, non-targeted liposomes, and single-agent controls, and reduces BTZ-induced side effects. These results support the use of PSGL-1-targeted liposomal BTZ-containing formulations to overcome drug resistance and improve patient outcomes in MM (319). Several recent review papers summarize the plethora of available immunotherapies and their mechanism of action in MM (checkpoint inhibitors, or T-cell engaging bispecific antibodies and/or adoptive T-cell/NK cell therapy) (320–322), therefore we will not cover this topic in the present review. Targeting the interaction of malignant PCs with the TME may weaken the TME-mediated drug resistance and/or decrease the immunosuppressive activity and environment induced by active MM. Therapeutic targeting of the CXCL12–CXCR4 axis using CXCR4 inhibitors weakens the adhesion of MM PCs on the TME, which renders them more sensitive to PI treatment (165). Likewise, blockade of the CXCL12–CXCR4 axis *via* monoclonal antibodies like Ulocuplumab shows promising signals for activity in combination with BTZ (323). Further, BMSCs and vascular ECs from MM patients and healthy individuals significantly inhibit the T cell-mediated MM cell lysis in a cell–cell contact dependent manner and without substantial T cell suppression, suggesting the induction of a cell adhesion-mediated immune resistance (CAM-IR) against cytotoxic T cell lymphocytes. However, BTZ increased the cytotoxic T cell lymphocyte-mediated lysis of MM cells. In addition, repression of survivin using the small molecule inhibitor YM155 synergized with cytotoxic T cell lymphocytes and abrogated CAM-IR *in vitro* and *in vivo (* 324). Recently, cancer-associated fibroblasts were shown to inhibit CAR T-cell anti-MM activity and to promote MM progression. At the same time, novel CAR T-cells targeting both MM cells and cancer-associated fibroblasts significantly improved the effector functions of CAR T-cells in MM and represent novel strategy to overcome CAR T-cell therapy resistance (325).

Such studies emphasize the importance of understanding TME-dependent and cell intrinsic metabolic adaptations in PI-resistant MM PCs, to tailor novel feasible therapies to overcome PI-resistance in MM patients.

## Conclusion

In conclusion, PI resistance is a multifaceted phenomenon that emerges upon long term and sublethal drug exposure, which allows for a wide metabolic adaptation in MM PCs subclones over time. Emerging evidence shows that beyond cell intrinsic changes, malignant PCs shape and fine-tune the BMM according to their needs to exploit resources that help them to cope with high energy demands and high redox capacity required for alleviation of oxidative and proteotoxic stress ultimately leading to increased survival in the presence of PI treatment. At the same time, therapy persistent MM PCs shape the BMM to an immunosuppressive state that supports the escape from the immune surveillance.

Recent studies have shown multiple ways to target RRMM, either by targeting MM PCs directly, by targeting the microenvironment, or by strengthening the immune response. The rise of IMiDs and targeted mABs treatments indicate our growing understanding of the therapeutic role of targeting the microenvironment. Overall, advancing our understanding of multi-drug resistance is critical for the development of more effective strategies for the treatment of RRMM.

## Author Contributions

JS drafted and conceptualized the manuscript. AB proofread the manuscript, conceptualized, and prepared the figures. CD provided critical revision of the manuscript. LB conceptualized the manuscript and the figures, supervised the writing, and finalized the manuscript. All authors have read and agreed to the published version of the manuscript.

## Funding

The work was supported by the Krebsliga Schweiz grant KFS-4990-02-2020.

## Conflict of Interest

The authors declare that the research was conducted in the absence of any commercial or financial relationships that could be construed as a potential conflict of interest.

## Publisher’s Note

All claims expressed in this article are solely those of the authors and do not necessarily represent those of their affiliated organizations, or those of the publisher, the editors and the reviewers. Any product that may be evaluated in this article, or claim that may be made by its manufacturer, is not guaranteed or endorsed by the publisher.

## References

[1] Kyle RA RS. Multiple Myleoma. Blood (2008) 111:2962–72. doi: 10.1182/blood-2007-10-078022 PMC2265446 18332230

[2] Keats JJ Chesi M Egan JB Garbitt VM Palmer SE Braggio E. Clonal Competition With Alternating Dominance in Multiple Myeloma. Blood (2012) 120(5):1067–76. doi: 10.1182/blood-2012-01-405985 PMC3412330 22498740

[3] Bolli N Avet-Loiseau H Wedge DC Van Loo P Alexandrov LB Martincorena I. Heterogeneity of Genomic Evolution and Mutational Profiles in Multiple Myeloma. Nat Commun (2014) 5:2997. doi: 10.1038/ncomms3997 24429703 PMC3905727

[4] Gandhi UH Cornell RF Lakshman A Gahvari ZJ McGehee E Jagosky MH. Outcomes of Patients With Multiple Myeloma Refractory to CD38-Targeted Monoclonal Antibody Therapy. Leukemia (2019) 33(9):2266–75. doi: 10.1038/s41375-019-0435-7 PMC6820050 30858549

[5] Lecker SH Goldberg AL Mitch WE. Protein Degradation by the Ubiquitin-Proteasome Pathway in Normal and Disease States. J Am Soc Nephrol (2006) 17(7):1807–19. doi: 10.1681/ASN.2006010083 16738015

[6] Kubiczkova L Pour L Sedlarikova L Hajek R Sevcikova S. Proteasome Inhibitors - Molecular Basis and Current Perspectives in Multiple Myeloma. J Cell Mol Med (2014) 18(6):947–61. doi: 10.1111/jcmm.12279 PMC4508135 24712303

[7] Besse A Besse L Kraus M Mendez-Lopez M Bader J Xin BT. Proteasome Inhibition in Multiple Myeloma: Head-to-Head Comparison of Currently Available Proteasome Inhibitors. Cell Chem Biol (2019) 26(3):340–51.e3. doi: 10.1016/j.chembiol.2018.11.007 30612952

[8] Lee AH Iwakoshi NN Anderson KC Glimcher LH. Proteasome Inhibitors Disrupt the Unfolded Protein Response in Myeloma Cells. Proc Natl Acad Sci USA (2003) 100(17):9946–51. doi: 10.1073/pnas.1334037100 PMC187896 12902539

[9] Obeng EA Carlson LM Gutman DM Harrington WJ Lee KP Boise LH. Proteasome Inhibitors Induce a Terminal Unfolded Protein Response in Multiple Myeloma Cells. Blood (2006) 107(12):4907–16. doi: 10.1182/blood-2005-08-3531 PMC1895817 16507771

[10] Maiso P Huynh D Moschetta M Sacco A Aljawai Y Mishima Y. Metabolic Signature Identifies Novel Targets for Drug Resistance in Multiple Myeloma. Cancer Res (2015) 75(10):2071–82. doi: 10.1158/0008-5472.CAN-14-3400 PMC4433568 25769724

[11] Zaal EA Wu W Jansen G Zweegman S Cloos J Berkers CR. Bortezomib Resistance in Multiple Myeloma Is Associated With Increased Serine Synthesis. Cancer Metab (2017) 5:7. doi: 10.1186/s40170-017-0169-9 28855983 PMC5575874

[12] Soriano GP Besse L Li N Kraus M Besse A Meeuwenoord N. Proteasome Inhibitor-Adapted Myeloma Cells Are Largely Independent From Proteasome Activity and Show Complex Proteomic Changes, in Particular in Redox and Energy Metabolism. Leukemia (2016) 30(11):2198–207. doi: 10.1038/leu.2016.102 PMC5097071 27118406

[13] Cohen YC Zada M Wang SY Bornstein C David E Moshe A. Identification of Resistance Pathways and Therapeutic Targets in Relapsed Multiple Myeloma Patients Through Single-Cell Sequencing. Nat Med (2021) 27(3):491–503. doi: 10.1038/s41591-021-01232-w 33619369 PMC7612793

[14] Bianchi G Munshi NC. Pathogenesis Beyond the Cancer Clone(s) in Multiple Myeloma. Blood (2015) 125(20):3049–58. doi: 10.1182/blood-2014-11-568881 PMC4432002 25838343

[15] Child JA Morgan GJ Davies FE Owen RG Bell SE Hawkins K. High-Dose Chemotherapy With Hematopoietic Stem-Cell Rescue for Multiple Myeloma. N Engl J Med (2003) 348(19):1875–83. doi: 10.1056/NEJMoa022340 12736280

[16] Kint N Vlayen S Delforge M. The Treatment of Multiple Myeloma in an Era of Precision Medicine. Expert Rev Precis Med Drug Dev (2019) 4(3):153–62. doi: 10.1080/23808993.2019.1606672

[17] Nooka AK Kastritis E Dimopoulos MA Lonial S. Treatment Options for Relapsed and Refractory Multiple Myeloma. Blood (2015) 125(20):3085–99. doi: 10.1182/blood-2014-11-568923 25838342

[18] Gao Q Wang L Wang S Huang B Jing Y Su J. Bone Marrow Mesenchymal Stromal Cells: Identification, Classification, and Differentiation. Front Cell Dev Biol (2022) 9:787118. doi: 10.3389/fcell.2021.787118 35047499 PMC8762234

[19] Liang X Song E. The Role of Bone Marrow Stromal Cells in Blood Diseases and Clinical Significance as a Crucial Part of the Hematopoietic Microenvironment. Ann Blood. (2020) 5:2–2. doi: 10.21037/aob.2019.12.03

[20] Michigami T Shimizu N Williams PJ Niewolna M Dallas SL Mundy GR. Cell–Cell Contact Between Marrow Stromal Cells and Myeloma Cells *via* VCAM-1 and α4β1-Integrin Enhances Production of Osteoclast-Stimulating Activity. Blood (2000) 96(5):1953–60. doi: 10.1182/blood.V96.5.1953 10961900

[21] Abe M Hiura K Wilde J Moriyama K Hashimoto T Ozaki S. Role for Macrophage Inflammatory Protein (MIP)-1α and MIP-1β in the Development of Osteolytic Lesions in Multiple Myeloma. Blood (2002) 100(6):2195–202. doi: 10.1182/blood.V100.6.2195 12200385

[22] Terpos E Dimopoulos MA. Myeloma Bone Disease: Pathophysiology and Management. Ann Oncol (2005) 16(8):1223–31. doi: 10.1093/annonc/mdi235 15928069

[23] Croucher PI Shipman CM Lippitt J Perry M Asosingh K Hijzen A. Osteoprotegerin Inhibits the Development of Osteolytic Bone Disease in Multiple Myeloma. Blood (2001) 98(13):3534–40. doi: 10.1182/blood.V98.13.3534 11739154

[24] Standal T Seidel C Hjertner O Plesner T Sanderson RD Waage A. Osteoprotegerin Is Bound, Internalized, and Degraded by Multiple Myeloma Cells. Blood (2002) 100(8):3002–7. doi: 10.1182/blood-2002-04-1190 12351414

[25] Giuliani N Colla S Morandi F Lazzaretti M Sala R Bonomini S. Myeloma Cells Block RUNX2/CBFA1 Activity in Human Bone Marrow Osteoblast Progenitors and Inhibit Osteoblast Formation and Differentiation. Blood (2005) 106(7):2472–83. doi: 10.1182/blood-2004-12-4986 15933061

[26] Oshima T Abe M Asano J Hara T Kitazoe K Sekimoto E. Myeloma Cells Suppress Bone Formation by Secreting a Soluble Wnt Inhibitor, sFRP-2. Blood (2005) 106(9):3160–5. doi: 10.1182/blood-2004-12-4940 16030194

[27] Vacca A Ribatti D Roncali L Ranieri G Serio G Silvestris F. Bone Marrow Angiogenesis and Progression in Multiple Myeloma. Br J Haematol (1994) 87:503–8. doi: 10.1111/j.1365-2141.1994.tb08304.x 7527645

[28] Rajkumar SV Mesa RA Fonseca R Schroeder G Plevak MF Dispenzieri A. Bone Marrow Angiogenesis in 400 Patients With Monoclonal Gammopathy of Undetermined Significance, Multiple Myeloma, and Primary Amyloidosis. Clin Cancer Res (2002) 8(7):2210–6. 12114422

[29] Carmeliet P Jain RK. Angiogenesis in Cancer and Other Diseases. Nature (2000) 407(6801):249–57. doi: 10.1038/35025220 11001068

[30] Bhaskar A Tiwary BN. Hypoxia Inducible Factor-1 Alpha and Multiple Myeloma. Int J Adv Res (2016) 4(1):706–15. PMC4760640 26900575

[31] Vande Broek I Asosingh K Allegaert V Leleu X Facon T Vanderkerken K. Bone Marrow Endothelial Cells Increase the Invasiveness of Human Multiple Myeloma Cells Through Upregulation of MMP-9: Evidence for a Role of Hepatocyte Growth Factor. Leukemia (2004) 18(5):976–82. doi: 10.1038/sj.leu.2403331 14999296

[32] Purushothaman A Uyama T Kobayashi F Yamada S Sugahara K Rapraeger AC. Heparanase-Enhanced Shedding of Syndecan-1 by Myeloma Cells Promotes Endothelial Invasion and Angiogenesis. Blood (2010) 115(12):2449–57. doi: 10.1182/blood-2009-07-234757 PMC2845901 20097882

[33] Fairfield H Dudakovic A Khatib CM Farrell M Falank C Hinge M. Myeloma-Modified Adipocytes Exhibit Metabolic Dysfunction and a Senescence-Associated Secretory Phenotype (SASP). Cancer Res (2021) 81(3):634–47. doi: 10.1158/0008-5472.CAN-20-1088 PMC7854508 33218968

[34] Wang Z He J Duc-Hiep B Yung-Hsing H Li Z Liu H. Induction of M6a Methylation in Adipocyte Exosomal LncRNAs Mediates Myeloma Drug Resistance. J Exp Clin Cancer Res (2022) 41(1):1–18. doi: 10.1186/s13046-021-02209-w 34980213 PMC8722039

[35] Landgren O Rajkumar SV Pfeiffer RM Kyle RA Katzmann JA Dispenzieri A. Obesity Is Associated With an Increased Risk of Monoclonal Gammopathy of Undetermined Significance Among Black and White Women. Blood (2010) 116(7):1056–9. doi: 10.1182/blood-2010-01-262394 PMC2938127 20421448

[36] Thordardottir M Lindqvist EK Lund SH Costello R Burton D Korde N. Obesity and Risk of Monoclonal Gammopathy of Undetermined Significance and Progression to Multiple Myeloma: A Population-Based Study. Blood Adv (2017) 1(24):2186–92. doi: 10.1182/bloodadvances.2017007609 PMC5737120 29296866

[37] Tsai CK Yeh CM Hsu TL Li CJ Tin C Hsiao LT. Underweight as a Risk Factor of Mortality in Patients With Newly Diagnosed Multiple Myeloma. Support Care Canc (2021) 29(7):3991–9. doi: 10.1007/s00520-020-05849-4 33398428

[38] Allegra A Innao V Allegra AG Pugliese M Di Salvo E Ventura-Spagnolo E. Lymphocyte Subsets and Inflammatory Cytokines of Monoclonal Gammopathy of Undetermined Significance and Multiple Myeloma. Int J Mol Sci (2019) 20(11):1–25. doi: 10.3390/ijms20112822 PMC6600674 31185596

[39] Díaz-Tejedor A Lorenzo-Mohamed M Puig N García-Sanz R Mateos MV Garayoa M. Immune System Alterations in Multiple Myeloma: Molecular Mechanisms and Therapeutic Strategies to Reverse Immunosuppression. Cancers (Basel) (2021) 13(6):1–26. doi: 10.3390/cancers13061353 PMC8002455 33802806

[40] Quezada SA Peggs KS Simpson TR Allison JP. Shifting the Equilibrium in Cancer Immunoediting: From Tumor Tolerance to Eradication. Immunol Rev (2011) 241(1):104–18. doi: 10.1111/j.1600-065X.2011.01007.x PMC3727276 21488893

[41] Schreiber RD Old LJ Smyth MJ. Cancer Immunoediting: Integrating Immunity’s Roles in Cancer Suppression and Promotion. Sci (80- ) (2011) 331(6024):1565–70. doi: 10.1126/science.1203486 21436444

[42] Guillerey C Nakamura K Vuckovic S Hill GR Smyth MJ. Immune Responses in Multiple Myeloma: Role of the Natural Immune Surveillance and Potential of Immunotherapies. Cell Mol Life Sci (2016) 73(8):1569–89. doi: 10.1007/s00018-016-2135-z PMC11108512 26801219

[43] Chauhan D Singh AV Brahmandam M Carrasco R Bandi M Hideshima T. Functional Interaction of Plasmacytoid Dendritic Cells With Multiple Myeloma Cells: A Therapeutic Target. Cancer Cell (2009) 16(4):309–23. doi: 10.1016/j.ccr.2009.08.019 PMC2762396 19800576

[44] Racanelli V Leone P Frassanito MA Brunetti C Perosa F Ferrone S. Alterations in the Antigen Processing-Presenting Machinery of Transformed Plasma Cells are Associated With Reduced Recognition by CD8+ T Cells and Characterize the Progression of MGUS to Multiple Myeloma. Blood (2010) 115(6):1185–93. doi: 10.1182/blood-2009-06-228676 PMC2826230 20008301

[45] Leone P Berardi S Frassanito MA Ria R De Re V Cicco S. Dendritic Cells Accumulate in the Bone Marrow of Myeloma Patients Where They Protect Tumor Plasma Cells From CD8+ T-Cell Killing. Blood (2015) 126(12):1443–51. doi: 10.1182/blood-2015-01-623975 PMC4592278 26185130

[46] Di Marzo L Desantis V Solimando AG Ruggieri S Annese T Nico B. Microenvironment Drug Resistance in Multiple Myeloma: Emerging New Players. Oncotarget (2016) 7(37):60698–711. doi: 10.18632/oncotarget.10849 PMC5312413 27474171

[47] Ahsmann EJM Lokhorst HM Dekker AW Bloem AC. Lymphocyte Function-Associated Antigen-1 Expression on Plasma Cells Correlates With Tumor Growth in Multiple Myeloma. Blood (1992) 79(8):2068–75. doi: 10.1182/blood.V79.8.2068.2068 1562732

[48] Asosingh K Vankerkhove V Van Riet I Van Camp B Vanderkerken K. Selective *In Vivo* Growth of Lymphocyte Function- Associated Antigen-1–Positive Murine Myeloma Cells. Exp Hematol (2003) 31(1):48–55. doi: 10.1016/S0301-472X(02)00970-0 12543106

[49] Tagde A Rajabi H Bouillez A Alam M Gali R Bailey S. MUC1-C Drives MYC in Multiple Myeloma. Blood (2016) 127(21):2587–97. doi: 10.1182/blood-2015-07-659151 PMC4882805 26907633

[50] Chauhan D Uchiyama H Akbarali Y Urashima M Yamamoto KI Libermann TA. Multiple Myeloma Cell Adhesion-Induced Interleukin-6 Expression in Bone Marrow Stromal Cells Involves Activation of NF-κb. Blood (1996) 87(3):1104–12. doi: 10.1182/blood.V87.3.1104.bloodjournal8731104 8562936

[51] Kizaki M Tabayashi T. The Role of Intracellular Signaling Pathways in the Pathogenesis of Multiple Myeloma and Novel Therapeutic Approaches. J Clin Exp Hematop (2016) 56(1):20–7. doi: 10.3960/jslrt.56.20 PMC6144275 27334854

[52] Snapp KR Ding H Atkins K Warnke R Luscinskas FW Kansas GS. A Novel P-Selectin Glycoprotein Ligand-1 Monoclonal Antibody Recognizes an Epitope Within the Tyrosine Sulfate Motif of Human PSGL-1 and Blocks Recognition of Both P- and L-Selectin. Blood (1998) 91(1):154–64. doi: 10.1182/blood.V91.1.154 9414280

[53] Azab AK Quang P Azab F Pitsillides C Thompson B Chonghaile T. P-Selectin Glycoprotein Ligand Regulates the Interaction of Multiple Myeloma Cells With the Bone Marrow Microenvironment. Blood (2012) 119(6):1468–78. doi: 10.1182/blood-2011-07-368050 PMC3286211 22096244

[54] Vacca A Di Loreto M Ribatti D Stefano R Gadaleta-Caldarola G Iodice G. Bone Marrow of Patients With Active Multiple Myeloma: Angiogenesis and Plasma Cell Adhesion Molecules LFA-1, VLA-4, LAM-1, and CD44. Am J Hematol (1995) 50(1):9–14. doi: 10.1002/ajh.2830500103 7545353

[55] Solimando AG da Viá MC Leone P Borrelli P Croci GA Tabares P. Halting the Vicious Cycle Within the Multiple Myeloma Ecosystem: Blocking JAM-A on Bone Marrow Endothelial Cells Restores Angiogenic Homeostasis and Suppresses Tumor Progression. Haematologica (2021) 106(7):1943–56. doi: 10.3324/haematol.2019.239913 PMC8252928 32354870

[56] Bahlis NJ King AM Kolonias D Carlson LM Hong YL Hussein MA. CD28-Mediated Regulation of Multiple Myeloma Cell Proliferation and Survival. Blood (2007) 109(11):5002–10. doi: 10.1182/blood-2006-03-012542 PMC1885531 17311991

[57] Rozanski CH Arens R Carlson LM Nair J Boise LH Chanan-Khan AA. Sustained Antibody Responses Depend on CD28 Function in Bone Marrow-Resident Plasma Cells. J Exp Med (2011) 208(7):1435–46. doi: 10.1084/jem.20110040 PMC3135367 21690252

[58] Wang Q Sun B Wang D Ji Y Kong Q Wang G. Murine Bone Marrow Mesenchymal Stem Cells Cause Mature Dendritic Cells to Promote T-Cell Tolerance. Scand J Immunol (2008) 68(6):607–15. doi: 10.1111/j.1365-3083.2008.02180.x 18959624

[59] Murray ME Gavile CM Nair JR Koorella C Carlson LM Buac D. CD28-Mediated Pro-Survival Signaling Induces Chemotherapeutic Resistance in Multiple Myeloma. Blood (2014) 123(24):3770–9. doi: 10.1182/blood-2013-10-530964 PMC4055924 24782505

[60] Gavile CM Barwick BG Newman S Neri P Nooka AK Lonial S. CD86 Regulates Myeloma Cell Survival. Blood Adv (2017) 1(25):2307–19. doi: 10.1182/bloodadvances.2017011601 PMC5729629 29296880

[61] Ray A Das DS Song Y Richardson P Munshi NC Chauhan D. Targeting PD1-PDL1 Immune Checkpoint in Plasmacytoid Dendritic Cell Interactions With T Cells, Natural Killer Cells and Multiple Myeloma Cells. Leukemia (2015) 29(6):1441–4. doi: 10.1038/leu.2015.11 PMC5703039 25634684

[62] Sponaas AM Moharrami NN Feyzi E Standal T Rustad EH Waage A. PDL1 Expression on Plasma and Dendritic Cells in Myeloma Bone Marrow Suggests Benefit of Targeted Anti PD1-PDL1 Therapy. PloS One (2015) 10(10):1–8. doi: 10.1371/journal.pone.0139867 PMC4596870 26444869

[63] Castella B Foglietta M Riganti C Massaia M. Vγ9vδ2 T Cells in the Bone Marrow of Myeloma Patients: A Paradigm of Microenvironment-Induced Immune Suppression. Front Immunol (2018) 9:1492. doi: 10.3389/fimmu.2018.01492 30013559 PMC6036291

[64] Benson DM Bakan CE Mishra A Hofmeister CC Efebera Y Becknell B. The PD-1/PD-L1 Axis Modulates the Natural Killer Cell Versus Multiple Myeloma Effect: A Therapeutic Target for CT-011, a Novel Monoclonal Anti-PD-1 Antibody. Blood (2010) 116(13):2286–94. doi: 10.1182/blood-2010-02-271874 PMC3490105 20460501

[65] Kim J Denu RA Dollar BA Escalante LE Kuether JP Callander NS. Macrophages and Mesenchymal Stromal Cells Support Survival and Proliferation of Multiple Myeloma Cells. Br J Haematol (2012) 158(3):336–46. doi: 10.1111/j.1365-2141.2012.09154.x PMC3395762 22583117

[66] Zheng Y Cai Z Wang S Zhang X Qian J Hong S. Macrophages are an Abundant Component of Myeloma Microenvironment and Protect Myeloma Cells From Chemotherapy Drug-Induced Apoptosis. Blood (2009) 114(17):3625–8. doi: 10.1182/blood-2009-05-220285 PMC2766678 19710503

[67] Zheng Y Yang J Qian J Qiu P Hanabuchi S Lu Y. PSGL-1/Selectin and ICAM-1/CD18 Interactions are Involved in Macrophage-Induced Drug Resistance in Myeloma. Leukemia (2013) 27(3):702–10. doi: 10.1038/leu.2012.272 PMC3652581 22996336

[68] Frassanito MA Ruggieri S Desantis V Di Marzo L Leone P Racanelli V. Myeloma Cells Act as Tolerogenic Antigen-Presenting Cells and Induce Regulatory T Cells *In Vitro* . Eur J Haematol (2015) 95(1):65–74. doi: 10.1111/ejh.12481 25409753

[69] Feyler S Scott GB Parrish C Jarmin S Evans P Short M. Tumour Cell Generation of Inducible Regulatory T-Cells in Multiple Myeloma is Contact-Dependent and Antigen-Presenting Cell-Independent. PloS One (2012) 7(5):1–10. doi: 10.1371/journal.pone.0035981 PMC3362588 22666318

[70] Leone P Solimando AG Malerba E Fasano R Buonavoglia A Pappagallo F. Actors on the Scene: Immune Cells in the Myeloma Niche. Front Oncol (2020) 10:599098. doi: 10.3389/fonc.2020.599098 33194767 PMC7658648

[71] Krejcik J Barnkob MB Nyvold CG Larsen TS Barington T Abildgaard N. Harnessing the Immune System to Fight Multiple Myeloma. Cancers (Basel) (2021) 13(18):4546. doi: 10.3390/cancers13184546 34572773 PMC8467095

[72] Moschoi R Imbert V Nebout M Chiche J Mary D Prebet T. Protective Mitochondrial Transfer From Bone Marrow Stromal Cells to Acute Myeloid Leukemic Cells During Chemotherapy. Blood (2016) 128(2):253–64. doi: 10.1182/blood-2015-07-655860 27257182

[73] Marlein CR Zaitseva L Piddock RE Robinson SD Edwards DR Shafat MS. NADPH Oxidase-2 Derived Superoxide Drives Mitochondrial Transfer From Bone Marrow Stromal Cells to Leukemic Blasts. Blood (2017) 130(14):1649–60. doi: 10.1182/blood-2017-03-772939 28733324

[74] Wang J Liu X Qiu Y Shi Y Cai J Wang B. Cell Adhesion-Mediated Mitochondria Transfer Contributes to Mesenchymal Stem Cell-Induced Chemoresistance on T Cell Acute Lymphoblastic Leukemia Cells. J Hematol Oncol (2018) 11(1):1–13. doi: 10.1186/s13045-018-0554-z 29357914 PMC5778754

[75] Marlein CR Piddock RE Mistry JJ Zaitseva L Hellmich C Horton RH. CD38-Driven Mitochondrial Trafficking Promotes Bioenergetic Plasticity in Multiple Myeloma. Cancer Res (2019) 79(9):2285–97. doi: 10.1158/0008-5472.CAN-18-0773 30622116

[76] Giallongo C Dulcamare I Tibullo D Del Fabro V Vicario N Parrinello N. CXCL12/CXCR4 Axis Supports Mitochondrial Trafficking in Tumor Myeloma Microenvironment. Oncogenesis (2022) 11(1):1–13. doi: 10.1038/s41389-022-00380-z 35064098 PMC8782911

[77] Matula Z Mikala G Lukácsi S Matkó J Kovács T Monostori É. Stromal Cells Serve Drug Resistance for Multiple Myeloma *via* Mitochondrial Transfer: A Study on Primary Myeloma and Stromal Cells. Cancers (Basel) (2021) 13(14):3461. doi: 10.3390/cancers13143461 34298674 PMC8307863

[78] Hideshima T Nakamura N Chauhan D Anderson KC. Biologic Sequelae of Interleukin-6 Induced PI3-K/Akt Signaling in Multiple Myeloma. Oncogene (2001) 20(42):5991–6000. doi: 10.1038/sj.onc.1204833 11593406

[79] Uchiyama H Barut BA Mohrbacher AF Chauhan D Anderson KC. Adhesion of Human Myeloma-Derived Cell Lines to Bone Marrow Stromal Cells Stimulates Interleukin-6 Secretion. Blood (1993) 82(12):3712–20. doi: 10.1182/blood.V82.12.3712.3712 8260708

[80] Gupta VA Matulis SM Conage-Pough JE Nooka AK Kaufman JL Lonial S. Bone Marrow Microenvironment–Derived Signals Induce Mcl-1 Dependence in Multiple Myeloma. Blood (2017) 129(14):1969–79. doi: 10.1182/blood-2016-10-745059 PMC5383873 28151428

[81] Conage-Pough JE Boise LH. Phosphorylation Alters Bim-Mediated Mcl-1 Stabilization and Priming. FEBS J (2018) 285(14):2626–40. doi: 10.1111/febs.14505 PMC6215700 29775995

[82] Tai YT Dillon M Song W Leiba M Li XF Burger P. Anti-CSl Humanized Monoclonal Antibody HuLuc63 Inhibits Myeloma Cell Adhesion and Induces Antibody-Dependent Cellular Cytotoxicity in the Bone Marrow Milieu. Blood (2008) 112(4):1329–37. doi: 10.1182/blood-2007-08-107292 PMC2515112 17906076

[83] Kikuchi J Hori M Iha H Toyama-Sorimachi N Hagiwara S Kuroda Y. Soluble SLAMF7 Promotes the Growth of Myeloma Cells *via* Homophilic Interaction With Surface SLAMF7. Leukemia (2020) 34(1):180–95. doi: 10.1038/s41375-019-0525-6 31358854

[84] Schneider P Mackay F Steiner V Hofmann K Bodmer JL Holler N. BAFF, a Novel Ligand of the Tumor Necrosis Factor Family, Stimulates B Cell Growth. J Exp Med (1999) 189(11):1747–56. doi: 10.1084/jem.189.11.1747 PMC2193079 10359578

[85] Novak AJ Darce JR Arendt BK Harder B Henderson K Kindsvogel W. Expression of BCMA, TACI, and BAFF-R in Multiple Myeloma: A Mechanism for Growth and Survival. Blood (2004) 103(2):689–94. doi: 10.1182/blood-2003-06-2043 14512299

[86] Tai YT Acharya C An G Moschetta M Zhong MY Feng X. APRIL and BCMA Promote Human Multiple Myeloma Growth and Immunosuppression in the Bone Marrow Microenvironment. Blood (2016) 127(25):3225–36. doi: 10.1182/blood-2016-01-691162 PMC4920023 27127303

[87] de Jong MME Kellermayer Z Papazian N Tahri S Hofste D Hoogenboezem R. The Multiple Myeloma Microenvironment is Defined by an Inflammatory Stromal Cell Landscape. Nat Immunol (2021) 22(6):769–80. doi: 10.1038/s41590-021-00931-3 34017122

[88] Hideshima T Chauhan D Schlossman R Richardson P Anderson KC. The Role of Tumor Necrosis Factor α in the Pathophysiology of Human Multiple Myeloma: Therapeutic Applications. Oncogene (2001) 20(33):4519–27. doi: 10.1038/sj.onc.1204623 11494147

[89] Lee C Oh JI Park J Choi JH Bae EK Lee HJ. TNF α Mediated IL-6 Secretion Is Regulated by JAK/STAT Pathway But Not by MEK Phosphorylation and AKT Phosphorylation in U266 Multiple Myeloma Cells. BioMed Res Int (2013) 2013:580135. doi: 10.1155/2013/580135 24151609 PMC3787550

[90] Mitsiades CS Mitsiades NS McMullan CJ Poulaki V Shringarpure R Akiyama M. Inhibition of the Insulin-Like Growth Factor Receptor-1 Tyrosine Kinase Activity as a Therapeutic Strategy for Multiple Myeloma, Other Hematologic Malignancies, and Solid Tumors. Cancer Cell (2004) 5(3):221–30. doi: 10.1016/S1535-6108(04)00050-9 15050914

[91] Bieghs L Johnsen HE Maes K Menu E Van Valckenborgh E Overgaard MT. The Insulin-Like Growth Factor System in Multiple Myeloma: Diagnostic and Therapeutic Potential. Oncotarget (2016) 7(30):48732–52. doi: 10.18632/oncotarget.8982 PMC5217049 27129151

[92] Choi SJ Oba Y Gazitt Y Alsina M Cruz J Anderson J. Antisense Inhibition of Macrophage Inflammatory Protein 1-α Blocks Bone Destruction in a Model of Myeloma Bone Disease. J Clin Invest (2001) 108(12):1833–41. doi: 10.1172/JCI200113116 PMC209465 11748267

[93] Oba Y Lee JW Ehrlich LA Chung HY Jelinek DF Callander NS. MIP-1α Utilizes Both CCR1 and CCR5 to Induce Osteoclast Formation and Increase Adhesion of Myeloma Cells to Marrow Stromal Cells. Exp Hematol (2005) 33(3):272–8. doi: 10.1016/j.exphem.2004.11.015 15730850

[94] Man JH Choi SJ Kurihara N Koide M Oba Y David Roodman G. Macrophage Inflammatory Protein-1α is an Osteoclastogenic Factor in Myeloma That Is Independent of Receptor Activator of Nuclear Factor κb Ligand. Blood (2001) 97(11):3349–53. doi; 10.1182/blood.v97.11.3349 11369623

[95] Yin L. Chondroitin Synthase 1 is a Key Molecule in Myeloma Cell-Osteoclast Interactions. J Biol Chem (2005) 280(16):15666–72. doi: 10.1074/jbc.M409877200 15485809

[96] Vallet S Mukherjee S Vaghela N Hideshima T Fulciniti M Pozzi S. Activin A Promotes Multiple Myeloma-Induced Osteolysis and Is a Promising Target for Myeloma Bone Disease. Proc Natl Acad Sci USA (2010) 107(11):5124–9. doi: 10.1073/pnas.0911929107 PMC2841922 20194748

[97] Ehrlich LA Chung HY Ghobrial I Choi SJ Morandi F Colla S. EL-3 Is a Potential Inhibitor of Osteoblast Differentiation in Multiple Myeloma. Blood (2005) 106(4):1407–14. doi: 10.1182/blood-2005-03-1080 15878977

[98] Pearse RN Sordillo EM Yaccoby S Wong BR Liau DF Colman N. Multiple Myeloma Disrupts the TRANCE/osteoprotegerin Cytokine Axis to Trigger Bone Destruction and Promote Tumor Progression. Proc Natl Acad Sci USA (2001) 98(20):11581–6. doi: 10.1073/pnas.201394498 PMC58772 11562486

[99] Terpos E Efstathiou E Christoulas D Roussou M Katodritou E Dimopoulos MA. RANKL Inhibition: Clinical Implications for the Management of Patients With Multiple Myeloma and Solid Tumors With Bone Metastases. Expert Opin Biol Ther (2009) 9(4):465–79. doi: 10.1517/14712590902845610 19344283

[100] Sugatani T Alvarez UM Hruska KA. Activin A Stimulates Iκb-α/Nfκb and RANK Expression for Osteoclast Differentiation, But Not AKT Survival Pathway in Osteoclast Precursors. J Cell Biochem (2003) 90(1):59–67. doi: 10.1002/jcb.10613 12938156

[101] Terpos E Kastritis E Christoulas D Gkotzamanidou M Eleutherakis-Papaiakovou E Kanellias N. Circulating Activin-A Is Elevated in Patients With Advanced Multiple Myeloma and Correlates With Extensive Bone Involvement and Inferior Survival; No Alterations Post-Lenalidomide and Dexamethasone Therapy. Ann Oncol (2012) 23(10):2681–6. doi: 10.1093/annonc/mds068 22492699

[102] Poole KES Van Bezooijen RL Loveridge N Hamersma H Papapoulos SE Löwik CW. Sclerostin is a Delayed Secreted Product of Osteocytes That Inhibits Bone Formation. FASEB J (2005) 19(13):1842–4. doi: 10.1096/fj.05-4221fje 16123173

[103] Toscani D Bolzoni M Ferretti M Palumbo C Giuliani N. Role of Osteocytes in Myeloma Bone Disease: Anti-Sclerostin Antibody as New Therapeutic Strategy. Front Immunol (2018) 9:2467. doi: 10.3389/fimmu.2018.02467 30410490 PMC6209728

[104] Boyle WJ Simonet WS Lacey DL. Osteoclast Differentiation and Activation. Nature (2003) 423(6937):337–42. doi: 10.1038/nature01658 12748652

[105] Hideshima T Mitsiades C Tonon G Richardson PG Anderson KC. Understanding Multiple Myeloma Pathogenesis in the Bone Marrow to Identify New Therapeutic Targets. Nat Rev Canc (2007) 7(8):585–98. doi: 10.1038/nrc2189 17646864

[106] Ribatti D Nico B Vacca A. Importance of the Bone Marrow Microenvironment in Inducing the Angiogenic Response in Multiple Myeloma. Oncogene (2006) 25(31):4257–66. doi: 10.1038/sj.onc.1209456 16518413

[107] Berardi S Ria R Reale A De Luisi A Catacchio I Moschetta M. Multiple Myeloma Macrophages: Pivotal Players in the Tumor Microenvironment. J Oncol (2013) 2013:183602. doi: 10.1155/2013/183602 23431298 PMC3570938

[108] Pratt G Goodyear O Moss P. Immunodeficiency and Immunotherapy in Multiple Myeloma. Br J Haematol (2007) 138(5):563–79. doi: 10.1111/j.1365-2141.2007.06705.x 17686051

[109] Nair JR Rozanski CH Lee KP. Under One Roof the Bone Marrow Survival Niche for Multiple Myeloma and Normal Plasma Cells. Oncoimmunology (2012) 1(3):388–9. doi: 10.4161/onci.18746 PMC3382851 22737625

[110] Suen H Brown R Yang S Weatherburn C Ho PJ Woodland N. Multiple Myeloma Causes Clonal T-Cell Immunosenescence: Identification of Potential Novel Targets for Promoting Tumour Immunity and Implications for Checkpoint Blockade. Leukemia (2016) 30(8):1716–24. doi: 10.1038/leu.2016.84 27102208

[111] Bryant C Suen H Brown R Yang S Favaloro J Aklilu E. Long-Term Survival in Multiple Myeloma is Associated With a Distinct Immunological Profile, Which Includes Proliferative Cytotoxic T-Cell Clones and a Favourable Treg/Th17 Balance. Blood Cancer J (2013) 3(9):1–7. doi: 10.1038/bcj.2013.34 PMC3789202 24036947

[112] Noonan K Marchionni L Anderson J Pardoll D Roodman GD Borrello I. A Novel Role of IL-17-Producing Lymphocytes in Mediating Lytic Bone Disease in Multiple Myeloma. Blood (2010) 116(18):3554–63. doi: 10.1182/blood-2010-05-283895 PMC4017298 20664052

[113] Prabhala RH Pelluru D Fulciniti M Prabhala HK Nanjappa P Song W. Elevated IL-17 Produced by TH17 Cells Promotes Myeloma Cell Growth and Inhibits Immune Function in Multiple Myeloma. Blood (2010) 115(26):5385–92. doi: 10.1182/blood-2009-10-246660 PMC2902136 20395418

[114] Shen CJ Yuan ZH Liu YX Hu GY. Increased Numbers of T Helper 17 Cells and the Correlation With Clinicopathological Characteristics in Multiple Myeloma. J Int Med Res (2012) 40(2):556–64. doi: 10.1177/147323001204000217 22613416

[115] Görgün GT Whitehill G Anderson JL Hideshima T Maguire C Laubach J. Tumor-Promoting Immune-Suppressive Myeloid-Derived Suppressor Cells in the Multiple Myeloma Microenvironment in Humans. Blood (2013) 121(15):2975–87. doi; 10.1182/blood-2012-08-448548 PMC3624943 23321256

[116] Ramachandran IR Martner A Pisklakova A Condamine T Chase T Vogl T. Myeloid-Derived Suppressor Cells Regulate Growth of Multiple Myeloma by Inhibiting T Cells in Bone Marrow. J Immunol (2013) 190(7):3815–23. doi: 10.4049/jimmunol.1203373 PMC3608837 23460744

[117] Rodriguez PC Quiceno DG Zabaleta J Ortiz B Zea AH Piazuelo MB. Arginase I Production in the Tumor Microenvironment by Mature Myeloid Cells Inhibits T-Cell Receptor Expression and Antigen-Specific T-Cell Responses. Cancer Res (2004) 64(16):5839–49. doi: 10.1158/0008-5472.CAN-04-0465 15313928

[118] Serafini P Borrello I Bronte V. Myeloid Suppressor Cells in Cancer: Recruitment, Phenotype, Properties, and Mechanisms of Immune Suppression. Semin Cancer Biol (2006) 16(1):53–65. doi: 10.1016/j.semcancer.2005.07.005 16168663

[119] Castella B Foglietta M Sciancalepore P Rigoni M Coscia M Griggio V. Anergic Bone Marrow Vγ9vδ2 T Cells as Early and Long-Lasting Markers of PD-1-Targetable Microenvironment-Induced Immune Suppression in Human Myeloma. Oncoimmunology (2015) 4(11):e1047580. doi: 10.1080/2162402X.2015.1047580 26451323 PMC4589055

[120] Li H Han Y Guo Q Zhang M Cao X. Cancer-Expanded Myeloid-Derived Suppressor Cells Induce Anergy of NK Cells Through Membrane-Bound TGF-β1. J Immunol (2009) 182(1):240–9. doi: 10.4049/jimmunol.182.1.240 19109155

[121] Karle H Hansen NE PT. Studies on Intraneutrophilic Lysozyme in Multiple Myeloma and Malignant Lymphoma. Scand J Haematol (1976) 17(1):62–70. 959776

[122] Romano A Parrinello NL La Cava P Tibullo D Giallongo C Camiolo G. PMN-MDSC and Arginase are Increased in Myeloma and may Contribute to Resistance to Therapy. Expert Rev Mol Diagn (2018) 18(7):675–83. doi: 10.1080/14737159.2018.1470929 29707981

[123] Puglisi F Parrinello NL Giallongo C Cambria D Camiolo G Bellofiore C. Plasticity of High-Density Neutrophils in Multiple Myeloma is Associated With Increased Autophagy *via* STAT3. Int J Mol Sci (2019) 20(14):1–17. doi: 10.3390/ijms20143548 PMC6678548 32565533

[124] Fowler JA Lwin ST Drake MT Edwards JR Kyle RA Mundy GR. Host-Derived Adiponectin Is Tumor-Suppressive and a Novel Therapeutic Target for Multiple Myeloma and the Associated Bone Disease. Blood (2011) 118:5872–82. doi: 10.1182/blood-2011-01-330407 PMC3228502 21908434

[125] Fan Y Hanai J-I Le PT Bi R Maridas D DeMambro V. Parathyroid Hormone Directs Bone Marrow Mesenchymal Cell Fate. Cell Metab (2017) 25(3):661–72. doi: 10.1016/j.cmet.2017.01.001 PMC5342925 28162969

[126] Boyd AL Reid JC Salci KR Aslostovar L Benoit YD Shapovalova Z. Acute Myeloid Leukaemia Disrupts Endogenous Myelo-Erythropoiesis by Compromising the Adipocyte Bone Marrow Niche. Nat Cell Biol (2017) 19(11):1336–47. doi: 10.1038/ncb3625 29035359

[127] Greco EA Lenzi A Migliaccio S. The Obesity of Bone. Ther Adv Endocrinol Metab (2015) 6(6):273–86. doi: 10.1177/2042018815611004 PMC4647134 26623005

[128] Sakurai T Ogasawara J Kizaki T Sato S Ishibashi Y Takahashi M. The Effects of Exercise Training on Obesity-Induced Dysregulated Expression of Adipokines in White Adipose Tissue. Int J Endocrinol (2013) 2013:801743. doi: 10.1155/2013/801743 24369466 PMC3867917

[129] Medina EA Oberheu K Polusani SR Ortega V Velagaleti GVN Oyajobi BO. PKA/AMPK Signaling in Relation to Adiponectin’s Antiproliferative Effect on Multiple Myeloma Cells. Leukemia (2014) 28(10):2080–9. doi: 10.1038/leu.2014.112 24646889

[130] Morris EV Suchacki KJ Hocking J Cartwright R Sowman A Gamez B. Myeloma Cells Down-Regulate Adiponectin in Bone Marrow Adipocytes *Via* TNF-Alpha. J Bone Min Res (2020) 35(5):942–55. doi: 10.1002/jbmr.3951 PMC9328417 31886918

[131] Yu W Cao DD Li Q-B Mei H-L Hu Y Guo T. Adipocytes Secreted Leptin is a Pro-Tumor Factor for Survival of Multiple Myeloma Under Chemotherapy. Oncotarget (2016) 7(52):86075–86. doi: 10.18632/oncotarget.13342 PMC5349898 27863383

[132] Favreau M Menu E Gaublomme D Vanderkerken K Faict S Maes K. Leptin Receptor Antagonism of iNKT Cell Function: A Novel Strategy to Combat Multiple Myeloma. Leukemia (2017) 31(12):2678–85. doi: 10.1038/leu.2017.146 28490813

[133] Liu Z Xu J He J Liu H Lin P Wan X. Mature Adipocytes in Bone Marrow Protect Myeloma Cells Against Chemotherapy Through Autophagy Activation. Oncotarget (2015) 6(33):34329–41. doi: 10.18632/oncotarget.6020 PMC4741456 26455377

[134] Jöhrer K Janke K Krugmann J Fiegl M Greil R. Transendothelial Migration of Myeloma Cells Is Increased by Tumor Necrosis Factor (TNF)-α *via* TNF Receptor 2 and Autocrine Up-Regulation of MCP-1. Clin Cancer Res (2004) 10(6):1901–10. doi: 10.1158/1078-0432.CCR-1053-03 15041705

[135] Westhrin M Moen SH Kristensen IB Buene G Mylin AK Turesson I. Chemerin is Elevated in Multiple Myeloma Patients and is Expressed by Stromal Cells and Pre-Adipocytes. Biomark Res (2018) 6(1):4–7. doi: 10.1186/s40364-018-0134-y 29946468 PMC6001014

[136] Pang J Shi Q Liu Z He J Liu H Lin P. Resistin Induces Multidrug Resistance in Myeloma by Inhibiting Cell Death and Upregulating ABC Transporter Expression. Haematologica (2017) 102(7):1273–80. doi: 10.3324/haematol.2016.154062 PMC5566043 28360146

[137] Van Niel G D’Angelo G Raposo G. Shedding Light on the Cell Biology of Extracellular Vesicles. Nat Rev Mol Cell Biol (2018) 19(4):213–28. doi: 10.1038/nrm.2017.125 29339798

[138] Abels ER Breakefield XO. Introduction to Extracellular Vesicles: Biogenesis, RNA Cargo Selection, Content, Release, and Uptake. Cell Mol Neurobiol (2016) 36(3):301–12. doi: 10.1007/s10571-016-0366-z PMC5546313 27053351

[139] Mulcahy LA Pink RC Carter DRF. Routes and Mechanisms of Extracellular Vesicle Uptake. J Extrac Vesicles (2014) 3(1). doi: 10.3402/jev.v3.24641 PMC4122821 25143819

[140] Boyiadzis M Whiteside TL. The Emerging Roles of Tumor-Derived Exosomes in Hematological Malignancies. Leukemia (2017) 31(6):1259–68. doi: 10.1038/leu.2017.91 28321122

[141] Zhang HG Grizzle WE. Exosomes: A Novel Pathway of Local and Distant Intercellular Communication That Facilitates the Growth and Metastasis of Neoplastic Lesions. Am J Pathol (2014) 184(1):28–41. doi: 10.1016/j.ajpath.2013.09.027 24269592 PMC3873490

[142] Moloudizargari M Abdollahi M Asghari MH Zimta AA Neagoe IB Nabavi SM. The Emerging Role of Exosomes in Multiple Myeloma. Blood Rev (2019) 38:100595. doi: 10.1016/j.blre.2019.100595 31445775

[143] Roccaro AM Sacco A Maiso P Azab AK Tai YT Reagan M. BM Mesenchymal Stromal Cell-Derived Exosomes Facilitate Multiple Myeloma Progression. J Clin Invest (2013) 123(4):1542–55. doi: 10.1172/JCI66517 PMC3613927 23454749

[144] Wang J Hendrix A Hernot S Lemaire M De Bruyne E Van Valckenborgh E. Bone Marrow Stromal Cell-Derived Exosomes as Communicators in Drug Resistance in Multiple Myeloma Cells. Blood (2014) 124(4):555–66. doi: 10.1182/blood-2014-03-562439 24928860

[145] De Veirman K Wang J Xu S Leleu X Himpe E Maes K. Induction of miR-146a by Multiple Myeloma Cells in Mesenchymal Stromal Cells Stimulates Their Pro-Tumoral Activity. Cancer Lett (2016) 377(1):17–24. doi: 10.1016/j.canlet.2016.04.024 27102001

[146] Theocharis AD Skandalis SS Gialeli C Karamanos NK. Extracellular Matrix Structure. Adv Drug Deliv Rev (2016) 97:4–27. doi: 10.1016/j.addr.2015.11.001 26562801

[147] Bonnans C Chou J Werb Z. Remodelling the Extracellular Matrix in Development and Disease. Nat Rev Mol Cell Biol (2014) 15(12):786–801. doi: 10.1038/nrm3904 25415508 PMC4316204

[148] Abedin M King N. Diverse Evolutionary Paths to Cell Adhesion. Trends Cell Biol (2010) 20(12):734–42. doi: 10.1016/j.tcb.2010.08.002 PMC2991404 20817460

[149] Hosen N. Integrins in Multiple Myeloma. Inflamm Regen (2020) 40(1):20–3. doi: 10.1186/s41232-020-00113-y PMC7104491 32256871

[150] Neri P Ren L Azab AK Brentnall M Gratton K Klimowicz AC. Integrin β7-Mediated Regulation of Multiple Myeloma Cell Adhesion, Migration, and Invasion. Blood (2011) 117(23):6202–13. doi: 10.1182/blood-2010-06-292243 PMC3122944 21474670

[151] Landowski TH Olashaw NE Agrawal D Dalton WS. Cell Adhesion-Mediated Drug Resistance (CAM-DR) Is Associated With Activation of NF-κb (RelB/p50) in Myeloma Cells. Oncogene (2003) 22(16):2417–21. doi: 10.1038/sj.onc.1206315 12717418

[152] Hosen N Matsunaga Y Hasegawa K Matsuno H Nakamura Y Makita M. The Activated Conformation of Integrin β7 Is a Novel Multiple Myeloma-Specific Target for CAR T Cell Therapy. Nat Med (2017) 23(12):1436–43. doi: 10.1038/nm.4431 29106400

[153] Riet IV Waele M Remels L Lacor P Schots R Van C B. Expression of Cytoadhesion Molecules (CD56, CD54, CD18 and CD29) by Myeloma Plasma Cells. Br J Haematol (1991) 79(3):421–7. doi: 10.1111/j.1365-2141.1991.tb08050.x 1721526

[154] Sanderson RD Turnbull JE Gallagher JT Lander AD. Fine Structure of Heparan Sulfate Regulates Syndecan-1 Function and Cell Behavior. J Biol Chem (1994) 269(18):13100–6. doi: 10.1016/S0021-9258(17)36804-7 8175735

[155] Reijmers RM Groen RWJ Rozemuller H Kuil A De Haan-Kramer A Csikós T. Targeting EXT1 Reveals a Crucial Role for Heparan Sulfate in the Growth of Multiple Myeloma. Blood (2010) 115(3):601–4. doi: 10.1182/blood-2009-02-204396 19965677

[156] Yang Y Yaccoby S Liu W Kevin Langford J Pumphrey CY Theus A. Soluble Syndecan-1 Promotes Growth of Myeloma Tumors *In Vivo* . Blood (2002) 100(2):610–7. doi: 10.1182/blood.V100.2.610 12091355

[157] Katz BZ. Adhesion Molecules-The Lifelines of Multiple Myeloma Cells. Semin Cancer Biol (2010) 20(3):186–95. doi: 10.1016/j.semcancer.2010.04.003 20416379

[158] Hatano K Kikuchi J Takatoku M Shimizu R Wada T Ueda M. Bortezomib Overcomes Cell Adhesion-Mediated Drug Resistance Through Downregulation of VLA-4 Expression in Multiple Myeloma. Oncogene (2009) 28(2):231–42. doi: 10.1038/onc.2008.385 18850009

[159] Möller C Strömberg T Juremalm M Nilsson K Nilsson G. Expression and Function of Chemokine Receptors in Human Multiple Myeloma. Leukemia (2003) 17(1):203–10. doi: 10.1038/sj.leu.2402717 12529679

[160] Menu E Asosingh K Indraccolo S De Raeve H Van Riet I Van Valckenborgh E. The Involvement of Stromal Derived Factor 1α in Homing and Progression of Multiple Myeloma in the 5TMM Model. Haematologica (2006) 91(5):605–12. 16627256

[161] Alsayed Y Ngo H Runnels J Leleu X Singha UK Pitsillides CM. Mechanisms of Regulation of CXCR4/SDF-1 (CXCL12)-Dependent Migration and Homing in Multiple Myeloma. Blood (2007) 109(7):2708–17. doi: 10.1182/blood-2006-07-035857 PMC1852222 17119115

[162] Sanz-Rodríguez F Hidalgo A Teixidó J. Chemokine Stromal Cell-Derived Factor-1α Modulates VLA-4 Integrin-Mediated Multiple Myeloma Cell Adhesion to CS-1/Fibronectin and VCAM-1. Blood (2001) 97(2):346–51. doi: 10.1182/blood.V97.2.346 11154207

[163] Martnez-Moreno M Leiva M Aguilera-Montilla N Sevilla-Movilla S Isern de Val S Arellano-Snchez N. *In Vivo* Adhesion of Malignant B Cells to Bone Marrow Microvasculature Is Regulated by α4β1 Cytoplasmic-Binding Proteins. Leukemia (2016) 30(4):861–72. doi: 10.1038/leu.2015.332 26658839

[164] Podar K Zimmerhackl A Fulciniti M Tonon G Hainz U Tai YT. The Selective Adhesion Molecule Inhibitor Natalizumab Decreases Multiple Myeloma Cell Growth in the Bone Marrow Microenvironment: Therapeutic Implications. Br J Haematol (2011) 155(4):438–48. doi: 10.1111/j.1365-2141.2011.08864.x 21923653

[165] Azab AK Runnels JM Pitsillides C Moreau AS Azab F Leleu X. CXCR4 Inhibitor AMD3100 Disrupts the Interaction of Multiple Myeloma Cells With the Bone Marrow Microenvironment and Enhances Their Sensitivity to Therapy. Blood (2009) 113(18):4341–51. doi: 10.1182/blood-2008-10-186668 PMC2676090 19139079

[166] Hargreaves DC Hyman PL Lu TT Ngo VN Bidgol A Suzuki G. A Coordinated Change in Chemokine Responsiveness Guides Plasma Cell Movements. J Exp Med (2001) 194(1):45–56. doi: 10.1084/jem.194.1.45 11435471 PMC2193440

[167] Elmentaite R Kumasaka N Roberts K Fleming A Dann E King HW. Cells of the Human Intestinal Tract Mapped Across Space and Time. Nature (2021) 597(7875):250–5. doi: 10.1038/s41586-021-03852-1 PMC8426186 34497389

[168] Natoni A Farrell ML Harris S Falank C Kirkham-McCarthy L Macauley MS. Sialyltransferase Inhibition Leads to Inhibition of Tumor Cell Interactions With E-Selectin, VCAM1, and MADCAM1, and Improves Sur Vival in a Human Multiple Myeloma Mouse Model. Haematologica (2020) 105(2):457–67. doi: 10.3324/haematol.2018.212266 PMC7012485 31101754

[169] Tohami T Drucker L Shapiro H Radnay J Lishner M. Overexpression of Tetraspanins Affects Multiple Myeloma Cell Survival and Invasive Potential. FASEB J (2007) 21(3):691–9. doi: 10.1096/fj.06-6610com 17210782

[170] Bianchi G Czarnecki PG Ho M Roccaro AM Sacco A Kawano Y. ROBO1 Promotes Homing, Dissemination, and Survival of Multiple Myeloma Within the Bone Marrow Microenvironment. Cancer Discov (2021) 2(4):338–53. doi: 10.1158/2643-3230.BCD-20-0164 PMC8265993 34268498

[171] Bustany S Bourgeais J Tchakarska G Body S Herault O Gouilleux F. Cyclin D1 Expression in Myeloma Cells Alters Various Cell Functions Cyclin D1 Increases Cell Adhesion and Migration, and Chemokine Secretion. Oncotarget (2016) 7(29):45214–24. doi: 10.18632/oncotarget.9901 PMC5216717 27286258

[172] Vande Broek I Leleu X Schots R Facon T Vanderkerken K Van Camp B VRI. Clinical Significance of Chemokine Receptor (CCR1, CCR2 and CXCR4) Expression in Human Myeloma Cells: The Association With Disease Activity and Survival. Haematologica (2007) 92(11):1584. 16461304

[173] Vandyke K Zeissig MN Hewett DR Martin SK Mrozik KM Cheong CM. HIF-2α Promotes Dissemination of Plasma Cells in Multiple Myeloma by Regulating CXCL12/CXCR4 and CCR1. Cancer Res (2017) 77(20):5452–63. doi: 10.1158/0008-5472.CAN-17-0115 28855206

[174] Akhmetzyanova I McCarron MJ Parekh S Chesi M Bergsagel PL Fooksman DR. Dynamic CD138 Surface Expression Regulates Switch Between Myeloma Growth and Dissemination. Leukemia (2020) 34(1):245–56. doi: 10.1038/s41375-019-0519-4 PMC6923614 31439945

[175] Sevcikova T Kryukov F Brozova L Filipova J Kufova Z Growkova K. Gene Expression Profile of Circulating Myeloma Cells Reveals CD44 and CD97 (ADGRE5) Overexpression. Blood (2016) 128(22):5639. doi: 10.1182/blood.V128.22.5639.5639

[176] Roccaro AM Mishima Y Sacco A Moschetta M Tai YT Shi J. CXCR4 Regulates Extra-Medullary Myeloma Through Epithelial-Mesenchymal-Transition-Like Transcriptional Activation. Cell Rep (2015) 12(4):622–35. doi: 10.1016/j.celrep.2015.06.059 PMC4961259 26190113

[177] Dahl IMS Rasmussen T Husebekk A Kauric G. Differential Expression of CD56 and CD44 in the Evolution of Extramedullary Myeloma. Br J Haematol (2002) 116(2):273–7. doi: 10.1046/j.1365-2141.2002.03258.x 11841427

[178] Usmani SZ Heuck C Mitchell A Szymonifka J Nair B Hoering A. Extramedullary Disease Portends Poor Prognosis in Multiple Myeloma and Is Over-Represented in High-Risk Disease Even in the Era of Novel Agents. Haematologica (2012) 97(11):1761–7. doi: 10.3324/haematol.2012.065698 PMC3487453 22689675

[179] Kirk SJ Cliff JM Thomas JA Ward TH. Biogenesis of Secretory Organelles During B Cell Differentiation. J Leukoc Biol (2010) 87(2):245–55. doi: 10.1189/jlb.1208774 19889725

[180] Cenci S Mezghrani A Cascio P Bianchi G Cerruti F Fra A. Progressively Impaired Proteasomal Capacity During Terminal Plasma Cell Differentiation. EMBO J (2006) 25(5):1104–13. doi: 10.1038/sj.emboj.7601009 PMC1409720 16498407

[181] Hanahan D Weinberg RA. Hallmarks of Cancer: The Next Generation. Cell (2011) 144(5):646–74. doi: 10.1016/j.cell.2011.02.013 21376230

[182] Biswas SK. Metabolic Reprogramming of Immune Cells in Cancer Progression. Immunity (2015) 43(3):435–49. doi: 10.1016/j.immuni.2015.09.001 26377897

[183] Scharping NE Delgoffe GM. Tumor Microenvironment Metabolism: A New Checkpoint for Anti-Tumor Immunity. Vaccines (2016) 4(4):1–15. doi: 10.3390/vaccines4040046 PMC5192366 27929420

[184] Cassim S Pouyssegur J. Tumor Microenvironment: A Metabolic Player That Shapes the Immune Response. Int J Mol Sci (2019) 21(1):157. doi: 10.3390/ijms21010157 PMC6982275 31881671

[185] Wegiel B Vuerich M Daneshmandi S Seth P. Metabolic Switch in the Tumor Microenvironment Determines Immune Responses to Anti-Cancer Therapy. Front Oncol (2018) 8:284. doi: 10.3389/fonc.2018.00284 30151352 PMC6099109

[186] Chang CH Qiu J O’Sullivan D Buck MD Noguchi T Curtis JD. Metabolic Competition in the Tumor Microenvironment Is a Driver of Cancer Progression. Cell (2015) 162(6):1229–41. doi: 10.1016/j.cell.2015.08.016 PMC4864363 26321679

[187] Overacre-Delgoffe AE Vignali DAA. Treg Fragility: A Prerequisite for Effective Antitumor Immunity? Cancer Immunol Res (2018) 6(8):882–7. doi: 10.1158/2326-6066.CIR-18-0066 PMC6080214 30068755

[188] Price MJ Patterson DG Scharer CD Boss JM. Progressive Upregulation of Oxidative Metabolism Facilitates Plasmablast Differentiation to a T-Independent Antigen. Cell Rep (2018) 23(11):3152–9. doi: 10.1016/j.celrep.2018.05.053 PMC6092755 29898388

[189] Xiang Y Fang B Liu Y Yan S Cao D Mei H. SR18292 Exerts Potent Antitumor Effects in Multiple Myeloma *via* Inhibition of Oxidative Phosphorylation. Life Sci (2020) 256:117971. doi: 10.1016/j.lfs.2020.117971 32553925

[190] Heiden MGV Cantley LC Thompson CB. Understanding the Warburg Effect: The Metabolic Requirements of Cell Proliferation. Science (2009) 324(5930):1029–33. 10.1126/science.1160809 PMC2849637 19460998

[191] D’Souza L Bhattacharya D. Plasma Cells: You are What You Eat. Immunol Rev (2019) 288(1):161–77. doi: 10.1111/imr.12732 PMC6422051 30874356

[192] McBrayer SK Cheng JC Singhal S Krett NL Rosen ST Shanmugam M. Multiple Myeloma Exhibits Novel Dependence on GLUT4, GLUT8, and GLUT11: Implications for Glucose Transporter-Directed Therapy. Blood (2012) 119(20):4686–97. doi: 10.1182/blood-2011-09-377846 PMC3367873 22452979

[193] Stuani L Sabatier M Sarry JE. Exploiting Metabolic Vulnerabilities for Personalized Therapy in Acute Myeloid Leukemia. BMC Biol (2019) 17(1):57. doi: 10.1186/s12915-019-0670-4 31319822 PMC6637566

[194] Hui S Ghergurovich JM Morscher RJ Jang C Teng X Lu W. Glucose Feeds the TCA Cycle *via* Circulating Lactate. Nature (2017) 551(7678):115–8. doi: 10.1038/nature24057 PMC5898814 29045397

[195] Faubert B Li KY Cai L Hensley CT Kim J Zacharias LG. Lactate Metabolism in Human Lung Tumors. Cell (2017) 171(2):358–71.e9. doi: 10.1016/j.cell.2017.09.019 28985563 PMC5684706

[196] Halestrap AP PN. The Proton-Linked Monocarboxylate Transporter (MCT) Family: Structure, Function and Regulation. Biochem J (1999) 343(Pt 2):281–99. doi: 10.1042/0264-6021:3430281 PMC1220552 10510291

[197] Halestrap AP. The Monocarboxylate Transporter Family-Structure and Functional Characterization. IUBMB Life (2012) 64(1):1–9. doi: 10.1002/iub.573 22131303

[198] Fujiwara S Wada N Kawano Y Okuno Y Kikukawa Y Endo S. Lactate, a Putative Survival Factor for Myeloma Cells, is Incorporated by Myeloma Cells Through Monocarboxylate Transporters 1. Exp Hematol Oncol (2015) 4(1):1–8. doi: 10.1186/s40164-015-0008-z 25909034 PMC4407384

[199] Bolzoni M Chiu M Accardi F Vescovini R Airoldi I Storti P. Dependence on Glutamine Uptake and Glutamine Addiction Characterize Myeloma Cells: A New Attractive Target. Blood (2016) 128(5):667–79. doi: 10.1182/blood-2016-01-690743 27268090

[200] Giuliani N Chiu M Bolzoni M Accardi F Bianchi MG Toscani D. The Potential of Inhibiting Glutamine Uptake as a Therapeutic Target for Multiple Myeloma. Expert Opin Ther Target (2017) 21(3):231–4. doi: 10.1080/14728222.2017.1279148 28052702

[201] Bajpai R Matulis SM Wei C Nooka AK Von Hollen HE Lonial S. Targeting Glutamine Metabolism in Multiple Myeloma Enhances BIM Binding to BCL-2 Eliciting Synthetic Lethality to Venetoclax. Oncogene (2016) 35(30):3955–64. doi: 10.1038/onc.2015.464 PMC5025767 26640142

[202] Mayers JR Vander Heiden MG. Famine Versus Feast: Understanding the Metabolism of Tumors *In Vivo* . Trends Biochem Sci (2015) 40(3):130–40. doi: 10.1016/j.tibs.2015.01.004 PMC4340757 25639751

[203] Jin L Alesi GN Kang S. Glutaminolysis as a Target for Cancer Therapy. Oncogene (2016) 35(28):3619–25. doi: 10.1038/onc.2015.447 PMC5225500 26592449

[204] Altman BJ Stine ZE Dang CV. From Krebs to Clinic: Glutamine Metabolism to Cancer Therapy. Nat Rev Canc (2016) 16(10):619–34. doi: 10.1038/nrc.2016.71 PMC5484415 27492215

[205] Yang C Ko B Hensley CT Jiang L Wasti AT Kim J. Glutamine Oxidation Maintains the TCA Cycle and Cell Survival During Impaired Mitochondrial Pyruvate Transport. Mol Cell (2014) 56(3):414–24. doi: 10.1016/j.molcel.2014.09.025 PMC4268166 25458842

[206] Yang L Venneti S Nagrath D. Glutaminolysis: A Hallmark of Cancer Metabolism. Annu Rev BioMed Eng (2017) 19:163–94. doi: 10.1146/annurev-bioeng-071516-044546 28301735

[207] Hosios AM Hecht VC Danai LV Johnson MO Rathmell JC Steinhauser ML. Amino Acids Rather Than Glucose Account for the Majority of Cell Mass in Proliferating Mammalian Cells. Dev Cell (2016) 36(5):540–9. doi: 10.1016/j.devcel.2016.02.012 PMC4766004 26954548

[208] Hensley CT Wasti AT DeBerardinis RJ. Glutamine and Cancer: Cell Biology, Physiology, and Clinical Opportunities. J Clin Invest (2013) 123(9):3678–84. doi: 10.1172/JCI69600 PMC3754270 23999442

[209] Gaglio D Metallo CM Gameiro PA Hiller K Danna LS Balestrieri C. Oncogenic K-Ras Decouples Glucose and Glutamine Metabolism to Support Cancer Cell Growth. Mol Syst Biol (2011) 7(523):1–15. doi: 10.1038/msb.2011.56 PMC3202795 21847114

[210] DeBerardinis RJ Mancuso A Daikhin E Nissim I Yudkoff M Wehrli S. Beyond Aerobic Glycolysis: Transformed Cells can Engage in Glutamine Metabolism That Exceeds the Requirement for Protein and Nucleotide Synthesis. Proc Natl Acad Sci USA (2007) 104(49):19345–50. doi: 10.1073/pnas.0709747104 PMC2148292 18032601

[211] Le A Lane AN Hamaker M Bose S Gouw A Barbi J. Glucose-Independent Glutamine Metabolism *via* TCA Cycling for Proliferation and Survival in B Cells. Cell Metab (2012) 15(1):110–21. doi: 10.1016/j.cmet.2011.12.009 PMC3345194 22225880

[212] Jacque N Ronchetti AM Larrue C Meunier G Birsen R Willems L. Targeting Glutaminolysis has Antileukemic Activity in Acute Myeloid Leukemia and Synergizes With BCL-2 Inhibition. Blood (2015) 126(11):1346–56. doi: 10.1182/blood-2015-01-621870 PMC4608389 26186940

[213] Ni F Yu WM Li Z Graham DK Jin L Kang S. Critical Role of ASCT2-Mediated Amino Acid Metabolism in Promoting Leukaemia Development and Progression. Nat Metab (2019) 1(3):390–403. doi: 10.1038/s42255-019-0039-6 31535081 PMC6750232

[214] Thompson RM Dytfeld D Reyes L Robinson RM Smith B Manevich Y. Glutaminase Inhibitor CB-839 Synergizes With Carfilzomib in Resistant Multiple Myeloma Cells. Oncotarget (2017) 8(22):35863–76. doi: 10.18632/oncotarget.16262 PMC5482623 28415782

[215] Gonsalves WI Ramakrishnan V Hitosugi T Ghosh T Jevremovic D Dutta T. Glutamine-Derived 2-Hydroxyglutarate is Associated With Disease Progression in Plasma Cell Malignancies. JCI Insight (2018) 3(1):e94543. doi: 10.1172/jci.insight.94543 PMC5821206 29321378

[216] Gonsalves WI Jang JS Jessen E Hitosugi T Evans LA Jevremovic D. *In Vivo* Assessment of Glutamine Anaplerosis Into the TCA Cycle in Human Pre-Malignant and Malignant Clonal Plasma Cells. Cancer Metab (2020) 8(1):1–17. doi: 10.1186/s40170-020-00235-4 33308307 PMC7731537

[217] Chng WJ Huang GF Chung TH Ng SB Gonzalez-Paz N Troska-Price T. Clinical and Biological Implications of MYC Activation: A Common Difference Between MGUS and Newly Diagnosed Multiple Myeloma. Leukemia (2011) 25(6):1026–35. doi: 10.1038/leu.2011.53 PMC3432644 21468039

[218] Wise DR Deberardinis RJ Mancuso A Sayed N Zhang XY Pfeiffer HK. Myc Regulates a Transcriptional Program That Stimulates Mitochondrial Glutaminolysis and Leads to Glutamine Addiction. Proc Natl Acad Sci USA (2008) 105(48):18782–7. doi: 10.1073/pnas.0810199105 PMC2596212 19033189

[219] Osthus RC Shim H Kim S Li Q Reddy R Mukherjee M. Deregulation of Glucose Transporter 1 and Glycolytic Gene Expression by C-Myc. J Biol Chem (2000) 275(29):21797–800. doi: 10.1074/jbc.C000023200 10823814

[220] Gao P Tchernyshyov I Chang TC Lee YS Kita K Ochi T. C-Myc Suppression of miR-23a/B Enhances Mitochondrial Glutaminase Expression and Glutamine Metabolism. Nature (2009) 458(7239):762–5. doi: 10.1038/nature07823 PMC2729443 19219026

[221] Effenberger M Bommert KS Kunz V Kruk J Leich E Rudelius M. Glutaminase Inhibition in Multiple Myeloma Induces Apoptosis *via* MYC Degradation. Oncotarget (2017) 8(49):85858–67. doi: 10.18632/oncotarget.20691 PMC5689652 29156762

[222] Holien T Våtsveen TK Hella H Waage A Sundan A. Addiction to C-MYC in Multiple Myeloma. Blood (2012) 120(12):2450–3. doi: 10.1182/blood-2011-08-371567 22806891

[223] Stine ZE Walton ZE Altman BJ Hsieh AL Dang CV. MYC, Metabolism, and Cancer. Cancer Discov (2015) 5(10):1024–39. doi: 10.1158/2159-8290.CD-15-0507 PMC4592441 26382145

[224] Demel HR Feuerecker B Piontek G Seidl C Blechert B Pickhard A. Effects of Topoisomerase Inhibitors That Induce DNA Damage Response on Glucose Metabolism and PI3K/Akt/mTOR Signaling in Multiple Myeloma Cells. Am J Cancer Res (2015) 5(5):1649–64. PMC4497433 26175935

[225] Poulain L Sujobert P Zylbersztejn F Barreau S Stuani L Lambert M. High Mtorc1 Activity Drives Glycolysis Addiction and Sensitivity to G6PD Inhibition in Acute Myeloid Leukemia Cells. Leukemia (2017) 31(11):2326–35. doi: 10.1038/leu.2017.81 28280275

[226] Hardie DG. AMP-Activated Protein Kinase-an Energy Sensor That Regulates All Aspects of Cell Function. Genes Dev (2011) 25(18):1895–908. doi: 10.1101/gad.17420111 PMC3185962 21937710

[227] Saxton RA Sabatini DM. mTOR Signaling in Growth, Metabolism, and Disease. Cell (2017) 168(6):960–76. doi: 10.1016/j.cell.2017.02.004 PMC5394987 28283069

[228] Hardie DG. Molecular Pathways: Is AMPK a Friend or a Foe in Cancer? Clin Cancer Res (2015) 21(17):3836–40. doi: 10.1158/1078-0432.CCR-14-3300 PMC4558946 26152739

[229] Hu J Handisides DR Van Valckenborgh E De Raeve H Menu E Vande Broek I. Targeting the Multiple Myeloma Hypoxic Niche With TH-302, a Hypoxia-Activated Prodrug. Blood (2010) 116(9):1524–7. doi: 10.1182/blood-2010-02-269126 20530289

[230] Semenza GL. HIF-1 Mediates Metabolic Responses to Intratumoral Hypoxia and Oncogenic Mutations. J Clin Invest (2013) 123(9):3664–71. doi: 10.1172/JCI67230 PMC3754249 23999440

[231] Hay N. Reprogramming Glucose Metabolism in Cancer: Can It Be Exploited for Cancer Therapy? Nat Rev Cancer (2016) 16(10):635–49. doi: 10.1038/nrc.2016.77 PMC5516800 27634447

[232] Shim H Dolde C Lewis BC Wu CS Dang G Jungmann RA. C-Myc Transactivation of LDH-A: Implications for Tumor Metabolism and Growth. Proc Natl Acad Sci USA (1997) 94(13):6658–63. doi: 10.1073/pnas.94.13.6658 PMC21214 9192621

[233] Kim J Zeller KI Wang Y Jegga AG Aronow BJ O’Donnell KA. Evaluation of Myc E-Box Phylogenetic Footprints in Glycolytic Genes by Chromatin Immunoprecipitation Assays. Mol Cell Biol (2004) 24(13):5923–36. doi: 10.1128/MCB.24.13.5923-5936.2004 PMC480875 15199147

[234] Petrackova A Minarik J Sedlarikova L Libigerova T Hamplova A Krhovska P. Diagnostic Deep-Targeted Next-Generation Sequencing Assessment of TP53 Gene Mutations in Multiple Myeloma From the Whole Bone Marrow. Br J Haematol (2020) 189(4):e122–5. doi: 10.1111/bjh.16547 32130732

[235] Lacroix M Riscal R Arena G Linares LK Le Cam L. Metabolic Functions of the Tumor Suppressor P53: Implications in Normal Physiology, Metabolic Disorders, and Cancer. Mol Metab (2020) 33:2–22. doi: 10.1016/j.molmet.2019.10.002 31685430 PMC7056927

[236] Antonangeli F Soriani A Ricci B Ponzetta A Benigni G Morrone S. Natural Killer Cell Recognition of *In Vivo* Drug-Induced Senescent Multiple Myeloma Cells. Oncoimmunology (2016) 5(10):1–11. doi: 10.1080/2162402X.2016.1218105 PMC5087311 27853638

[237] El-Sherbiny YM Meade JL Holmes TD McGonagle D Mackie SL Morgan AW. The Requirement for DNAM-1, NKG2D, and NKp46 in the Natural Killer Cell-Mediated Killing of Myeloma Cells. Cancer Res (2007) 67(18):8444–9. doi: 10.1158/0008-5472.CAN-06-4230 17875681

[238] Paul B Kang S Zheng Z Kang Y. The Challenges of Checkpoint Inhibition in the Treatment of Multiple Myeloma. Cell Immunol (2018) 334:87–98. doi: 10.1016/j.cellimm.2018.10.003 30342750 PMC6309858

[239] Noman MZ Janji B Berchem G Chouaib S. miR-210 and Hypoxic Microvesicles: Two Critical Components of Hypoxia Involved in the Regulation of Killer Cells Function. Cancer Lett (2016) 380(1):257–62. doi: 10.1016/j.canlet.2015.10.026 26523672

[240] Duan S Guo W Xu Z He Y Liang C Mo Y. Natural Killer Group 2D Receptor and its Ligands in Cancer Immune Escape. Mol Canc (2019) 18(1):1–14. doi: 10.1186/s12943-019-0956-8 PMC6391774 30813924

[241] Riera-Domingo C Audigé A Granja S Cheng WC Ho PC Baltazar F. Immunity, Hypoxia, and Metabolism–the Ménage À Trois of Cancer: Implications for Immunotherapy. Physiol Rev (2020) 100(1):1–102. doi: 10.1152/physrev.00018.2019 31414610

[242] Barsoum IB Smallwood CA Siemens DR Graham CH. A Mechanism of Hypoxia-Mediated Escape From Adaptive Immunity in Cancer Cells. Cancer Res (2014) 74(3):665–74. doi: 10.1158/0008-5472.CAN-13-0992 24336068

[243] Paiva B Azpilikueta A Puig N Ocio EM Sharma R Oyajobi BO. PD-L1/PD-1 Presence in the Tumor Microenvironment and Activity of PD-1 Blockade in Multiple Myeloma. Leukemia (2015) 29(10):2110–3. doi: 10.1038/leu.2015.79 25778100

[244] Jelinek T Paiva B Hajek R. Update on PD-1/PD-L1 Inhibitors in Multiple Myeloma. Front Immunol (2018) 9:13. doi: 10.3389/fimmu.2018.02431 30505301 PMC6250817

[245] Yin Z Bai L Li W Zeng T Tian H Cui J. Targeting T Cell Metabolism in the Tumor Microenvironment: An Anti-Cancer Therapeutic Strategy. J Exp Clin Cancer Res (2019) 38(1):403. doi: 10.1186/s13046-019-1409-3 31519198 PMC6743108

[246] Fischer K Hoffmann P Voelkl S Meidenbauer N Ammer J Edinger M. Inhibitory Effect of Tumor Cell-Derived Lactic Acid on Human T Cells. Blood (2007) 109(9):3812–9. doi: 10.1182/blood-2006-07-035972 17255361

[247] Calcinotto A Filipazzi P Grioni M Iero M De Milito A Ricupito A. Modulation of Microenvironment Acidity Reverses Anergy in Human and Murine Tumor-Infiltrating T Lymphocytes. Cancer Res (2012) 72(11):2746–56. doi: 10.1158/0008-5472.CAN-11-1272 22593198

[248] Bohn T Rapp S Luther N Klein M Bruehl TJ Kojima N. Tumor Immunoevasion *via* Acidosis-Dependent Induction of Regulatory Tumor-Associated Macrophages. Nat Immunol (2018) 19(12):1319–29. doi: 10.1038/s41590-018-0226-8 30397348

[249] Zhang D Tang Z Huang H Zhou G Cui C Weng Y. Metabolic Regulation of Gene Expression by Histone Lactylation. Nature (2019) 574(7779):575–80. doi: 10.1038/s41586-019-1678-1 PMC6818755 31645732

[250] Seth P Csizmadia E Hedblom A Vuerich M Xie H Li M. Deletion of Lactate Dehydrogenase-A in Myeloid Cells Triggers Antitumor Immunity. Cancer Res (2017) 77(13):3632–43. doi: 10.1158/0008-5472.CAN-16-2938 PMC5505499 28446465

[251] Chiu M Toscani D Marchica V Taurino G Costa F Bianchi MG. Myeloma Cells Deplete Bone Marrow Glutamine and Inhibit Osteoblast Differentiation Limiting Asparagine Availability. Cancers (Basel) (2020) 12(11):1–17. doi: 10.3390/cancers12113267 PMC7694402 33167336

[252] Palmieri EM Menga A Martín-Pérez R Quinto A Riera-Domingo C De Tullio G. Pharmacologic or Genetic Targeting of Glutamine Synthetase Skews Macrophages Toward an M1-Like Phenotype and Inhibits Tumor Metastasis. Cell Rep (2017) 20(7):1654–66. doi: 10.1016/j.celrep.2017.07.054 PMC5575233 28813676

[253] Menga A Serra M Todisco S Riera-Domingo C Ammarah U Ehling M. Glufosinate Constrains Synchronous and Metachronous Metastasis by Promoting Anti-Tumor Macrophages. EMBO Mol Med (2020) 12(10):1–24. doi: 10.15252/emmm.201911210 PMC7539200 32885605

[254] Richardson PG Mitsiades C Hideshima T Anderson KC. Bortezomib: Proteasome Inhibition as an Effective Anticancer Therapy. Annu Rev Med (2006) 57:33–47. doi: 10.1146/annurev.med.57.042905.122625 16409135

[255] Suraweera A Münch C Hanssum A Bertolotti A. Failure of Amino Acid Homeostasis Causes Cell Death Following Proteasome Inhibition. Mol Cell (2012) 48(2):242–53. doi: 10.1016/j.molcel.2012.08.003 PMC3482661 22959274

[256] Parzych K Chinn TM Chen Z Loaiza S Porsch F Valbuena GN. Inadequate Fine-Tuning of Protein Synthesis and Failure of Amino Acid Homeostasis Following Inhibition of the ATPase VCP/P97. Cell Death Dis (2015) 6(12):e2031. doi: 10.1038/cddis.2015.373 26720340 PMC4720905

[257] Albornoz N Bustamante H Soza A Burgos P. Cellular Responses to Proteasome Inhibition: Molecular Mechanisms and Beyond. Int J Mol Sci (2019) 20(14):3379. doi: 10.3390/ijms20143379 PMC6678303 31295808

[258] Saavedra-García P Roman-Trufero M Al-Sadah HA Blighe K López-Jiménez E Christoforou M. Systems Level Profiling of Chemotherapy-Induced Stress Resolution in Cancer Cells Reveals Druggable Trade-Offs. Proc Natl Acad Sci USA (2021) 118(17):e2018229118. doi: 10.1073/pnas.2018229118 33883278 PMC8092411

[259] Fucci C Resnati M Riva E Perini T Ruggieri E Orfanelli U. The Interaction of the Tumor Suppressor FAM46C With P62 and FNDC3 Proteins Integrates Protein and Secretory Homeostasis. Cell Rep (2020) 32(12):108162. doi: 10.1016/j.celrep.2020.108162 32966780

[260] Averous J Lambert-Langlais S Mesclon F Carraro V Parry L Jousse C. GCN2 Contributes to Mtorc1 Inhibition by Leucine Deprivation Through an ATF4 Independent Mechanism. Sci Rep (2016) 6:1–10. doi: 10.1038/srep27698 27297692 PMC4906353

[261] Jin HO Hong SE Kim JY Jang SK Park IC. Amino Acid Deprivation Induces AKT Activation by Inducing GCN2/ATF4/REDD1 Axis. Cell Death Dis (2021) 12(12):1–10. doi: 10.1038/s41419-021-04417-w 34862383 PMC8642548

[262] Tsvetkov P Detappe A Cai K Keys HR Brune Z Ying W. Mitochondrial Metabolism Promotes Adaptation to Proteotoxic Stress. Nat Chem Biol (2019) 15(7):681–9. doi: 10.1038/s41589-019-0291-9 PMC8183600 31133756

[263] Besse L Besse A Mendez-Lopez M Vasickova K Sedlackova M Vanhara P. A Metabolic Switch in Proteasome Inhibitor-Resistant Multiple Myeloma Ensures Higher Mitochondrial Metabolism, Protein Folding and Sphingomyelin Synthesis. Haematologica (2019) 104(9):e415–9. doi: 10.3324/haematol.2018.207704 PMC6717569 30792209

[264] Prelowska MK Mehlich D Ugurlu MT Kedzierska H Cwiek A Kosnik A. Inhibition of the ʟ-Glutamine Transporter ASCT2 Sensitizes Plasma Cell Myeloma Cells to Proteasome Inhibitors. Cancer Lett (2021) 507:13–25. doi: 10.1016/j.canlet.2021.02.020 33713737

[265] Wang Z Liu F Fan N Zhou C Li D Macvicar T. Targeting Glutaminolysis: New Perspectives to Understand Cancer Development and Novel Strategies for Potential Target Therapies. Front Oncol (2020) 10:13. doi: 10.3389/fonc.2020.589508 33194749 PMC7649373

[266] Pompella A Visvikis A Paolicchi A De Tata V Casini AF. The Changing Faces of Glutathione, a Cellular Protagonist. Biochem Pharmacol (2003) 66(8):1499–503. doi: 10.1016/S0006-2952(03)00504-5 14555227

[267] Ballatori N Krance SM Notenboom S Shi S Tieu K Hammond CL. Glutathione Dysregulation and the Etiology and Progression of Human Diseases. Biol Chem (2009) 390(3):191–214. doi: 10.1515/BC.2009.033 19166318 PMC2756154

[268] Forman HJ Zhang H Rinna A. Glutathione: Overview of its Protective Roles, Measurement, and Biosynthesis. Mol Aspect Med (2009) 30(1–2):1–12. doi: 10.1016/j.mam.2008.08.006 PMC2696075 18796312

[269] Pallardó FV Markovic J García JL Viña J. Role of Nuclear Glutathione as a Key Regulator of Cell Proliferation. Mol Aspect Med (2009) 30(1–2):77–85. doi: 10.1016/j.mam.2009.01.001 19232542

[270] Liu RM Gaston Pravia KA. Oxidative Stress and Glutathione in TGF-β-Mediated Fibrogenesis. Free Radic Biol Med (2010) 48(1):1–15. doi: 10.1016/j.freeradbiomed.2009.09.026 19800967 PMC2818240

[271] Kaplowitz N Aw TY Ookhtens M. The Regulation of Hepatic Glutathione. Annu Rev Pharmacol Toxicol (1985) 25:715–44. doi: 10.1146/annurev.pa.25.040185.003435 3890714

[272] Meredith MJ Reed DJ. Status of the Mitochondrial Pool of Glutathione in the Isolated Hepatocyte. J Biol Chem (1982) 257(7):3747–53. doi: 10.1016/S0021-9258(18)34844-0 7061508

[273] Hwang C Sinskey AJ Lodish HF. Oxidized Redox State of Glutathione in the Endoplasmic Reticulum. Science (1992) 257(5076):1496–502. doi: 10.1126/science.1523409 1523409

[274] Yuan L Kaplowitz N. Glutathione in Liver Diseases and Hepatotoxicity. Mol Aspect Med (2009) 30(1–2):29–41. doi: 10.1016/j.mam.2008.08.003 18786561

[275] Lu SC. Regulation of Glutathione Synthesis. Mol Aspect Med (2009) 30(1–2):42–59. doi: 10.1016/j.mam.2008.05.005 PMC2704241 18601945

[276] Dalle-Donne I Rossi R Colombo G Giustarini D Milzani A. Protein S-Glutathionylation: A Regulatory Device From Bacteria to Humans. Trends Biochem Sci (2009) 34(2):85–96. doi: 10.1016/j.tibs.2008.11.002 19135374

[277] Starheim KK Holien T Misund K Johansson I Baranowska KA Sponaas AM. Intracellular Glutathione Determines Bortezomib Cytotoxicity in Multiple Myeloma Cells. Blood Cancer J (2016) 6(7):e446. doi: 10.1038/bcj.2016.56 27421095 PMC5141348

[278] Yamamoto N Sawada H Izumi Y Kume T Katsuki H Shimohama S. Proteasome Inhibition Induces Glutathione Synthesis and Protects Cells From Oxidative Stress: Relevance to Parkinson Disease. J Biol Chem (2007) 282(7):4364–72. doi: 10.1074/jbc.M603712200 17158454

[279] Zhang J Ye Z-W Chen W Culpepper J Jiang H Ball LE. Altered Redox Regulation and S-Glutathionylation of BiP Contribute to Bortezomib Resistance in Multiple Myeloma. Free Radic Biol Med (2020) 160:755–67. doi: 10.1016/j.freeradbiomed.2020.09.013 PMC7704679 32937189

[280] Townsend DM Manevich Y He L Hutchens S Pazoles CJ Tew KD. Novel Role for Glutathione S-Transferase π Regulator of Protein S-Glutathionylation Following Oxidative and Nitrosative Stress. J Biol Chem (2009) 284(1):436–45. doi: 10.1074/jbc.M805586200 PMC2610519 18990698

[281] Ye ZW Zhang J Ancrum T Manevich Y Townsend DM Tew KD. Glutathione S-Transferase P-Mediated Protein S-Glutathionylation of Resident Endoplasmic Reticulum Proteins Influences Sensitivity to Drug-Induced Unfolded Protein Response. Antioxid Redox Signal (2017) 26(6):247–61. doi: 10.1089/ars.2015.6486 PMC5312626 26838680

[282] Chen J Zaal EA Berkers CR Ruijtenbeek R Garssen J Redegeld FA. Omega-3 Fatty Acids Dha and Epa Reduce Bortezomib Resistance in Multiple Myeloma Cells by Promoting Glutathione Degradation. Cells (2021) 10(9):2287. doi: 10.3390/cells10092287 34571936 PMC8465636

[283] Wu X Xia J Zhang J Zhu Y Wu Y Guo J. Phosphoglycerate Dehydrogenase Promotes Proliferation and Bortezomib Resistance Through Increasing Reduced Glutathione Synthesis in Multiple Myeloma. Br J Haematol (2020) 190(1):52–66. doi: 10.1111/bjh.16503 32037523

[284] Xia J Zhang J Meng B Wu X Lei Q Zhu Y. High Glycine Promotes Proliferation and Progression Though Increase of Glutathione Synthesis in Multiple Myeloma. Blood (2019) 134(Supplement 1):179. doi: 10.1182/blood-2019-125452

[285] Wilkinson B Gilbert HF. Protein Disulfide Isomerase. Biochim Biophys Acta - Proteins Proteomics (2004) 1699(1–2):35–44. doi: 10.1016/S1570-9639(04)00063-9 15158710

[286] Gruber CW Čemažar M Heras B Martin JL Craik DJ. Protein Disulfide Isomerase: The Structure of Oxidative Folding. Trends Biochem Sci (2006) 31(8):455–64. doi: 10.1016/j.tibs.2006.06.001 16815710

[287] Vatolin S Phillips JG Jha BK Govindgari S Hu J Grabowski D. Novel Protein Disulfide Isomerase Inhibitor With Anticancer Activity in Multiple Myeloma. Cancer Res (2016) 76(11):3340–50. doi: 10.1158/0008-5472.CAN-15-3099 27197150

[288] Robinson RM Reyes L Duncan RM Bian H Reitz AB Manevich Y. Inhibitors of the Protein Disulfide Isomerase Family for the Treatment of Multiple Myeloma. Leukemia (2019) 33(4):1011–22. doi: 10.1038/s41375-018-0263-1 PMC7194280 30315229

[289] Desantis V Saltarella I Lamanuzzi A Mariggiò MA Racanelli V Vacca A. Autophagy: A New Mechanism of Prosurvival and Drug Resistance in Multiple Myeloma. Transl Oncol (2018) 11(6):1350–7. doi: 10.1016/j.tranon.2018.08.014 PMC6132177 30196237

[290] Frassanito MA De Veirman K Desantis V Di Marzo L Vergara D Ruggieri S. Halting Pro-Survival Autophagy by Tgfβ Inhibition in Bone Marrow Fibroblasts Overcomes Bortezomib Resistance in Multiple Myeloma Patients. Leukemia (2016) 30(3):640–8. doi: 10.1038/leu.2015.289 26487273

[291] Catley L Weisberg E Kiziltepe T Tai Y-T Hideshima T Neri P. Aggresome Induction by Proteasome Inhibitor Bortezomib and α-Tubulin Hyperacetylation by Tubulin Deacetylase (TDAC) Inhibitor LBH589 are Synergistic in Myeloma Cells. Blood (2006) 108(10):3441–9. doi: 10.1182/blood-2006-04-016055 PMC1895432 16728695

[292] Milan E Perini T Resnati M Orfanelli U Oliva L Raimondi A. A Plastic SQSTM1/p62-Dependent Autophagic Reserve Maintains Proteostasis and Determines Proteasome Inhibitor Susceptibility in Multiple Myeloma Cells. Autophagy (2015) 11(7):1161–78. doi: 10.1080/15548627.2015.1052928 PMC4590585 26043024

[293] Riz I Hawley TS Marsal JW Hawley RG. Noncanonical SQSTM1/p62-Nrf2 Pathway Activation Mediates Proteasome Inhibitor Resistance in Multiple Myeloma Cells *via* Redox, Metabolic and Translational Reprogramming. Oncotarget (2016) 7(41):66360–85. doi: 10.18632/oncotarget.11960 PMC5340085 27626179

[294] Parzych KR Klionsky DJ. An Overview of Autophagy: Morphology, Mechanism, and Regulation. Antioxid Redox Signal (2014) 20(3):460–73. doi: 10.1089/ars.2013.5371 PMC3894687 23725295

[295] Riz I Hawley TS Hawley RG. KLF4-SQSTM1/p62-Associated Prosurvival Autophagy Contributes to Carfilzomib Resistance in Multiple Myeloma Models. Oncotarget (2015) 6(17):14814–31. doi: 10.18632/oncotarget.4530 PMC4558117 26109433

[296] Rückrich T Kraus M Gogel J Beck A Ovaa H Verdoes M. Characterization of the Ubiquitin-Proteasome System in Bortezomib-Adapted Cells. Leukemia (2009) 23(6):1098–105. doi: 10.1038/leu.2009.8 19225532

[297] Balsas P Galán-Malo P Marzo I Naval J. Bortezomib Resistance in a Myeloma Cell Line is Associated to Psmβ5 Overexpression and Polyploidy. Leuk Res (2012) 36(2):212–8. doi: 10.1016/j.leukres.2011.09.011 21978467

[298] Acosta-Alvear D Cho MY Wild T Buchholz TJ Lerner AG Simakova O. Paradoxical Resistance of Multiple Myeloma to Proteasome Inhibitors by Decreased Levels of 19S Proteasomal Subunits. Elife (2015) 4:1–19. doi: 10.7554/eLife.08153 PMC4602331 26327694

[299] Shi CX Kortüm KM Zhu YX Bruins LA Jedlowski P Votruba PG. CRISPR Genome-Wide Screening Identifies Dependence on the Proteasome Subunit PSMC6 for Bortezomib Sensitivity in Multiple Myeloma. Mol Cancer Ther (2017) 16(12):2862–70. doi: 10.1158/1535-7163.MCT-17-0130 PMC5796678 28958990

[300] Tsvetkov P Mendillo ML Zhao J Carette JE Merrill PH Cikes D. Compromising the 19S Proteasome Complex Protects Cells From Reduced Flux Through the Proteasome. Elife (2015) 4:1–22. doi: 10.7554/eLife.08467 PMC4551903 26327695

[301] Tsvetkov P Sokol E Jin D Brune Z Thiru P Ghandi M. Suppression of 19S Proteasome Subunits Marks Emergence of an Altered Cell State in Diverse Cancers. Proc Natl Acad Sci USA (2017) 114(2):382–7. doi: 10.1073/pnas.1619067114 PMC5240730 28028240

[302] Parzych K Saavedra-García P Valbuena GN Al-Sadah HA Robinson ME Penfold L. The Coordinated Action of VCP/p97 and GCN2 Regulates Cancer Cell Metabolism and Proteostasis During Nutrient Limitation. Oncogene (2019) 38(17):3216–31. doi: 10.1038/s41388-018-0651-z PMC6756015 30626938

[303] Skrott Z Mistrik M Andersen KK Friis S Majera D Gursky J. Alcohol-Abuse Drug Disulfiram Targets Cancer *via* P97 Segregase Adaptor NPL4. Nature (2017) 552(7684):194–9. doi: 10.1038/nature25016 PMC5730499 29211715

[304] Bar-Natan M Stroopinsky D Luptakova K Coll MD Apel A Rajabi H. Bone Marrow Stroma Protects Myeloma Cells From Cytotoxic Damage *via* Induction of the Oncoprotein MUC1. Br J Haematol (2017) 176(6):929–38. doi: 10.1111/bjh.14493 PMC5800979 28107546

[305] Yin L Kufe T Avigan D Kufe D. Targeting MUC1-C is Synergistic With Bortezomib in Downregulating TIGAR and Inducing ROS-Mediated Myeloma Cell Death. Blood (2014) 123(19):2997–3006. doi: 10.1182/blood-2013-11-539395 24632713 PMC4014842

[306] Xu H Han H Song S Yi N Qian C Qiu Y. Exosome-Transmitted PSMA3 and PSMA3-AS1 Promote Proteasome Inhibitor Resistance in Multiple Myeloma. Clin Cancer Res (2019) 25(6):1923–35. doi: 10.1158/1078-0432.CCR-18-2363 30610101

[307] Dytfeld D Luczak M Wrobel T Usnarska-Zubkiewicz L Brzezniakiewicz K Jamroziak K. Comparative Proteomic Profiling of Refractory/Relapsed Multiple Myeloma Reveals Biomarkers Involved in Resistance to Bortezomib-Based Therapy. Oncotarget (2016) 7(35):56726–36. doi: 10.18632/oncotarget.11059 PMC5302948 27527861

[308] Luczak M Kubicki T Rzetelska Z Szczepaniak T Przybylowicz-Chalecka A Ratajczak B. Comparative Proteomic Profiling of Sera From Patients With Refractory Multiple Myeloma for Predicting Response to Bortezomib-Based Therapy. Pol Arch Intern Med (2017) 127(6):392–400. doi; 10.20452/pamw.4032 28546528

[309] Dimopoulos MA San-Miguel J Belch A White D Benboubker L Cook G. Daratumumab Plus Lenalidomide and Dexamethasone Versus Lenalidomide and Dexamethasone in Relapsed or Refractory Multiple Myeloma: Updated Analysis of POLLUX. Haematologica (2018) 103(12):2088–96. doi: 10.3324/haematol.2018.194282 PMC6269302 30237262

[310] Spencer A Lentzsch S Weisel K Avet-Loiseau H Mark TM Spicka I. Daratumumab Plus Bortezomib and Dexamethasone Versus Bortezomib and Dexamethasone in Relapsed or Refractory Multiple Myeloma: Updated Analysis of CASTOR. Haematologica (2018) 103(12):2079–87. doi: 10.3324/haematol.2018.194118 PMC6269293 30237264

[311] Sunami K Suzuki K Ri M Matsumoto M Shimazaki C Asaoku H. Isatuximab Monotherapy in Relapsed/Refractory Multiple Myeloma: A Japanese, Multicenter, Phase 1/2, Safety and Efficacy Study. Cancer Sci (2020) 111(12):4526–39. doi: 10.1111/cas.14657 PMC7734004 32975869

[312] Mikhael J Belhadj-Merzoug K Hulin C Vincent L Moreau P Gasparetto C. A Phase 2 Study of Isatuximab Monotherapy in Patients With Multiple Myeloma Who are Refractory to Daratumumab. Blood Cancer J (2021) 11(5):4–8. doi: 10.1038/s41408-021-00478-4 33980831 PMC8116334

[313] Rajkumar SV Kumar S. Multiple Myeloma Current Treatment Algorithms. Blood Cancer J (2020) 10(9):94. doi: 10.1038/s41408-020-00359-2 32989217 PMC7523011

[314] Buccarelli M D’Alessandris QG Matarrese P Mollinari C Signore M Cappannini A. Elesclomol-Induced Increase of Mitochondrial Reactive Oxygen Species Impairs Glioblastoma Stem-Like Cell Survival and Tumor Growth. J Exp Clin Cancer Res (2021) 40(1):1–17. doi: 10.1186/s13046-021-02031-4 34253243 PMC8273992

[315] Besse L Besse A Stolze SC Sobh A Zaal EA Der Ham AJV. Treatment With HIV-Protease Inhibitor Nelfinavir Identifies Membrane Lipid Composition and Fluidity as a Therapeutic Target in Advanced Multiple Myeloma. Cancer Res (2021) 81(17):4581–93. doi: 10.1158/0008-5472.CAN-20-3323 PMC7611616 34158378

[316] Driessen C Kraus M Joerger M Rosing H Bader J Hitz F. Treatment With the HIV Protease Inhibitor Nelfinavir Triggers the Unfolded Protein Response and may Overcome Proteasome Inhibitor Resistance of Multiple Myeloma in Combination With Bortezomib: A Phase I Trial (SAKK 65/08). Haematologica (2016) 101(3):346–55. doi: 10.3324/haematol.2015.135780 PMC4815726 26659919

[317] Driessen C Rouven M Novak U Cantoni N Betticher D Mach N. Promising Activity of Nelfinavir-Bortezomib-Dexamethasone in Proteasome Inhibitor – Refractory Multiple Myeloma. Lett to Blood. (2018) 132(19):2097–100. doi: 10.1182/blood-2018-05-851170 PMC6238158 30237154

[318] Chroma K Skrott Z Gursky J Bacovsky J Moudry P Buchtova T. A Drug Repurposing Strategy for Overcoming Human Multiple Myeloma Resistance to Standard-of-Care Treatment. Cell Death Dis (2022) 13(3):203. doi: 10.1038/s41419-022-04651-w 35246527 PMC8897388

[319] Federico C Alhallak K Sun J Duncan K Azab F Sudlow GP. Tumor Microenvironment-Targeted Nanoparticles Loaded With Bortezomib and ROCK Inhibitor Improve Efficacy in Multiple Myeloma. Nat Commun (2020) 11(1):6037. doi: 10.1038/s41467-020-19932-1 33247158 PMC7699624

[320] Uckun FM. Overcoming the Immunosuppressive Tumor Microenvironment in Multiple Myeloma. Cancers (Basel) (2021) 13(9):2018. doi: 10.3390/cancers13092018 33922005 PMC8122391

[321] Rasche L Wäsch R Munder M Goldschmidt H Raab MS Raab MS. Novel Immunotherapies in Multiple Myeloma - Chances and Challenges. Haematologica (2021) 106(10):2555–65. doi: 10.3324/haematol.2020.266858 PMC8485654 34196164

[322] Lakshman A Kumar SK. Chimeric Antigen Receptor T-Cells, Bispecific Antibodies, and Antibody-Drug Conjugates for Multiple Myeloma: An Update. Am J Hematol (2022) 97(1):99–118. doi: 10.1002/ajh.26379 34661922

[323] Ghobrial IM Liu CJ Redd RA Perez RP Baz R Zavidij O. A Phase Ib/II Trial of the First-in-Class Anti-CXCR4 Antibody Ulocuplumab in Combination With Lenalidomide or Bortezomib Plus Dexamethasone in Relapsed Multiple Myeloma. Clin Cancer Res (2020) 26(2):344–53. doi: 10.1158/1078-0432.CCR-19-0647 PMC11753616 31672767

[324] De Haart SJ Van De Donk NWCJ Minnema MC Huang JH Aarts-Riemens T Bovenschen N. Accessory Cells of the Microenvironment Protect Multiple Myeloma From T-Cell Cytotoxicity Through Cell Adhesion-Mediated Immune Resistance. Clin Cancer Res (2013) 19(20):5591–601. doi: 10.1158/1078-0432.CCR-12-3676 24004671

[325] SakemuraRHefaziMSieglerELCoxMJDaniel PLHansenMJ. Targeting Cancer-Associated Fibroblasts in the Bone Marrow Prevents Resistance to CART-Cell Therapy in Multiple Myeloma. Blood (2022) doi: 10.1182/blood.2021012811 35090171

